# Combined Alcohol Exposure and KRAS Mutation in Human Pancreatic Ductal Epithelial Cells Induces Proliferation and Alters Subtype Signatures Determined by Multi-Omics Analysis

**DOI:** 10.3390/cancers14081968

**Published:** 2022-04-13

**Authors:** Emalie J. Clement, Henry C.-H. Law, Fangfang Qiao, Dragana Noe, Jose G. Trevino, Nicholas T. Woods

**Affiliations:** 1 Eppley Institute for Research in Cancer and Allied Diseases, Fred & Pamela Buffett Cancer Center, University of Nebraska Medical Center, Omaha, NE 68198, USA; emalie.clement@unmc.edu (E.J.C.); henrylawch@connect.hku.hk (H.C.-H.L.); fangfang.qiao@unmc.edu (F.Q.); 2 Mass Spectrometry and Proteomics Core Facility, University of Nebraska Medical Center, Omaha, NE 68198, USA; dragana.lagundzin@unmc.edu; 3 Department of Surgery, Virginia Commonwealth University, Richmond, VA 23298, USA; jose.trevino@vcuhealth.org

**Keywords:** alcohol, pancreatic cancer, Proteomics, KRAS, SERPINE1

## Abstract

**Simple Summary:**

Pancreatic ductal adenocarcinoma is a deadly disease wherein alcohol use increases the risk of developing this cancer. Mutations in the KRAS oncogene are required for alcohol to promote pancreatic cancer in mice, but little is known about the molecular events associated with the combined exposure of alcohol and mutant KRAS expression in pancreas cells. In this study, we use pancreas cell models with and without mutant KRAS to evaluate the impact of chronic alcohol exposure on transcription and protein expression. This study identifies numerous differentially expressed transcripts and proteins that could influence the emergence of oncogenic features, such as increased proliferation, in pancreas cells.

**Abstract:**

Pancreatic Ductal adenocarcinoma (PDAC) is an aggressive cancer commonly exhibiting KRAS-activating mutations. Alcohol contributes to the risk of developing PDAC in humans, and murine models have shown alcohol consumption in the context of KRAS mutation in the pancreas promotes the development of PDAC. The molecular signatures in pancreas cells altered by alcohol exposure in the context of mutant KRAS could identify pathways related to the etiology of PDAC. In this study, we evaluated the combined effects of alcohol exposure and KRAS mutation status on the transcriptome and proteome of pancreatic HPNE cell models. These analyses identified alterations in transcription and translational processes in mutant KRAS cells exposed to alcohol. In addition, multi-omics analysis suggests an increase in the correlation between mRNA transcript and protein abundance in cells exposed to alcohol with an underlying KRAS mutation. Through differential co-expression, SERPINE1 was found to be influential for PDAC development in the context of mutant KRAS and ethanol. In terms of PDAC subtypes, alcohol conditioning of HPNE cells expressing mutant KRAS decreases the Inflammatory subtype signature and increases the Proliferative and Metabolic signatures, as we previously observed in patient samples. The alterations in molecular subtypes were associated with an increased sensitivity to chemotherapeutic agents gemcitabine, irinotecan, and oxaliplatin. These results provide a framework for distinguishing the molecular dysregulation associated with combined alcohol and mutant KRAS in a pancreatic cell line model.

## 1. Introduction

Pancreatic ductal adenocarcinoma is currently the 4th leading cause of cancer-related deaths in the United States, with a 5-year patient survival rate of only 10% due, in part, to its high rates of metastasis and treatment resistance. By the year 2030, PDAC is projected to be the 2nd leading cause of all deaths in the United States [1]. Improving the survival of PDAC patients requires a detailed understanding of the underlying molecular signatures that impact disease progression and treatment response. Toward this goal, efforts have determined that PDAC is not a singular disease and that it can be more accurately described according to several molecular subtypes described by multivariate transcriptomic, genomic, metabolomic, and proteomic signatures [2,3,4,5,6]. These subtypes are predictive of patient survival and response to chemotherapy and have the potential to optimize therapeutic interventions [5,6]. Proteomic analysis from our group identified four distinct subtypes of PDAC (Metabolic, Progenitor-like, Proliferative, and Inflammatory) utilizing patient samples with longitudinal clinical metadata [6]. This study is one of the first to identify a subtype-specific survival advantage in response to FOLFIRINOX treatment. This important discovery indicates that molecular subtyping could provide a path toward precision therapy of PDAC because subtype is predictive of therapeutic response. However, the genetic and environmental factors influencing the development of the different PDAC subtypes require additional evaluation.

The four most frequently mutated genes in PDAC are *KRAS*, *TP53*, *CDKN2A*, and *SMAD4*. Over 90% of PDACs have an activating mutation in *KRAS*. The G12D mutation in *KRAS* is the most common genetic alteration and typically precedes mutations in other genes, as it is found in early pancreatic intraepithelial neoplasia (PanIN) lesions, and subsequent mutations in the other three genes occur later in PDAC disease progression [7,8]. While KRAS is the most commonly observed mutation in PDAC, dysregulation of and mutations in other RAS/RAF/MAPK pathway members are frequently observed in other cancer types. Wild-type KRAS PDAC tumors often display activation of the MAPK pathway through BRAF mutations, suggesting KRAS and BRAF mutations can compensate for one another in the activation of the MAPK pathway [9]. Thus, PDAC is highly reliant on the activation of the MAPK pathway to drive proliferation and initiate oncogenesis.

There are several underlying causes and risk factors associated with developing PDAC, including familial history of PDAC, chronic pancreatitis, smoking, diabetes, obesity, and alcohol consumption [10], but their associations with the development of certain subtypes of the disease are poorly defined. 

Initially recognized as a co-carcinogen [11], more recent research has shown that ethanol and its associated byproducts are carcinogens. Alcohol use is a known risk factor for several cancers, including breast, stomach, esophageal, colon, liver, and PDAC cancers [11,12,13,14,15]. Chronic alcohol exposure in cancer cell models have shown that it increases tumor aggressiveness by promoting an invasive and migratory phenotype [16]. Alcohol can cause liver damage/cirrhosis, immunosuppression, vitamin deficiencies, and enhance oxidative stress, all of which can contribute to tumorigenesis [11,17].

Recent evidence indicates that alcohol intake promotes PDAC development in the context of mutated *KRAS* [18,19], which suggests that intrinsic and extrinsic factors cooperatively influence the initiation of PDAC development. Our research suggests that some risk factors, including alcohol and tobacco use, influence subtype classification [6]. A history of alcohol use impairs the frequency of presentation of the Inflammatory subtype, suggesting that alcohol may influence the development of PDAC subtypes. However, the molecular pathways impacted by alcohol use and how these contribute to subtype development remains to be determined. Therefore, in this study, we used human non-transformed human pancreatic nestin expressing (HPNE) and KRAS G12D transformed HPNE (HPNE-KRAS) cells to investigate the effects of alcohol on gene and protein expression signatures in a cell model of early PDAC development. This multi-omics study delineates the alcohol-induced proteomic and transcriptomic changes associated with cell proliferation and PDAC subtype signatures dependent upon KRAS mutation status. 

## 2. Materials and Methods

### 2.1. Cell Lines and Culture Conditions

HPNE cells were kindly provided by Dr. Ouelette [20]. HPNE-KRAS cells contain a constituently active KRAS G12D mutation [20]. HPNE and HPNE-KRAS cells were cultured in Dulbecco’s Modified Eagle Medium (DMEM; Corning, Manassas, VA, USA), supplemented with 25% M3: Base F media (INCELL, San Antonio, TX, USA), 5% Fetal Bovine Serum (FBS), human Epidermal Growth Factor (hEGF) (SigmaAldrich, St. Louis, MO, USA), penicillin-streptomycin, and Amphotericin B (Corning, Manassas, VA, USA). Cells were incubated at 37 °C with 5% CO_2_. Ethanol (EtOH)-conditioned cells (HPNE EtOH or HPNE-KRAS EtOH) were cultured in 100 mM ethanol (Calbiochem, Milipore, Billerica, MA, USA) for 6 months, as previously described [19]. Fresh ethanol was added every 2–3 days following sub culturing. 

### 2.2. Proliferation Assay

Following 6 months of ethanol treatment or untreated control, HPNE, HPNE EtOH, HPNE-KRAS and HPNE-KRAS EtOH cells were seeded at a density of 1000 cells per well in 6-well plates, for four time points. Cells for three biological replicates for each condition were manually counted with a hemocytometer every 48 h for a duration of 8 days. Cell growth rate (hour^−1^) was calculated by growth_rate = ln((N(t)/N(0))/t, where N(t) is the number of cells at the final time point, N(0) is the number of cells at time 0, and t is the final time point. The cell doubling time (hours) for each cell line was calculated by doubling_time = ln(2)/growth_rate.

### 2.3. Caspase-3 Apoptosis Assay 

Cells were lysed in CHAPS lysis buffer (1% CHAPS, 150 mM NaCl, 10 mM HEPES pH 7.4 with protease and phosphatase inhibitors) and spun down at 13,000 rpm for 10 min at 4 °C to collect the lysate. The protein concentration of the lysate was quantified and the Caspase assay buffer (1% CHAPS, 200 mM HEPES pH 7.4, 50 mM DTT, 20 mM EDTA, Caspase Substrate (N-Acetyl-Asp-Glu-Val-Asp-7-amido-4-methylcoumarin; Sigma, A1086) and cell lysates were incubated in the dark and at room temperature for 1 h in 96-well plates. The fluorescence was read at 360 nm excitation/460 nm emission by FLUOstar Optima (BMG Labtech, Cary, NC, USA). Caspase activity was calculated by subtracting wells excluding the Caspase Substrate, as previously described [21]. 

### 2.4. Cell Cycle Analysis

Ethanol-treated and untreated cells were washed 2× in PBS and harvested following trypsinization. A total of 1,000,000 cells were aliquoted for each sample condition and fixed in 70% ethanol for 1 h. Cells were washed in PBS and incubated in 1 mL Telford reagent (1 mM EDTA disodium salt, 2.5 U/mL RNAse A, 75 µM propidium iodide, 0.1% Triton X-100 in PBS) for 30 min and analyzed by flow cytometry by FACSCalibur. The data were analyzed by ModFit LT (version 4.0.5). The gating strategy can be found in Figure S1.

### 2.5. Acetaldehyde Assay

Acetaldehyde concentrations were measured by the Acetaldehyde Assay Kit (MAK321; Sigma Aldrich, St. Louis, MO, USA). Ethanol-treated and untreated cells were seeded at 1000 cells per well in a 96-well plate and incubated overnight. One hour prior to assay measurement, 100 mM ethanol was added to selected replicates. Supernatants were transferred to a new 96-well plate and the assay reaction mixes were added to the appropriate wells, following the manual’s instruction. Fluorescence was read at 530 nm/585 nm excitation/emission by FLUOstar Optima (BMG biotech, Ortenberg, Germany). 

### 2.6. Cellular ROS Measurements

Reactive Oxygen Species (ROS) were measured by the Cellular ROS Assay Kit (AbCam, ab113851). Cells were grown in 10 cm dishes in HPNE media (20,000 cells per condition) and were aliquoted and incubated in 2′,7′-dichlorofluorescin diacetate (DCFDA) for 30 min at 37 °C to stain cellular ROS. Following incubation, cells were treated with or without ethanol. Positive control cells were treated with tert-butyl hydrogen peroxide (THBP) for 3 h to induce ROS production and allow an appropriate time to acquire an optimal signal. DCFDA staining was omitted for the negative control cells. Cellular ROS staining was analyzed by flow cytometry (488 nm excitation, 535 nm detection) by FACSCalibur and the data were analyzed by ModFit LT (version 4.0.5). The gating strategy can be found in Figure S2. 

### 2.7. RNA Sequencing (RNA-Seq)

Ethanol-treated and untreated cells were grown in 10 cm dishes. For each condition, 1 × 10^6^ cells were collected following trypsinization. The Qiagen RNeasy Plus Mini kit (Qiagen, Hilden, Germany) was used to extract total cellular RNA via the manual’s instructions. RNA quality was checked with a Fragment Analyzer prior to library preparation and sequencing. Libraries were generated using 1000 ng of total RNA from each sample and the TrueSeq V2 RNA-Seq kit (Illumina Inc., San Diego, CA, USA), following the recommended procedure. Libraries were multiplexed and sequenced on the NextSeq550 DNA Analyzer (Illumina) to generate a total of approximately 12 million pairs of 75 bp reads for each sample. FastQC was used to ensure the quality of the reads were adequate for alignment and quantification. STAR was used to align sequencing reads to the human genome primary assembly (GRCh38) downloaded from Ensembl, with Ensembl annotation. Read counts were determined by HTSeq using the default union parameter. A pseudocount of one was added to all raw transcript counts, and normalization factors were calculated and applied to the data with the size.factor() function through DESeq2 in R (median of ratios method). 

### 2.8. Peptide Preparation

For protein extract preparation, cells were washed with PBS, lysed with RIPA lysis buffer, and briefly sonicated. Protein concentrations were measured with a BCA protein assay (Pierce, ThermoFisher Scientific, Rockford, IL, USA, Cat#: 23227) using BSA as a standard. Briefly, protein lysates were reconstituted in 100 mM triethylammonium bicarbonate (TEAB) and incubated with 100 mM Tris(2-carboxyethyl)phosphine (TCEP) at 55 °C for 1 h. The samples were subsequently incubated in 375 mM iodoacetamide for 30 min, protected from light, and then acetone precipitated, and trypsin digested (2.5 ug Trypsin) overnight at 37 °C, as previously described [22].

### 2.9. TMT Tagging 

Tryptic peptide concentrations were measured with the Thermo Colorimetric Peptide Assay (Pierce, ThermoFisher Scientific, Rockford, IL, USA, Cat#: 23275) and incubated with anhydrous acetonitrile-dissolved TMT reagents (ThermoFisher Scientific, Rockford, IL, USA, TMT10 Plex Ref 90309 Lot TE264412) for 1 h at room temperature. After quenching the reaction with hydroxylamine, the samples were combined and fractionated with Pierce High pH Reversed-Phase Peptide Fractionation Kit (ThermoFisher Scientific, Cat#: 84868).

### 2.10. LC/MS-MS and Protein Identification

Peptides generated by typsin digest were analyzed by LC-MS/MS on a Dionex Nano Ultimate 3000 RSLCnano system (ThermoFisher Scientific) coupled to an Orbitrap Fusion Lumos mass spectrometer (ThermoFisher Scientific). The samples were first injected onto a trap column (Acclaim PepMap™ 100, 75 µm × 2 cm, ThermoFisher Scientific) for 3 min at a flow rate of 4 µL/min before switching in line with the main column. Separation was performed on a C18 nano column (EASY-Spray™, 2 µm 75 µm × 500 mm, ThermoFisher Scientific) at 300 nL/min with a linear gradient from 0–45% over 120 min. The LC aqueous mobile phase contained 0.1% (*v*/*v*) formic acid in water and the organic mobile phase contained 0.1% (*v*/*v*) formic acid in 80% (*v*/*v*) acetonitrile. Mass spectra for the eluted peptides were acquired on a Fusion Lumos mass spectrometer in data-dependent mode using a mass range of m/z 375–1500, resolution 120,000, AGC target: standard, maximum injection time: 150 ms. Data-dependent MS2 spectra were acquired by CID, collision energy: 35%, AGC target: standard, maximum injection time: auto, isolation window: 2 m/z. MS3 spectra were acquired with HCD activation, HCD collision energy: 65%, Orbitrap resolution: 50,000, normalized AGC target: 200%. 

Database searching: The mass spectra were extracted using Proteome Discoverer (2.1.1.21). All MS/MS samples were analyzed using Sequest HT. Sequest HT was set up to search the SwissProt database (SwissProt TaxID = 9606 v2017-10-25, 42,252 entries) assuming the digestion enzyme trypsin allowing for up to 2 missed protease cleavage sites. Sequest HT was searched with a fragment ion mass tolerance of 0.60 Da and a parent ion tolerance of up to 10.0 PPM. TMT6plex/+229.163 Da (on any N-Terminus), and carbamidomethyl of cysteine were specified as fixed modifications. Oxidation of methionine and TMT6plex/+229.163 Da of Lysine were specified as variable modifications. False discovery rates were determined by searching the decoy database. 

### 2.11. Bioinformatics and Statistical Analysis

#### 2.11.1. Differential Expression

Following DESeq2 normalization, RNA-Seq transcripts were categorized by biotype (Ensembl). Transcripts in the EtOH-treated conditions that were ±2-fold from the untreated control cell line were considered differentially expressed to ensure confidence in transcript expression changes with single replicates for each condition. For the MA plot, all transcript biotypes were included. Log2-fold changes were calculated for EtOH-treated compared to the untreated control and the average of each transcript was calculated including all conditions. For the Venn diagram, only transcripts that were determined to be protein-coding transcripts were included. 

For proteomics values, the median expression values of the ion reporter abundance ratio values were used for comparing the fold change of the EtOH-treated and untreated control cells. A fold change of ±1.25 between the EtOH-treated and untreated cells was used to determine differentially expressed proteins. Statistical significance was calculated by Students T-test and the adjusted *p*-values (using the false-discovery rate (FDR)) were calculated in R with the p.adjust() function.

R was used to generate a hierarchical clustering heatmap for the RNA Seq and proteomics data set. The Euclidian distance was calculated, and clustering was performed by the hclust function in R (hclust complete clustering). The number of clusters was determined from the elbow plots for each data set. For the Principal Components Analysis, HPNE replicate 2 was omitted as an outlier based on falling outside of the Hotelling Ellipse (95% confidence interval). 

#### 2.11.2. GO Term Analysis

GO term analysis for RNA Seq and proteomics data was done through DAVID Bioinformatics using the filtered GO FAT ontology terms (no background set was included). Differentially expressed proteins and genes with a ±1.25 and ±2.0-fold change were used for GO term enrichment, respectively, including genes/proteins, regardless of significance. GO bubble plots were generated in R with the GOplot package. The z-score was calculated by (up/down)/sq(count), where up and down are the number of assigned genes upregulated or downregulated in the data, and count is the number of genes assigned to a term. All genes and proteins for the highest transcript changes and SERPINE1 were used as input for GO term analysis, through DAVID Bioinformatics using the filtered GO FAT ontology terms.

#### 2.11.3. Multiple Co-Inertia Analysis

Multiple Co-Inertia Analysis was done in R with the omicade4 package. The 3247 unique proteins from the proteomics data were cross referenced with the 23,190 transcripts from the RNA Seq data set. A total of 3181 proteins matched, with the values found in the RNA Seq data set used for input into omicade4. 

#### 2.11.4. Differential CoExpression

To investigate which proteins were coordinately expressed in the HPNE and how ethanol and mutant KRAS altered coordinately expressed proteins, we performed a differential co-expression analysis. Proteins with any missing value were excluded from the analysis. Pearson correlation coefficients for each protein–protein pair were calculated for each condition separately using the psych::corr.test in R. Correlations that were greater than 0.75 in HPNE, HPNE EtOH, and HPNE-KRAS, but less than −0.75 in HPNE-KRAS EtOH were considered for the negative correlation network, while correlations that were less than −0.75 in HPNE, HPNE EtOH, and HPNE-KRAS, but greater than 0.75 in HPNE-KRAS EtOH were considered for the positive correlation network. Networks were generated in Cytoscape 3.7.2.

#### 2.11.5. TCGA Data

SERPINE1 protein expression data from PDAC patients from the TGCA Firehose Legacy was accessed through the cBioportal [23,24]. Data for the Kaplan Meier survival analysis curves was accessed through the Human Protein Atlas [25] (https://www.proteinatlas.org/ENSG00000106366-SERPINE1/pathology/pancreatic+cancer (accessed on 10 December 2021) and plotted in R using the survival and survminer packages. 

### 2.12. Quantitative Reverse Transcription Polymerase Chain Reaction (qRT-PCR)

RNA from each cellular condition was extracted with the Qiagen RNeasy Plus Mini kit (Qiagen, Hilden, Germany; Cat#: 74134). In addition, 1 µg of RNA was reverse transcribed with the iScript cDNA Synthesis Kit (BioRad, Hercules, CA, USA) and 100 ng of cDNA was used with iTaq Universal SYBR Green Supermix for gene quantification by the CFX96 Real time PCR detection system (BioRad). Experimental procedures were performed according to the manufacturer’s instructions. Relative gene expression and Cq values were determined by the CFX Manager Software. Values are represented as mean delta Cq to show the relative change across both cell lines and all conditions. GAPDH was used as a control for normalization. Primers (listed 5′-3′): CD81 forward: 5′-ATTTCGTCTTCTGGCTGGCTG-3′, CD81 reverse: 5′-TATACACAGGCGGTGATGGC-3′, NT5E forward: 5′-GTATCCGGTCGCCCATTGAT-3′, NT5E reverse: 5′-AAAGGCCTTCTTCAGGGTGG-3′, PRPF19 forward: 5′-TGCCAAGTTCCCAACCAAGT-3′, PRPF19 reverse: 5′-ATTGGGGACCGACCAAATCC-3′, RHOG forward: 5′-GCCTGCTCATCTGCTACACA-3′, RHOG reverse: 5′-CTCTGCGCGCTGTAATTGTC-3′, RUVBL1 forward: 5′-AGAGCACTACGAAGACGCAG-3′, RUVBL1 reverse: 5′-GCGCATTAGCCACATCCAAG-3′, ZPR1 forward: 5′-GGAACAACACGGAGATCCAG-3′, ZPR1 reverse: 5′-CTCGCAGTTGGTAGCCATGA-3′.

### 2.13. Western Blotting

Cells were washed with PBS, lysed with RIPA lysis buffer, and put on ice. Protein concentrations were measured with the NanoDrop One Microvolume UV Spectrophotometer (ThermoFisher) A280 Protein measurement. Protein lysates were run on SDS-PAGE gels and transferred to PVDF membranes. Membranes were blocked for 30 min with Licor blocking buffer and incubated with 1:1000 primary antibody dilutions in 2.5% BSA overnight. Membranes were washed 3× in TBS-T and incubated with 1:4000 HRP- conjugated secondary antibodies for 1 h and developed with SuperSignal West Femto Maximum Sensitivity Substrate (ThermoFisher, Cat. No. 34096). Blots were scanned with the Odyssey Fc imager (Li-Cor) and signal intensity was analyzed with Image Studio (Li-Cor, version 5.2). Antibodies used: CD81 (Santa Cruz Biotechnology, Dallas, TX, USA, sc-166029), NT5E (Cell Signaling Technology, Danvers, MA, USA, 13160S), PRPF19 (Santa Cruz Biotechnology, sc-514338), RHOG (Santa Cruz Biotechnology, sc-80015), RUVBL1 (Cell Signaling Technology, 12300S), ZPR1 (Santa Cruz Biotechnology, sc-398241), GAPDH (Cell Signaling Technology, 5174T), HSC70 (Santa Cruz Biotechnology, sc-7298).

### 2.14. Chemotherapeutic Agent Treatment and Viability Assay

HPNE, HPNE EtOH, and HPNE-KRAS cells were plated at a density of 5000 cells per well in a 96-well plate. HPNE-KRAS EtOH cells were plated at 1000 cells per well (due to the high proliferation rate, the wells were 100% confluent by day 2, plated at 5000 cells per well, and complicated cell viability measurements). Cells were allowed to adhere overnight, and the drug was added at 24 h. Plates were incubated at 37 °C, 5% CO_2_ for 3 days. Cell viability was measured by CellTiter-Glo 2.0 (Promega, Madison, WI, USA, G9242), as indicated by the instruction manual. Luminescence was read by the FLUOstar Optima (BMG biotech). Gemcitabine (cat#: S1714), Irinotecan HCl Trihydrate (cat#: S2217), and Oxaliplatin (cat#: S1224) were purchased from Selleckchem (Houston, TX, USA).

## 3. Results

### 3.1. Chronic Ethanol Treatment Promotes Proliferation in KRAS Mutated HPNE Cells 

**Figure 1:**
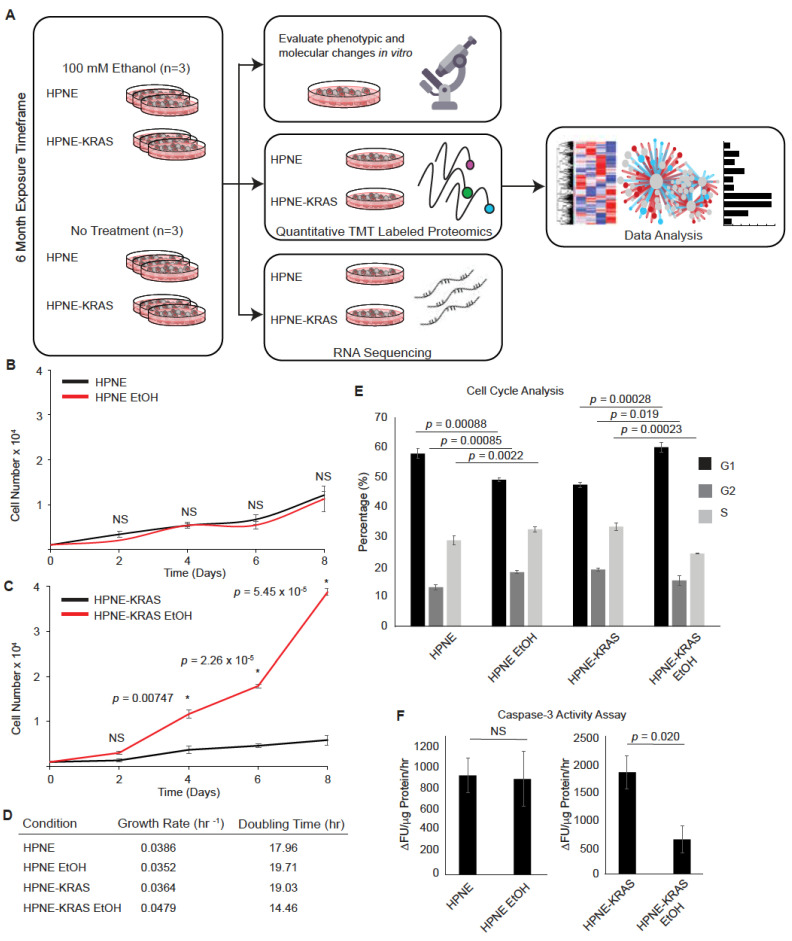
Ethanol promotes HPNE cell population expansion in a KRAS-dependent manner. (**A**) Schematic of the study design. RNA-Seq and quantitative proteomics were used to characterize the impact of EtOH conditioning in the context of KRAS mutations in non-transformed pancreatic HPNE cells. (**B**,**C**) Cell population growth curves. In total, 1000 cells were plated per well and counted every 2 days over an 8-day period for HPNE (**B**) and HPNE-KRAS (**C**) cell lines. Mean ± standard error of the mean (SEM); *n* = 3; significant *p*-values determined by Student’s *t*-test are indicated; NS, not significant; *, *p*-value ≤ 0.05. (**D**) Cell population doubling time determined from the cell counting study depicted in (**B**,**C**). (**E**) Cell cycle profiles were determined by PI staining and FACs analysis for the indicated cell lines and treatments. Mean ± SEM; *n* = 3; *p*-value determined by Student’s *t*-test. (**F**) The steady-state levels of Caspase-3 activity in the indicated cells and treatments represented as the change in fluorescent units per µg whole protein lysate per 1 h reaction time (∆FU/µg protein/hr). Mean ± SEM; *n* = 3.

Non-transformed human pancreatic nestin expressing (HPNE) and mutant KRAS G12D-transformed HPNE (HPNE-KRAS) cells were used in this study to evaluate the impact of alcohol in a defined cellular and genetic context relevant to the most common early mutation event in pancreatic cancer [20]. Alcohol use by humans is difficult to model because of the variability of behaviors between individuals. Alcohol exposure occurs over the course years and decades and may affect molecular, cellular, or tissues through either chronic and/or acute exposures. To model a lifetime worth of alcohol exposure in a short period of 6 months, we treated the HPNE and HPNE-KRAS cells with 100 mM EtOH every 2–3 days as previously described [26]. This allowed us to examine the effect of ethanol exposure on wild-type KRAS and constitutively active KRAS (G12D) expressing pancreas cells. After 6 months of EtOH treatment, phenotypic changes in cell proliferation were evaluated and a multi-omics analysis of the proteome and transcriptome was performed (Figure 1A).

Both HPNE and HPNE-KRAS cells tolerated the ethanol exposure and significant cell death was not observed during the treatment period. Six months after beginning ethanol treatment, the HPNE cells did not exhibit any difference in proliferation between the control and EtOH treatment conditions (Figure 1B). However, the HPNE-KRAS cells treated with EtOH displayed a significant increase in their proliferation rate compared to control cells (Figure 1C). Analysis of the doubling times of these cells in culture confirmed EtOH treatment promotes a faster growth rate in HPNE-KRAS compared to control (Figure 1D). The increased proliferation in EtOH-treated HPNE-KRAS cells was associated with an increase in the percentage of cells in the G1 phase, with lower numbers in the G2 and S phases, compared to the un-treated HPNE-KRAS control cells. Whereas, in HPNE cells treated with EtOH, there is a decrease in cells in G1 phase and an increase in cells in the G2 and S phases, compared to the untreated HPNE cells (Figure 1E and Figure S1). 

The rapidly expanding cell population observed in the ethanol-conditioned HPNE-KRAS cell culture could also be influenced by changes in the rates of cell death. Chronic ethanol treatment has been shown to induce caspase-3 activation and apoptosis in various non-transformed cell types, including β-cells and T-cells [27,28,29,30,31]. In our experiments, there was no difference in caspase-3 activity between control and EtOH-conditioned HPNE cells. However, EtOH-conditioned HPNE-KRAS cells displayed a decrease in caspase-3 activity in steady-state cultures (Figure 1F). Together, these results suggest that changes in the cell cycle and decreased apoptotic rates contribute to the decreased population doubling time of EtOH-treated HPNE-KRAS cells.

### 3.2. Mutant KRAS and Ethanol Treatment Increases Ribosomal and RNA Metabolism Gene Expression

**Figure 2:**
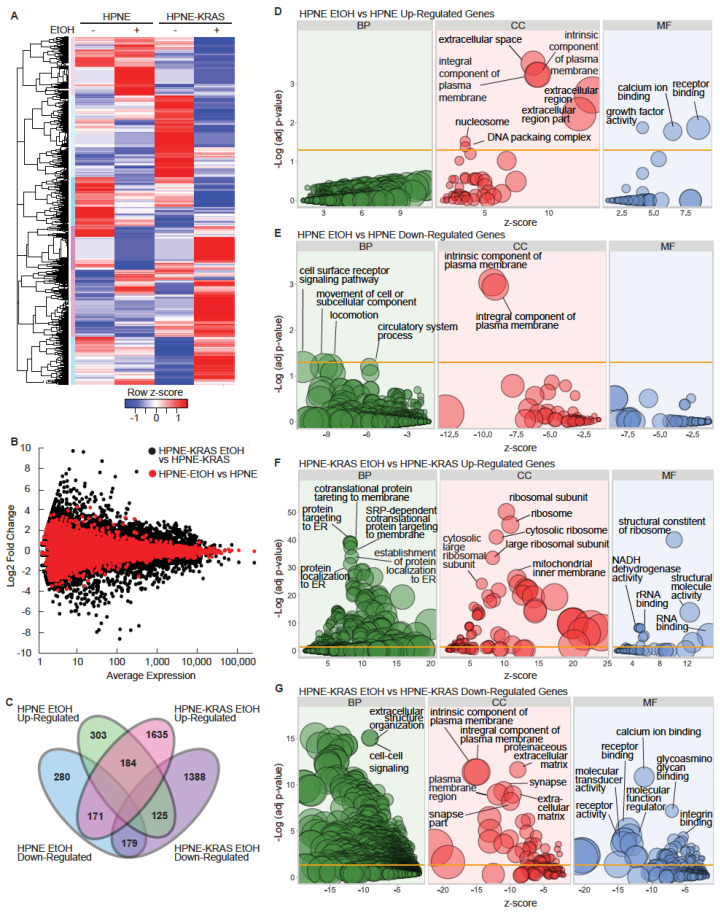
EtOH and KRAS status influence gene expression profiles in HPNE cells. (**A**) Heatmap of transcript expression values measured by RNA-Seq. (*n* = 1). (**B**) MA plot showing the log ratio (M) versus the mean expression (**A**), across all conditions, of transcripts detected by RNA-Seq. (**C**) Venn diagram of differentially expressed protein coding transcripts. Protein coding transcripts that were ±2-fold different in the EtOH-conditioned cells compared to the untreated controls were considered differentially expressed. (**D**–**G**) Ethanol-conditioned cell lines were compared to untreated controls to identify up and downregulated protein coding genes (fold change = ±2) for GO term enrichment analysis. The DAVID bioinformatics tool was used to identify enriched terms for up or downregulated genes (analyzed independently). The z-score (x-axis) was calculated by z-score = (up − down)/sq (count), where up and down are the number of assigned genes upregulated or downregulated in the data, and count is the number of genes assigned to a term. The bubble size is representative of the number of genes present in the respective term. FDR was used for the adjusted *p*-values.

RNA-Seq analysis was performed on the HPNE cell lines to identify gene expression signatures affected by mutant KRAS and EtOH exposure, which identified 6 clusters of similarly expressed genes (Figure 2A and Figure S3A; Table S1). Larger fold-change differences in gene expression due to EtOH conditioning were observed in the context of HPNE-KRAS mutant cells in comparison to wild-type HPNE cells (Figure 2B). HPNE-KRAS cells also exhibit a larger number of differentially expressed transcripts (±2-fold change) associated with EtOH conditioning (7017 transcripts), compared to the HPNE cells (3105 transcripts) (Figure S3B). In protein coding genes only, there were 997 genes with a fold change of 2 or greater, while 728 had a fold change of −2 or lower (Table S2). There were 184 commonly upregulated genes and 179 commonly downregulated genes (±2-fold changes) in both HPNE EtOH and HPNE-KRAS EtOH cells (Figure 2C), suggesting conserved influence of EtOH conditioning on the regulation of these genes independent of KRAS mutation status. However, 1635 upregulated (Table S3, Figure 2C) and 1388 downregulated (Table S4, Figure 2C) genes were uniquely associated with EtOH conditioning in HPNE-KRAS cells compared to 303 upregulated and 280 downregulated genes unique to EtOH conditioning in HPNE cells (Figure 2C). Together, these results suggest that an underlying KRAS mutation in pancreatic cells may exacerbate the dysregulation of gene expression caused by prolonged ethanol exposure.

To evaluate the common characteristics of these differentially expressed protein coding genes, gene ontology (GO) term enrichment analysis was performed using DAVID [32,33] for both the HPNE (Figure 2D,E) and HPNE-KRAS (Figure 2F,G) cell lines. The upregulated gene set associated with EtOH conditioning in HPNE cells is enriched for cellular component (CC) terms corresponding to the extracellular region and components of the plasma membrane, while enriched molecular function (MF) terms for receptor binding, calcium ion binding, and growth factor activity were identified (Figure 2D, Table S5). There were no significantly enriched biological process (BP) terms for the gene set upregulated by EtOH in HPNE cells. Enriched GO terms in the downregulated genes associated with EtOH conditioning in HPNE cells were cell surface receptor signaling, movement of cell or subcellular component in the BP category, and plasma membrane components in the CC category (Figure 2E, Table S6), similar to the results observed in the upregulated gene set (Figure 2D). In EtOH-conditioned HPNE-KRAS cells, upregulated genes were enriched in protein targeting to the plasma membrane and ER terms in the BP category, ribosomal component terms in the CC category, and rRNA metabolism/processing terms in the MF category (Figure 2F, Table S7). Genes downregulated in HPNE-KRAS ethanol treated cells were enriched in extracellular matrix and structure organization and cell–cell signaling (BP); plasma membrane and extracellular matrix components, neuron, and synapse parts (CC); calcium ion binding, receptor binding and activity, and glycosaminoglycan binding (MF) (Figure 2G, Table S8). The combined impact of KRAS mutation and EtOH conditioning on ribosomal gene expression suggested that protein expression profiles could be affected.

### 3.3. Mutant KRAS and Ethanol Treatment Increases Ribosomal and RNA Processing Proteins

**Figure 3:**
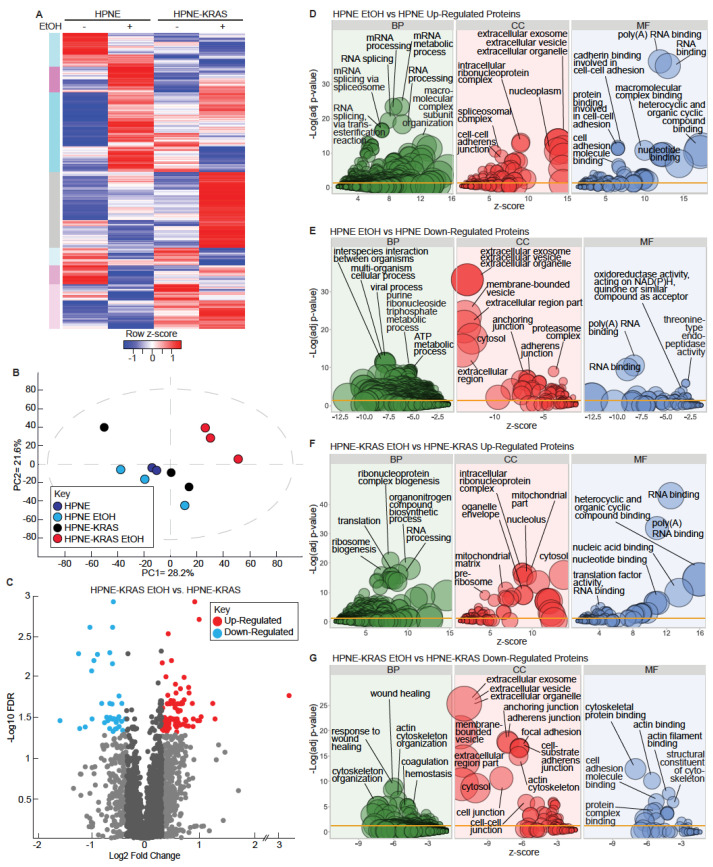
Quantitative proteomic analysis of the impact of EtOH and KRAS mutation in HPNE cells on protein expression. (**A**) Heatmap of the mean protein expression values for HPNE and HPNE-KRAS cells treated with or without EtOH. *n* = 3. (**B**) Principal Components Analysis (PCA) of the proteomics data for HPNE and HPNE-KRAS cells with or without EtOH conditioning. HPNE-2 was removed as an outlier. (**C**) Volcano plot of protein expression differences between HPNE-KRAS EtOH vs. HPNE-KRAS (−log_10_ *p*-value, determined by Student’s *t*-test). (**D**–**G**) Ethanol-conditioned cell lines were compared to untreated controls to select up and downregulated proteins (cutoff, fold-change ±1.25) for GO term enrichment analysis for the indicated comparisons. The DAVID bioinformatics tool was used to identify enriched terms for up or downregulated proteins (analyzed independently). The z-score (x-axis) was calculated by z-score = (up − down)/sq (count), where up and down are the number of assigned proteins upregulated or downregulated in the data, and count is the number of proteins assigned to a term. The bubble size is representative of the number of proteins present in the respective term. FDR was used for the adjusted *p*-values.

To characterize the protein-level effects of EtOH and KRAS mutation in HPNE cells, we used quantitative shotgun proteomics with 3 biological replicates for each condition. We detected 3861 proteins. Proteins that had at least 2 of 3 replicate values in each condition were included for further analysis. A total of 3167 and 3210 proteins met these requirements in the HPNE and HPNE-KRAS cells, respectively (Figure 3A, Tables S9 and S10). Principal components analysis (PCA) was used to visualize the variations and patterns in the proteome of each sample (Figure 3B). EtOH treatment in the HPNE cell line had a relatively minor effect on the variation of the proteome in comparison to the untreated HPNE cells. However, EtOH treatment in the context of mutant KRAS expression had a larger effect on the variation of the proteome compared to non-treated HPNE-KRAS control cells (Figure 3B). Like the RNA-seq results, EtOH conditioning had a larger effect on differential protein expression in HPNE-KRAS cells compared to HPNE. At a fold-change threshold of ±1.25 and an FDR ≤ 0.05 to define differentially expressed proteins, 132 proteins (95 up-regulated and 37 down-regulated) were differentially expressed in the HPNE-KRAS EtOH as compared to the non-treated HPNE-KRAS cells (Figure 3C, Table S10); however, no proteins met this FDR threshold in HPNE cells (Table S9).

We performed GO term enrichment analysis through DAVID to determine significant terms associated with differentially expressed proteins (±1.25-fold change) in both the HPNE and HPNE-KRAS cell lines (Figure 3D–G). Biological process (BP) terms enriched in HPNE EtOH up-regulated proteins were primarily associated with RNA processing and splicing, cellular component (CC) terms included ribonucleoprotein and spliceosomal complex, molecular function terms (MF) included cadherin binding involved in cell-cell adhesion, and poly(A) and RNA binding (Figure 3D, Table S11). The top terms for with the down-regulated proteins in HPNE cells treated with EtOH were cell interspecies interaction between organisms (BP) extracellular organelle (CC), oxidoreductase, and threonine-type endopeptidase activity (MF) (Figure 3E, Table S12). Poly(A) and RNA binding were also significant terms associated with both down-regulated proteins and up-regulated proteins. Like the RNA Seq data, we observed more enriched terms in HPNE-KRAS EtOH up- and down-regulated proteins for all three GO categories (Figure 3F,G). Up-regulated proteins were enriched in protein targeting to the ER and ribosomes; functions related to translation, rRNA/RNA processing and binding appeared frequently in the GO term results (Figure 3F, Table S13). Downregulated genes were enriched in extracellular structure organization, components of the plasma membrane, and calcium ion binding (Figure 3G, Table S14). 

### 3.4. Ethanol Metabolism Is Marginally Impacted Following Chronic Ethanol Conditioning

**Figure 4:**
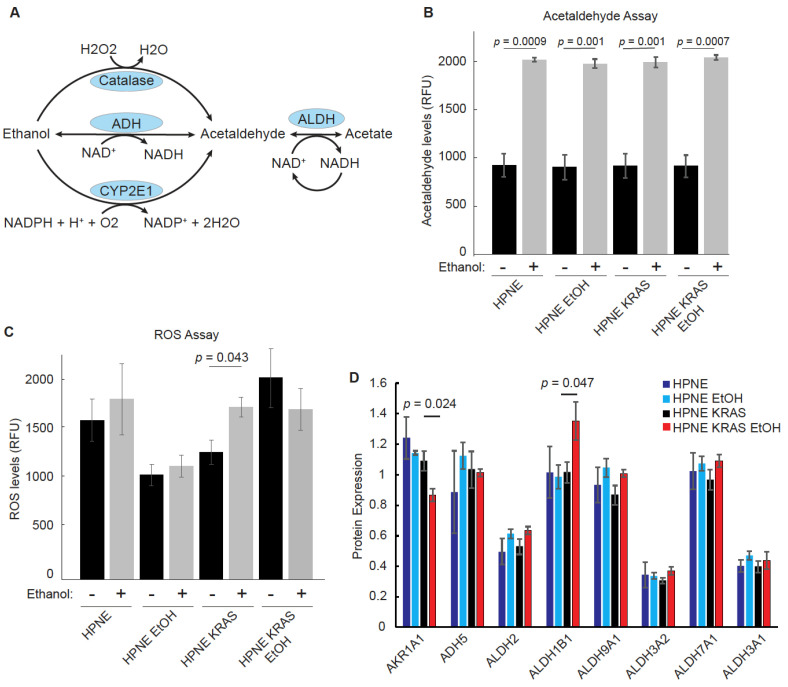
The effects of EtOH conditioning on ethanol metabolism in HPNE and HPNE-KRAS cells. (**A**) Simplified depiction of the ethanol metabolic pathway. Metabolites are in black text; enzymes are in blue circles. (**B**) Measurement of acetaldehyde abundance with and without acute EtOH exposure for 1 h in both the parental- and EtOH-conditioned HPNE and HPNE-KRAS cell lines. Mean ± SEM; *n* = 3. (**C**) Cellular Reactive Oxygen Species (ROS) measurements by DCFDA staining for the indicated cell line and treatment condition. Mean ± SEM; *n* = 3. (**D**) Protein expression of ethanol breakdown metabolism enzymes detected by quantitative TMT labeled proteomics. Mean ± SEM; *n* = 3. *p*-values were determined by Student’s *t*-test.

The initial steps of ethanol breakdown involve alcohol dehydrogenase (ADH) and cytochrome P450 2E1 (CYP2E1) that generate acetaldehyde from ethanol [34]. ADH has a low Km (Michaelis constant) for ethanol and is the most active enzyme in metabolizing ethanol [35,36]. Under conditions of high ethanol concentrations, however, ethanol can be metabolized by CYP2E1, which generates ROS as a byproduct of the reaction, potentially causing damage to DNA and proteins that contribute to cancer progression (Figure 4A). Therefore, we measured the baseline acetaldehyde and ROS levels in the HPNE and HPNE-KRAS that were conditioned with EtOH compared to similar passage untreated control cells and their response to acute EtOH stimulation (100 mM incubated for 1 h prior to measurement). While acute EtOH stimulation increased the production of acetaldehyde, no significant differences in acetaldehyde concentrations were observed between the different cell lines, regardless of EtOH conditioning status (Figure 4B). Similarly, ROS levels were not drastically altered across the different cell lines, except for the non-EtOH- conditioned HPNE-KRAS cells that demonstrated an increase in the ROS levels following acute EtOH stimulation (Figure 4C, Figure S2). The lack of significant changes in acetaldehyde and ROS production is also reflected in the proteomic data. Most of the enzymes associated with EtOH metabolism detected in the proteomics experiments are expressed at similar levels in both HPNE and HPNE-KRAS cells and not affected by EtOH conditioning (Figure 4D). The exception to this being of AKR1A1 and ALDH1B1 that were down-regulated or upregulated, respectively, in EtOH-conditioned HPNE-KRAS cells (Figure 4D). Notably, ALDH1B1 and mutant KRAS have been implicated in driving PDAC development [37]. Together, these results suggest that the changes in phenotype associated with EtOH conditioning in HPNE-KRAS cells is likely not due to adaptations associated with EtOH metabolic pathways.

### 3.5. Mutant KRAS Cells Treated with Ethanol Display Increased Correlation between RNA and Protein Expression through Multi-Omics Comparison

**Figure 5:**
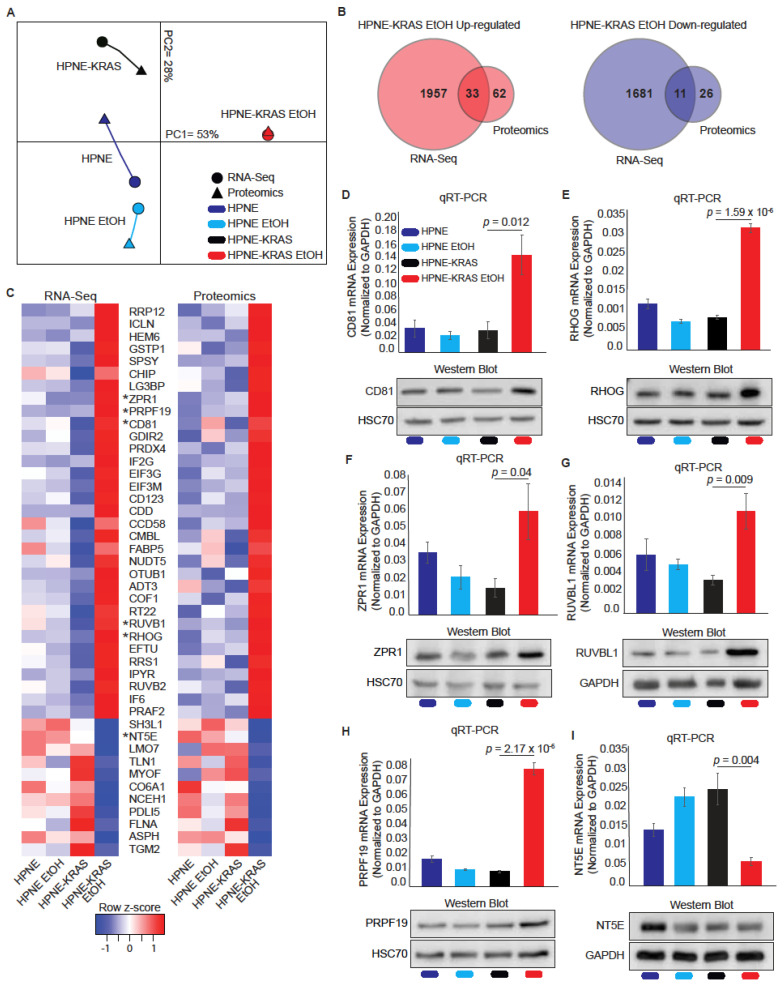
Mutant KRAS cells treated with ethanol display a higher level of transcriptional and translational coordination. (**A**) Multiple Co-inertia analysis of RNA-Seq and proteomics data. Length of the line connecting the proteomics and RNA-Seq data represents their dissimilarity; RNA-Seq *n* = 1, proteomics *n* = 3. (**B**) Venn diagrams showing differentially expressed protein coding genes (RNA-Seq, ±2-fold change) and proteins (Proteomics, ±1.25-fold change) common in the RNA-Seq and proteomics data. (**C**) Heatmaps of all conditions showing expression of commonly detected up-(33) and down-(11) regulated protein-coding transcripts (left) and proteins (right) found in HPNE-KRAS EtOH cells compared to HPNE-KRAS cells. (**D**–**I**). Validation of omics data by qRT-PCR and Western blot for CD81 (**D**), RHOG (**E**), ZPR1 (**F**), RUVBL1 (**G**), PRPF19 (**H**), and NT5E (**I**); indicated in (**C**) by *. The qRT-PCR was performed to validate RNA-Seq expression with *GAPDH* used for normalization. Mean ± SEM; *n* = 4. Western blots were used to validate proteomics expression using HSC70 or GAPDH as loading control. Cluster of Differentiation 81 (CD81); Ras Homolog Family Member G (RHOG); ZPR1 zinc finger (ZPR1); RuvB like AAA ATPase 1 (RUVBL1); Pre-MRNA Processing Factor 19 (PRPF19); 5′-Nucleotidase (NT5E). The original blots of Figure 5 could be found in Figure S5.

Multiple co-inertia analysis (MCIA) was performed to observe the relationship between the RNA-Seq and proteomics data for each sample. The RNA-Seq and proteomics data differ in the HPNE, HPNE EtOH, and HPNE-KRAS conditions, as depicted by an increased separation of data type points for the similar conditions (Figure 5A). Interestingly, the HPNE-KRAS EtOH RNA Seq and proteomics data exhibited a high level of similarity (Figure 5A), suggesting an increased correlation between transcriptional and translational processes in these cells.

Using the HPNE-KRAS EtOH data sets, we identified 44 transcripts and proteins consistently up- (*n* = 33) or down-regulated (*n* = 11) in both the RNA-Seq and proteomics data sets (Figure 5B,C). Genes/proteins with known roles in proliferative processes, including PRP19, CD81, ZPR1, RUVB1, RHOG, and NT5E were selected to validate the RNA Seq and proteomics data by both q-RT-PCR and western blot analysis (Figure 5D–I). Protein and mRNA levels of CD81 (Figure 5D), RHOG (Figure 5E), ZPR1 (Figure 5F), RUVBL1 (Figure 5G), and PRPF19 (Figure 5H) were increased in response to EtOH conditioning in HPNE-KRAS cells, whereas NT5E (Figure 5I) mRNA was decreased and protein levels were only marginally reduced in these cells. These results are mostly consistent with both the proteomics and RNA-Seq data (Figure 5C indicated by asterisks preceding gene/protein names). 

### 3.6. Differential Co-Expression Analysis Identifies SERPINE1 as a Highly Correlated Protein Unique to EtOH conditioned HPNE-KRAS Cells

**Figure 6:**
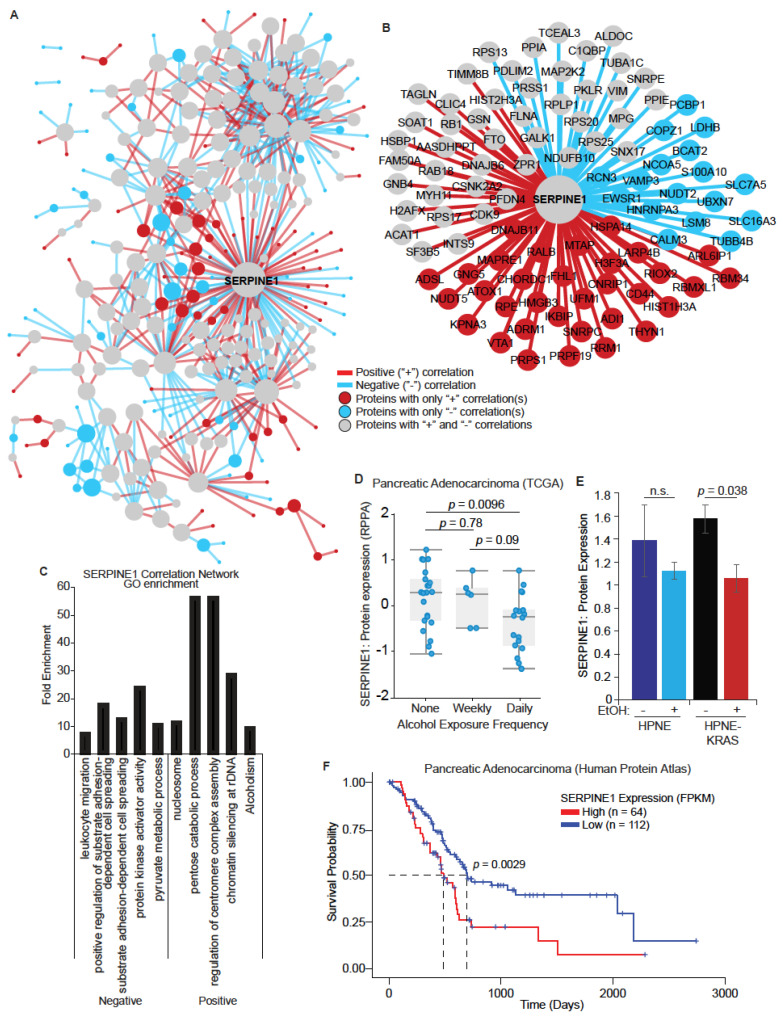
Co-expression network analysis identifies SERPINE1 as a highly interconnected protein involved in alcohol-treated and mutant KRAS HPNE cells. (**A**) Pearson correlation values for each protein–protein pair were determined for HPNE, HPNE EtOH, HPNE-KRAS, and HPNE-KRAS EtOH proteomics expression. Correlations that were ≥ +0.75 in the HPNE-KRAS EtOH and ≤−0.75 in the HPNE, HPNE EtOH, and HPNE-KRAS were considered positive correlations. Correlations that were ≤−0.75 in the HPNE-KRAS EtOH and ≥ +0.75 in the HPNE, HPNE EtOH, and HPNE-KRAS were considered negative correlations. Edges represent the presence of a positive (red) or negative (blue) correlation between connected proteins. Nodes represent proteins that have positive correlations (red), negative correlations (blue), or positive and negative correlations (grey) (*n* = 3). (**B**) Positive and negative correlations found for SERPINE1 from the overview network (**A**). (**C**) Proteins with positive or negative correlations with SERPINE1 were selected for GO term and KEGG pathway enrichment analysis (analyzed separately). The top four non-redundant terms based on Fisher’s Exact Score for both positively and negatively correlated proteins are represented with the selected term and fold enrichment. (**D**) TCGA protein expression for SERPINE1 in patients with various alcohol exposure. (**E**) SERPINE1 protein expression determined by quantitative proteomics. n.s, not significant. (**F**) Kaplan–Meier survival probability for patients with high (red) or low (blue) SERPINE1 expression.

While differential expression analysis can identify changes in protein abundance across conditions, there may be more subtle coordinated changes in sets of proteins that go unnoticed. To identify potentially important regulatory relationships between proteins, we performed a differential co-expression analysis, focusing on the EtOH-conditioned HPNE-KRAS condition. We determined what proteins were co-expressed and how co-expression changed in the context of mutant KRAS expression and EtOH treatment. In total, 287 proteins with 561 positive or negative correlations were identified (Figure 6A, annotated in Figure S4). In many instances, protein–protein pairs having positive correlations were found in HPNE-KRAS EtOH cells, but these same pairs were found negatively correlated or uncorrelated in the HPNE, HPNE EtOH, and HPNE-KRAS cells. Similarly, protein–protein pairs exhibiting negative correlations in HPNE-KRAS EtOH cells could be found positively correlated or uncorrelated in the HPNE, HPNE EtOH, and HPNE-KRAS cells.

There were several highly connected proteins that were identified in the combined network with both the negative and positive correlations, including plasminogen activator inhibitor 1 (PAI1, aka SERPINE1) (Figure 6B,C). SERPINE1 is a serine protease inhibitor overexpressed by most human cancer cell lines and is thought to stimulate growth through cell cycle progression and indirectly through activation of protease activation receptors (PAR) [38]. The top five GO/KEGG pathway terms based on the Fisher’s Exact Score for the proteins with either positive or negative correlations with SERPINE1 were identified (Figure 6C). One of the top terms associated with the SERPINE1 positive correlation network proteins was “alcoholism”. Another top term in the SERPINE1 negative correlation network was “pyruvate metabolic process” (Figure 6C). Ethanol breakdown and pyruvate both produce Acetyl Co-A, and pyruvate is decreased with ethanol exposure [39]. The differential co-expression network of SERPINE1 indicates this protein could serve as a “hub” protein in the regulation of pancreatic cell response to EtOH exposure. Indeed, TCGA data from PDAC patients annotated for alcohol intake frequency indicates that SERPINE1 protein expression is negatively associated with increased frequency of alcohol use (Figure 6D). Similarly, our proteomics data showed a significant decrease in SERPINE1 expression in the HPNE-KRAS EtOH cells compared to the non-treated HPNE-KRAS controls (Figure 6E). Furthermore, a low expression of SERPINE1 is associated with a significant survival benefit in PDAC patients (Figure 6F). Combined, these results suggest that SERPINE1 is a highly interconnected node in the network of proteins regulated by alcohol exposure in HPNE-KRAS cells, which may have implications on patient survival.

### 3.7. PDAC Subtypes Are Altered with Ethanol Treatment

**Figure 7:**
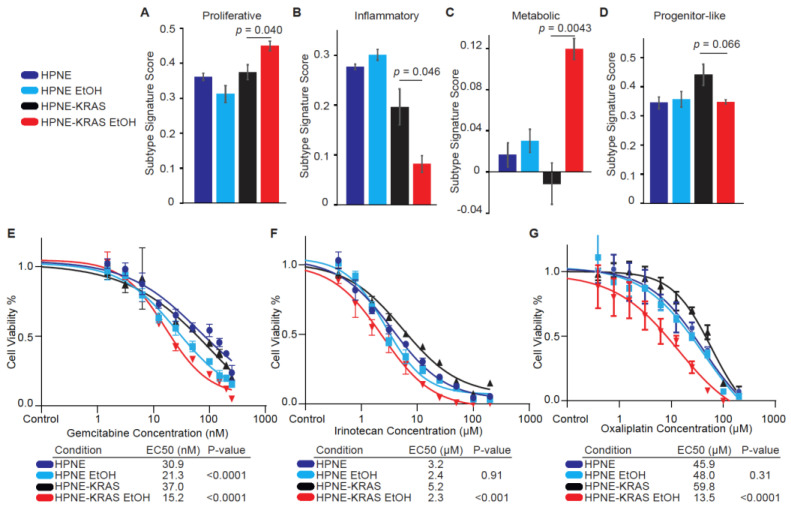
PDAC subtype signatures and chemotherapy sensitivity are associated with ethanol conditioning in mutant KRAS HPNE cells. (**A**–**D**) Proteomics expression data were mapped to PDAC subtype proteomics signatures. The top 60 variable proteins were selected and mapped to the PLS-DA model from Law et al. ROC curves were used to determine the cutoff scores for the Proliferative (**A**), Inflammatory (**B**), Metabolic (**C**), and Progenitor-like (**D**) subtypes. (**E**–**G**) Cells were treated with chemotherapeutic drugs commonly used in PDAC therapy. HPNE, HPNE EtOH, and HPNE-KRAS cells were plated at a density of 5000 cells per well, HPNE-KRAS EtOH were plated at a density of 1000 cells per well. Cells were treated with increasing doses of Gemcitabine (**E**), Irinotecan (**F**), or Oxaliplatin (**G**) for 3 days. Cell viability was assessed by CellTiter-Glo following drug exposure to assess cell viability. Relative luminescence was read on a plate reader and IC50 values were determined by GraphPad Prism. Mean ± SEM; *n* = 4.

Our previous proteomic observations in metastatic PDAC tissues indicated that alcohol use in these patients was associated with a decrease in the Inflammatory subtype and an increase in the Proliferative and Metabolic subtypes [6]. To determine the impacts of EtOH on subtype signatures in the presence and absence of mutant KRAS in the HPNE cell lines, we evaluated the PDAC subtype signatures for each of the HPNE and HPNE-KRAS cells lines with or without EtOH conditioning (Figure 7A–D). EtOH did not significantly impact the subtype signatures in the HPNE cells. However, the HPNE-KRAS EtOH cells exhibited significant increases in the Proliferative (Figure 7A) and Metabolic (Figure 7C) subtype signatures and a significant decrease in the Inflammatory subtype signature (Figure 7B). Notably, the change in subtype signatures in response to EtOH conditioning in HPNE-KRAS cells was most evident in the suppression of the Inflammatory signature and an increase in the Metabolic signature (Figure 7B,C). These trends are largely consistent with what we previously observed in clinical PDAC samples [6], suggesting that the impact of EtOH on PDAC subtype development could be dependent upon the mutational status of KRAS.

Furthermore, our previous study determined that clinical response to FOLFIRINOX is also associated with PDAC subtypes [3,6]. Because a shift in subtype signatures in response to EtOH conditioning could also influence response to therapeutic agents, we analyzed the impact of three commonly used pancreatic cancer drugs (gemcitabine, irinotecan, and oxaliplatin) on cell viability (Figure 7E–G). EtOH conditioning of HPNE cells resulted in a lower EC50 in the gemcitabine treatment compared to control HPNE cells, but no impact on EC50s was observed for the irinotecan or oxaliplatin treatments in this cell line. However, the EC50s for gemcitabine (Figure 7E), irinotecan (Figure 7F), and oxaliplatin (Figure 7G) were significantly lower in HPNE-KRAS EtOH cells in comparison to the control HPNE-KRAS cells. These results are consistent with expectations that treatment responses would improve as cells acquire the Metabolic signature and lose the Inflammatory signature.

## 4. Discussion

Epidemiological studies show a consistent association between heavy alcohol use and risk of PDAC development [12,13,14,15]. Additionally, recent studies have shown that ethanol exposure increases PanIN and PDAC development in mutant KRAS murine models [18,19]. The results suggest that alcohol consumption in the context of an underlying genetic susceptibility, such as KRAS mutation, potentiates PDAC tumorigenesis. In this study, we observed that ethanol conditioning increased proliferation, a hallmark of cancer, in HPNE cells harboring the G12D KRAS mutation, but not in HPNE with wild-type KRAS. Delineating the molecular signatures associated with alcohol exposure in the context of mutant KRAS is essential to understanding the effects of alcohol on the etiology of PDAC. In this study, we utilized a multi-omics approach to identify gene and protein expression signatures unique to alcohol conditioning in mutant KRAS-expressing HPNE cells. 

Individually, both KRAS mutation and alcohol exposure alter the molecular signatures of pancreas tissues. KRAS mutation and gene dosage drives tumorigenesis and metastasis associated with differential gene expression signatures [40]. Patients with an alcoholic etiology of pancreatitis displayed differential gene expression signatures compared to hereditary etiologies of pancreatitis [41]. In our study, the individual variables of either alcohol exposure or KRAS mutation status were associated with changes in the transcriptome and proteome. Furthermore, a previous analysis using a breast cancer cell line found that ethanol-induced activation of the Ras/MEK/MAPK signaling promotes cell growth, suggesting pathway-specific requirements for alcohol-induced proliferation [42]. Indeed, our study has now identified a unique set of genes and proteins that are differentially expressed and dependent upon both alcohol exposure and KRAS mutation status in HPNE cells. Functional analysis of our RNA-seq datasets comparing HPNE-KRAS with and without ethanol conditioning identified an enrichment of GO terms for protein translation of differentially upregulated proteins, while downregulated proteins were associated with terms for actin and cytoskeleton arrangement. The downregulation of proteins involved in actin and cytoskeletal organization could be indicative of a migratory phenotype, which has been observed in breast cancer cells treated with ethanol [16,43]. 

In addition, we observed a differential response to combined ethanol conditioning and KRAS mutation status in the proteomics data. In the ethanol-conditioned HPNE-KRAS cells, 95 and 37 proteins were differentially up- or down-regulated, respectively, in comparison with HPNE-KRAS controls. In agreement with the RNA-Seq data, the up-regulated proteins were enriched for GO terms associated with transcription and translational processes, with RNA/poly(A) binding being the top term. The GO terms associated with the downregulated terms related to cell–cell adhesion and contact, which may be related to the downregulation and disruption of the actin and cytoskeleton related genes observed in the RNA-Seq results. Notably, ethanol-induced disruption of cell–cell contact has previously been demonstrated in the brain, colon, and pancreas acinar cells [44,45,46,47,48]. These results from the transcriptomic and proteomic analyses suggest alcohol exposure combined with expression of mutant KRAS affects a spectrum of cellular functions that could contribute to the development of tumor phenotypes.

MCIA of the RNA-Seq and Proteomics data sets allows for the evaluation of the differences between gene and protein expression in different treatment and genetic contexts. The HPNE, HPNE EtOH, and HPNE-KRAS cells display variation between their transcriptomic and proteomic profiles. This is likely attributable to the multiple levels of regulation controlling transcriptional, posttranscriptional, translational, and posttranslational processes that can result in poor correlation between transcript and protein levels. Interestingly, the ethanol conditioning of HPNE-KRAS appears to promote an increased correlation between the transcriptomic and proteomic profiles, which could be attributable to an increase in translational processes suggested by the GO term enrichments. Quantitative increases in protein translation support cell growth and proliferation, while qualitative differences in translation, such as preferential expression of oncogenes, likely contribute to cancer development [49,50]. Increased correlation between mRNA transcripts and translated proteins may indicate the ethanol-conditioned HPNE-KRAS cells are better able to respond to signaling cues affecting transcriptional/translational programs. 

The increase in proliferation of the HPNE-KRAS EtOH cells could be, in part, due to the impact of metabolic byproducts of ethanol causing DNA mutations or protein adducts that change the proliferation rate of these cells due to the dysregulation of cell cycle-related proteins. However, we did not observe significant changes in the acetaldehyde or ROS production in the HPNE-KRAS EtOH cells. Acetaldehyde appeared to be metabolized at the same rate regardless of KRAS status and non-treated versus chronic ethanol- treated conditions. There is a possibility that additional ethanol breakdown pathways and ethanol usage pathways could be involved in the reduction of chronic ethanol treated cells, however the major byproducts of ethanol metabolism do not show significant alterations dependent upon either KRAS or alcohol conditioning. Nevertheless, additional carcinogenic byproducts, for example lipid peroxides malondialdehyde (MDA) and 4-hydroxyneoneal (4-HNE), could play a role on carcinogenesis and producing a highly proliferative phenotype [36,51,52]. Alternatively, the changes in the cell cycle in the HPNE-KRAS EtOH cells indicate the potential dysregulation of the pathways regulating this process. 

In a network-based analysis, we generated correlation networks for protein expression in the different alcohol and KRAS mutation conditions. We aimed to determine what proteins were coordinately expressed in the HPNE cells and investigated how ethanol and KRAS mutation affects these correlations. We found that SERPINE1 had positive or negative correlations with 95 proteins, and was the most highly connected protein in the differential co-expression network. The correlations identified are either absent by not meeting the Pearson correlation threshold, or oppositely correlated, making these unique to the HPNE-KRAS EtOH-treated cells. “Alcoholism” was the top term found for the upregulated correlations with SERPINE1, suggesting the potential value of this analysis relevant to the dataset. SERPINE1 is pro-tumorigenic in many types of cancers, promoting growth, metastasis, and invasiveness of tumor cells, including PDAC [53]. It has been used as a mesenchymal marker [54,55], implicated in EMT, metastasis, and angiogenesis [56,57], fibrosis and immunosuppression [58], and increased expression is an indication of poor survival [59]. The independent validation that SERPINE1 is down-regulated in clinical PDAC samples annotated for frequent alcohol use further supports a potential role of this protein in alcohol-associated PDAC development. However, further experiments are required to understand the mechanistic role of this protein in alcohol associated PDAC.

The HPNE cell culture model used in this study reflects the associations observed in PDAC patients who used alcohol [6], and KRAS mutation was necessary to observe the ethanol-induced changes in PDAC subtype signatures in the HPNE cells. These changes in subtype signatures are likely reflective of the altered gene and protein expression caused by ethanol conditioning in KRAS mutant HPNE cells. It is likely that gemcitabine, irinotecan, and oxaliplatin demonstrated lower EC50s in mutant KRAS-expressing HPNE cells conditioned with ethanol because PDAC subtypes are predictive of response to chemotherapeutic agents [3,6]. However, it is also possible that the link between chemotherapeutic efficacy in the HPNE-KRAS EtOH cells could be attributed to either changes in the expression of specific genes/proteins or to a general trait, such as a subtype with increased proliferation. Since each drug tested in our study has a different mechanism of action [60,61,62], this argues that the changes in chemosensitivity is not likely arising from alterations in a single protein, but rather a general trait of the cell population. Based on the changes to subtype signatures, our results suggest that alcohol conditioning can promote either clonal outgrowth or subtype plasticity through selective pressure. While this study observed improved chemosensitivity following EtOH conditioning, the negative impacts of alcohol itself and the difficulties envisioned with sustaining such a high exposure level over many months to effect a change in subtype signature likely preclude its clinical application. Rather, targeted strategies for modulating signaling pathways and networks associated with subtype delineation, plasticity, or selection should be explored more broadly to determine their potential clinical benefits in terms of preventing or overcoming chemoresistance. 

## 5. Conclusions

This study has determined that alcohol preferentially induces and increases in the proliferative potential of pancreas HPNE cell that express mutant KRAS. This study indicates that the influence of alcohol on pancreas cells is dependent upon the KRAS mutation status of the cell. Combined, alcohol and mutant KRAS potentiate a proliferative phenotype and influence subtype delineation associated with alterations in the transcriptome and proteome.

## Data Availability

All data generated and analyzed in this study are included in the article or can be obtained from the authors upon reasonable request. Please direct all requests to N.T.W. (nicholas.woods@unmc.edu). The mass spectrometry proteomics data have been deposited to the ProteomeXchange Consortium via the PRIDE [63] partner repository with the dataset identifier PXD030180. RNA-Sequencing data have been submitted to NCBI under the BioProject accession PRJNA784536.

## Supplementary Materials

The following supporting information can be downloaded at: https://www.mdpi.com/article/10.3390/cancers14081968/s1, Table S1. RNA-Sequencing gene counts normalized by DESeq2, Table S2. Differentially Expressed Genes in the HPNE-KRAS EtOH condition compared to HPNE-KRAS, Log2 Fold Change ±2, Table S3. 1635 upregulated genes in HPNE-KRAS EtOH (compared to HPNE-KRAS) that were identified only in HPNE-KRAS EtOH and absent in HPNE-EtOH vs. HPNE. See Venn diagram in Figure 3C, Table S4. 1388 downregulated genes in HPNE-KRAS EtOH (compared to HPNE-KRAS) identified only in HPNE-KRAS EtOH and absent in HPNE-EtOH vs. HPNE. See Venn diagram in Figure 3C, Table S5. Gene ontology enrichment analysis of gene transcripts upregulated in HPNE EtOH treated cells vs HPNE untreated control, Table S6. Gene ontology enrichment analysis of gene transcripts downregulated in HPNE EtOH treated cells vs. HPNE untreated control, Table S7. Gene ontology enrichment analysis of gene transcripts upregulated in HPNE-KRAS EtOH treated cells vs HPNE-KRAS untreated control, Table S8. Gene ontology enrichment analysis of gene transcripts downregulated in HPNE-KRAS EtOH treated cells vs. HPNE-KRAS untreated control, Table S9. Proteomics expression values of 3 replicates in HPNE and ethanol treated HPNE cells including the uncorrected *p*-value (student’s *t*-test), FDR, and log2 fold change, Table S10. Proteomics expression values of 3 replicates in HPNE-KRAS and ethanol-treated HPNE-KRAS cells, including the uncorrected *p*-value (student’s *t*-test), FDR, and log2 fold change, Table S11. Gene ontology enrichment analysis of proteins upregulated in HPNE EtOH-treated cells vs HPNE untreated control, Table S12. Gene ontology enrichment analysis of proteins downregulated in HPNE EtOH-treated cells vs. HPNE untreated control, Table S13. Gene ontology enrichment analysis of proteins upregulated in HPNE-KRAS EtOH-treated cells vs. HPNE-KRAS untreated control, Table S14. Gene ontology enrichment analysis of proteins downregulated in HPNE-KRAS EtOH-treated cells vs HPNE-KRAS untreated control; Figure S1. The gating strategies for the EtOH-treated and untreated cell line cell cycle analysis, Figure S2. The gating strategies for the EtOH-treated and untreated cell line intra-cellular Reactive Oxygen Species (ROS) measurements, Figure S3. (A) Differentially expressed transcripts quantified by RNA Sequencing with a fold change of ±2.0 from the untreated control cell lines. (*n* = 1). (B) The biotypes of all transcripts detected through RNA Seq. Biotypes of each transcript determined by Ensembl. Biotype categories that did not fall into protein coding, non-coding RNA, or pseudogenes were categorized as “other”, Figure S4. Differential Co-Expression Network. Pearson correlation values for each protein–protein pair were determined for HPNE, HPNE EtOH, HPNE-KRAS, and HPNE-KRAS EtOH proteomics expression. Correlations that were ≥ +0.75 in the HPNE-KRAS EtOH and ≤−0.75 in the HPNE, HPNE EtOH, and HPNE-KRAS were considered positive correlations. Correlations that were ≤−0.75 in the HPNE-KRAS EtOH and ≥ +0.75 in the HPNE, HPNE EtOH, and HPNE-KRAS were considered negative correlations. Edges represent the presence of a positive (red) or negative (blue) correlation between connected proteins. Nodes represent proteins that have positive correlations (red), negative correlations (blue), or positive and negative correlations (grey) (*n* = 3). Node size corresponds to the number of correlations associated with each protein. Figure S5. The original blots of Figure 5.
