# Supplementary material for: Combined Alcohol Exposure and KRAS Mutation in Human Pancreatic Ductal Epithelial Cells Induces Proliferation and Alters Subtype Signatures Determined by Multi-Omics Analysis
Source: Cancers (Basel). 2022 Apr 13;14(8):1968. doi: 10.3390/cancers14081968 (PMC9027648; doi:10.3390/cancers14081968)
Supplement: Supplementary file 1 [file cancers-14-01968-s001.zip › supplementary/Figure_S1.pdf]

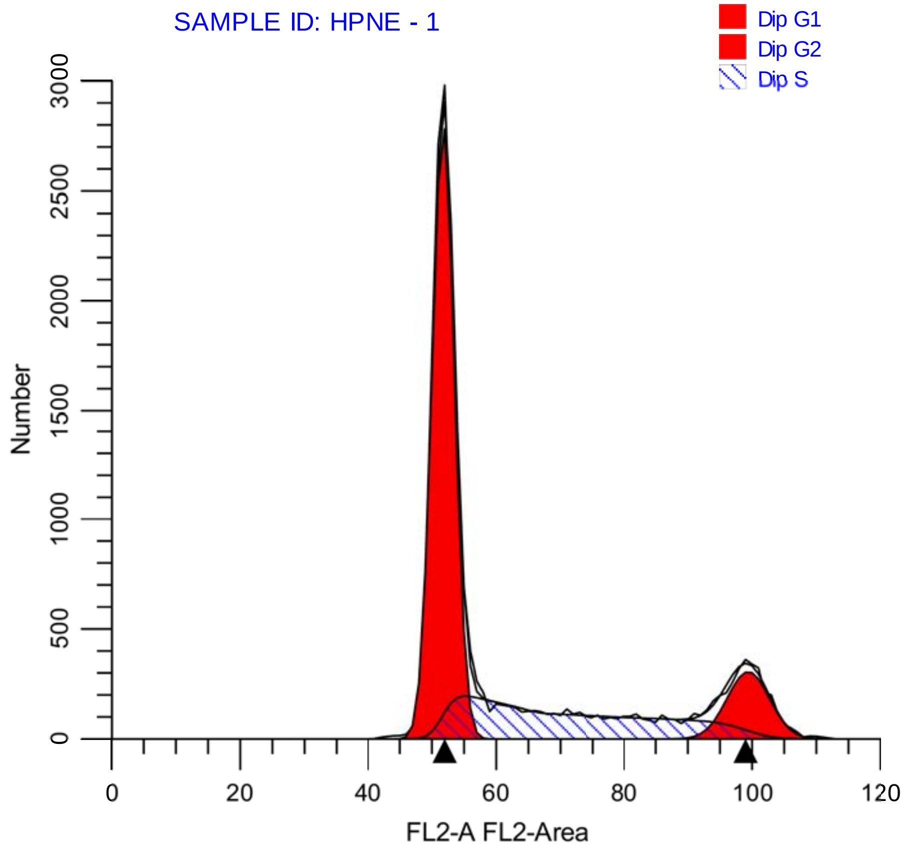

File analyzed: EC13J20.001
Date analyzed: 13-Jan-2020
Model: 1nn0n_DSD
Analysis type: Manual analysis
Auto Linearity: No

Ploidy Mode: First cycle is diploid

Diploid: 100.00 %
Dip G1: 59.65 % at 51.78
Dip G2: 12.41 % at 99.42
Dip S: 27.94 % G2/G1: 1.92
%CV: 3.29

Total S-Phase: 27.94 %
Total B.A.D.: 0.00 % no debris no aggs

Debris: %
Aggregates: %
Modeled events: 20329
All cycle events: 20329
Cycle events per channel: 418
RCS: 1.767

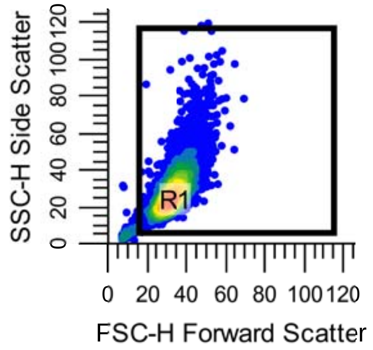

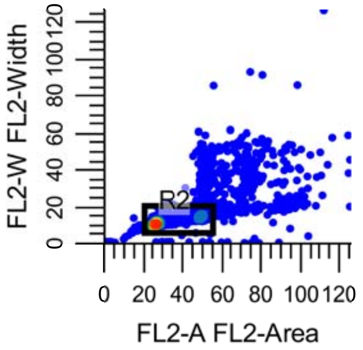

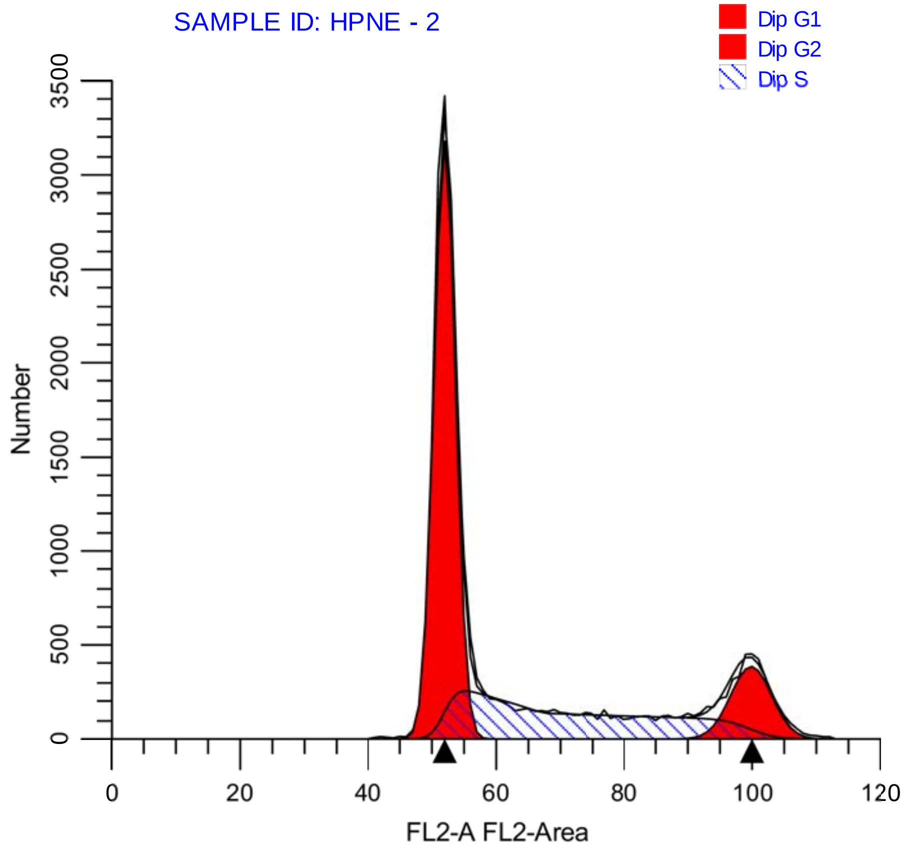

File analyzed: EC13J20.002
Date analyzed: 13-Jan-2020
Model: 1nn0n_DSD
Analysis type: Manual analysis
Auto Linearity: No

Ploidy Mode: First cycle is diploid

Diploid: 100.00 %
Dip G1: 56.42 % at 52.02
Dip G2: 12.99 % at 99.87
Dip S: 30.59 %   G2/G1: 1.92
%CV: 3.16

Total S-Phase: 30.59 %
Total B.A.D.: 0.00 %   no debris no aggs

Debris: %
Aggregates: %
Modeled events: 23574
All cycle events: 23574
Cycle events per channel: 483
RCS: 2.830

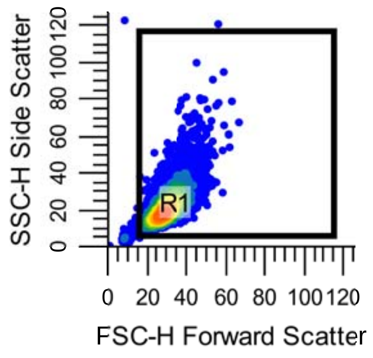

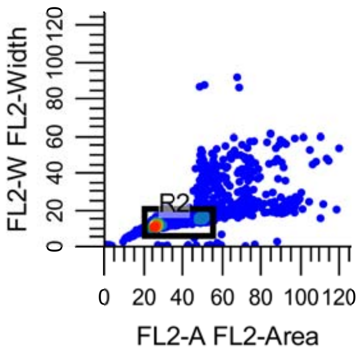

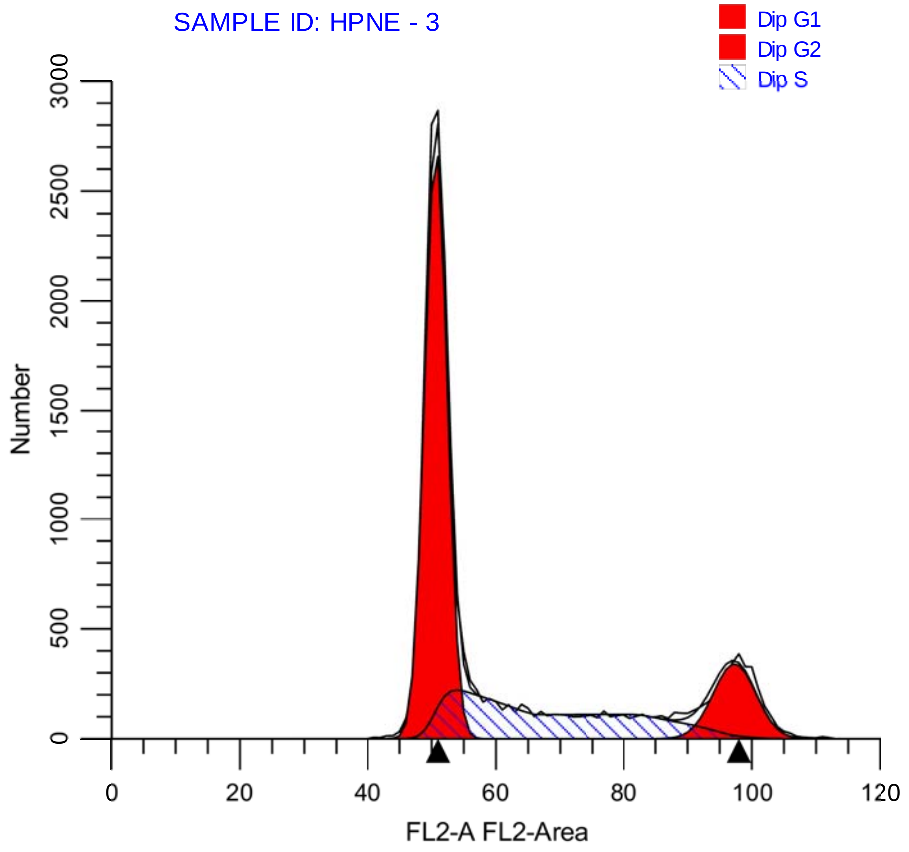

File analyzed: EC13J20.003
Date analyzed: 13-Jan-2020
Model: 1nn0n_DSD
Analysis type: Manual analysis
Auto Linearity: No

Ploidy Mode: First cycle is diploid

Diploid: 100.00 %
Dip G1: 57.98 % at 50.69
Dip G2: 13.99 % at 97.32
Dip S: 28.03 % G2/G1: 1.92
%CV: 3.38

Total S-Phase: 28.03 %
Total B.A.D.: 0.00 % no debris no aggs

Debris: %
Aggregates: %
Modeled events: 20257
All cycle events: 20257
Cycle events per channel: 425
RCS: 2.832

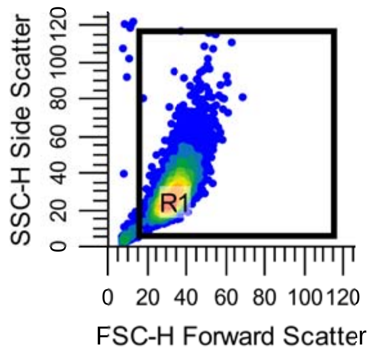

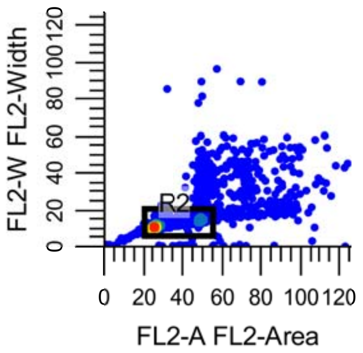

ModFit LT V4.0.5(Win)

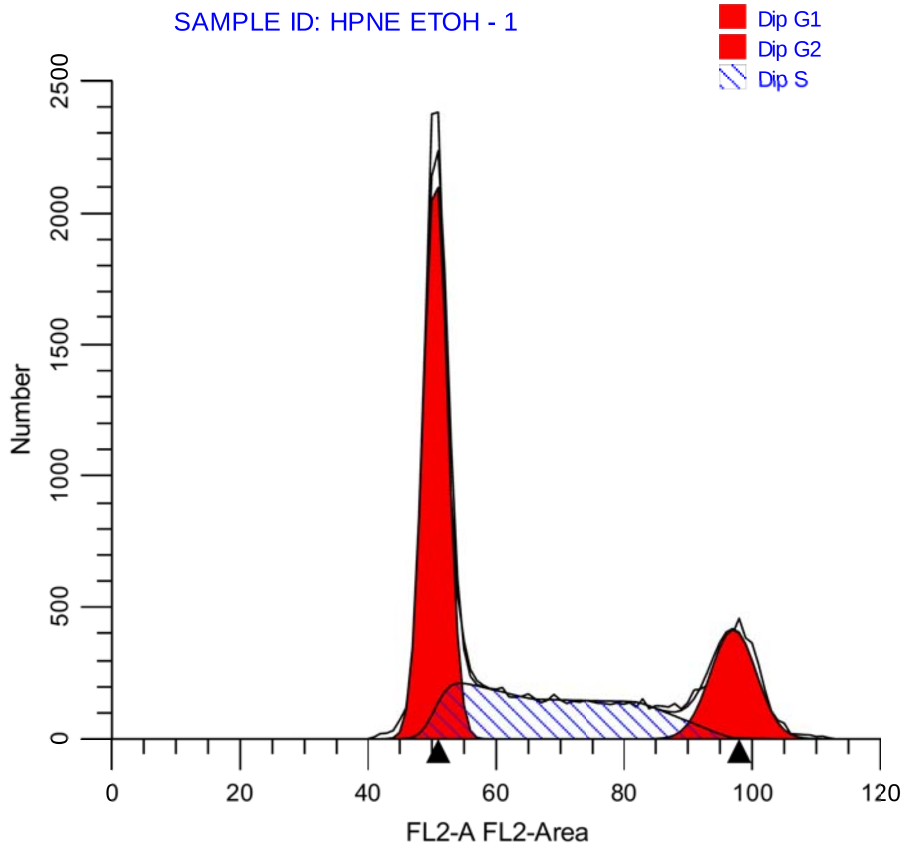

File analyzed: EC13J20.004
Date analyzed: 13-Jan-2020
Model: 1nn0n_DSD
Analysis type: Manual analysis
Auto Linearity: No

Ploidy Mode: First cycle is diploid

Diploid: 100.00 %
Dip G1: 49.85 % at 50.58
Dip G2: 18.31 % at 97.11
Dip S: 31.84 % G2/G1: 1.92
%CV: 3.65

Total S-Phase: 31.84 %
Total B.A.D.: 0.00 % no debris no aggs

Debris: %
Aggregates: %
Modeled events: 20177
All cycle events: 20177
Cycle events per channel: 424
RCS: 3.252

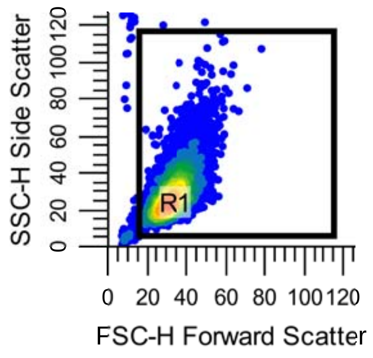

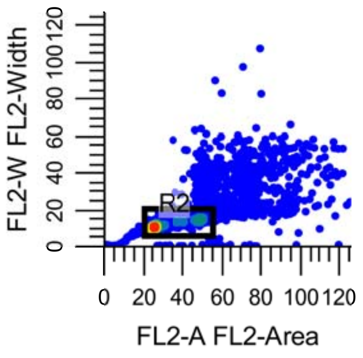

ModFit LT V4.0.5(Win)

SAMPLE ID: HPNE ETOH - 2

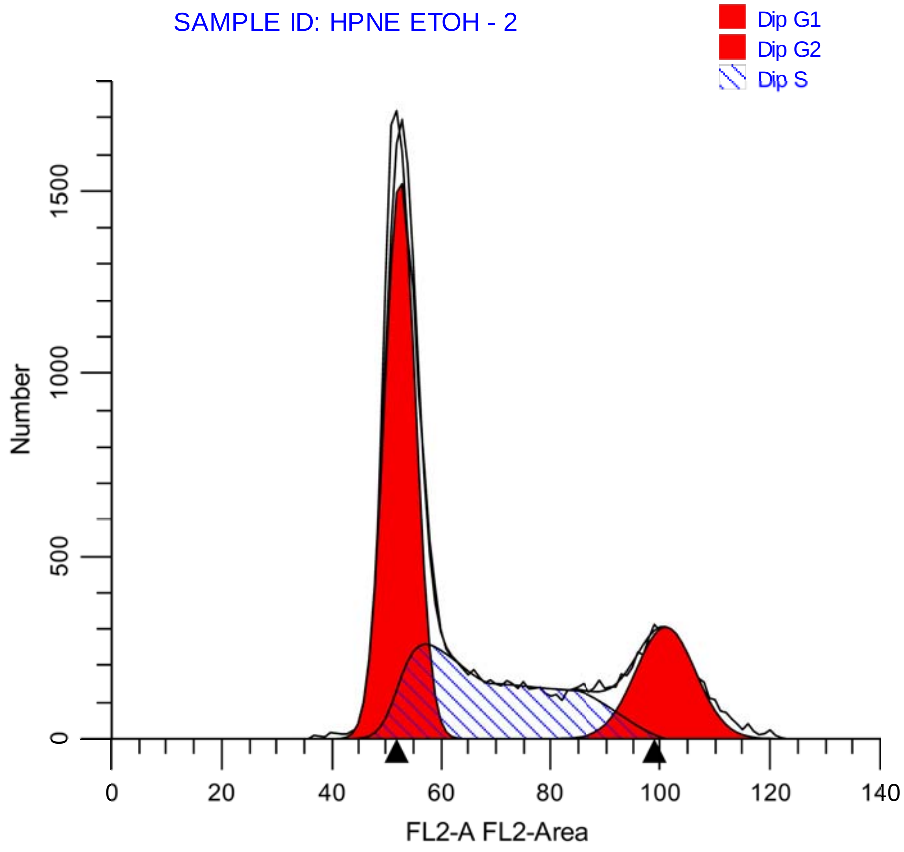

File analyzed: EC13J20.005
Date analyzed: 13-Jan-2020
Model: 1nn0n_DSD
Analysis type: Manual analysis
Auto Linearity: No

Ploidy Mode: First cycle is diploid

Diploid: 100.00 %
Dip G1: 49.00 % at 52.62
Dip G2: 18.74 % at 101.03
Dip S: 32.26 %   G2/G1: 1.92
%CV: 5.34

Total S-Phase: 32.26 %
Total B.A.D.: 0.00 %   no debris no aggs

Debris: %
Aggregates: %
Modeled events: 22127
All cycle events: 22127
Cycle events per channel: 448
RCS: 3.847

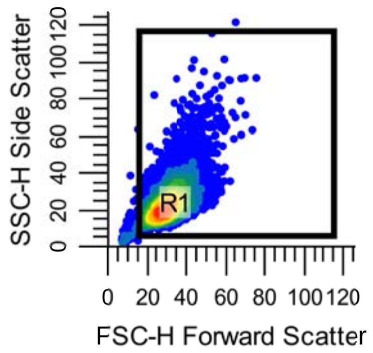

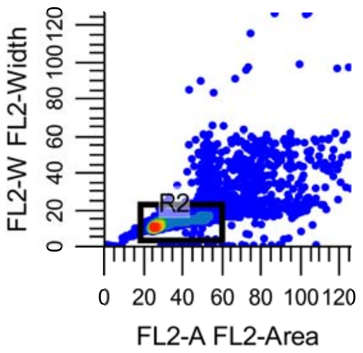

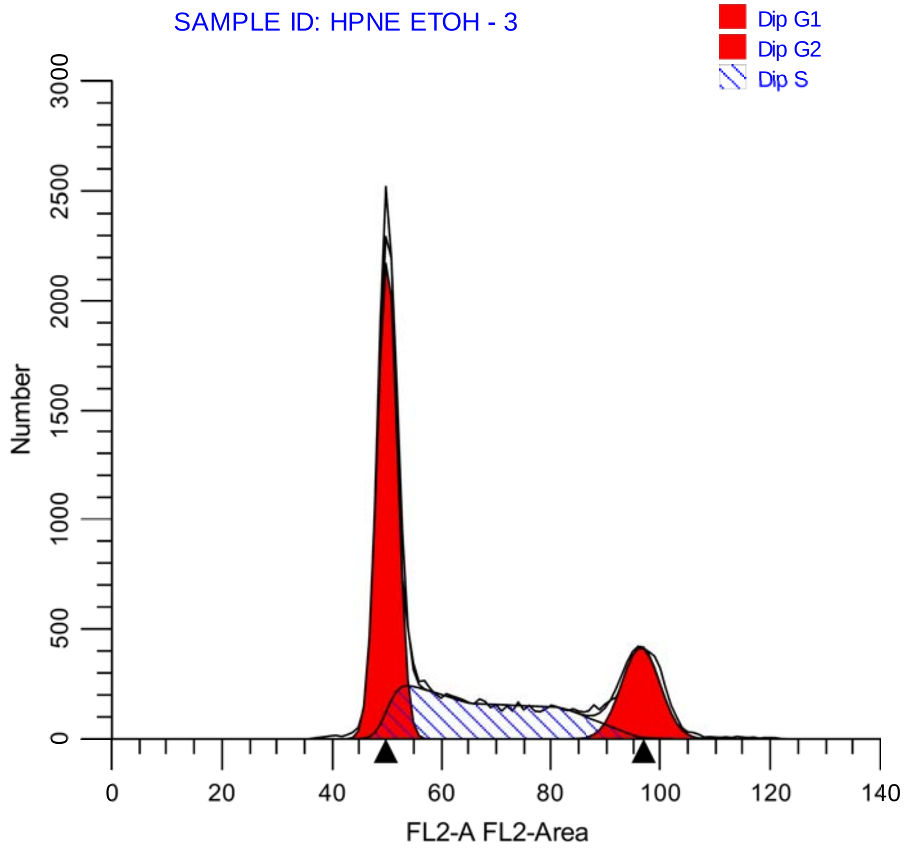

File analyzed: EC13J20.006
Date analyzed: 13-Jan-2020
Model: 1nn0n_DSD
Analysis type: Manual analysis
Auto Linearity: No

Ploidy Mode: First cycle is diploid

Diploid: 100.00 %
Dip G1: 48.73 % at 50.25
Dip G2: 17.61 % at 96.48
Dip S: 33.66 % G2/G1: 1.92
%CV: 3.59

Total S-Phase: 33.66 %
Total B.A.D.: 0.00 % no debris no aggs

Debris: %
Aggregates: %
Modeled events: 20617
All cycle events: 20617
Cycle events per channel: 437
RCS: 3.662

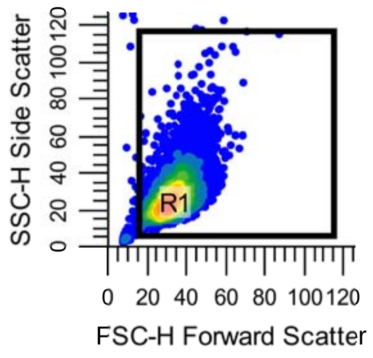

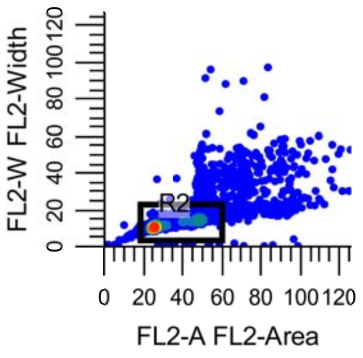

ModFit LT V4.0.5(Win)

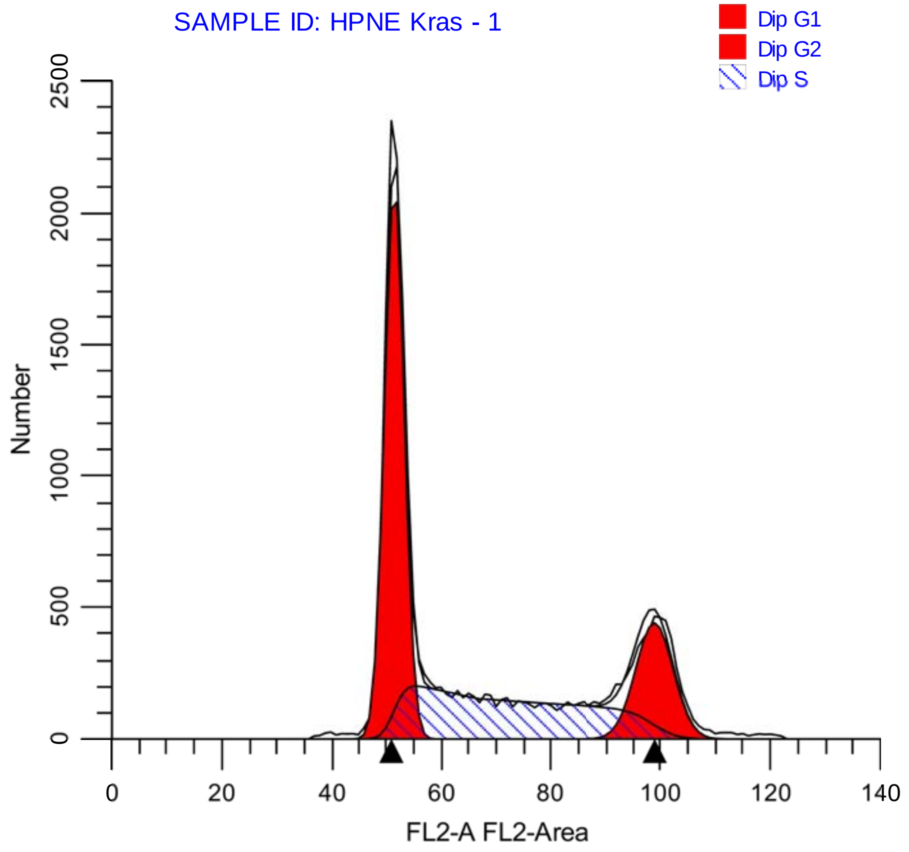

File analyzed: EC13J20.007
Date analyzed: 13-Jan-2020
Model: 1nn0n_DSD
Analysis type: Manual analysis
Auto Linearity: No

Ploidy Mode: First cycle is diploid

Diploid: 100.00 %
Dip G1: 46.65 % at 51.54
Dip G2: 18.53 % at 98.96
Dip S: 34.82 % G2/G1: 1.92
%CV: 3.40

Total S-Phase: 34.82 %
Total B.A.D.: 0.00 % no debris no aggs

Debris: %
Aggregates: %
Modeled events: 20162
All cycle events: 20162
Cycle events per channel: 416
RCS: 4.982

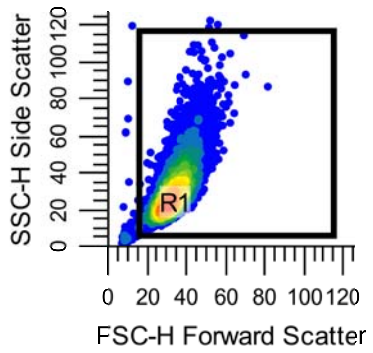

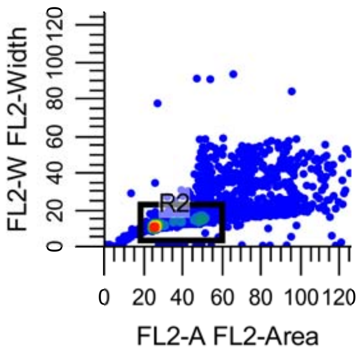

ModFit LT V4.0.5(Win)

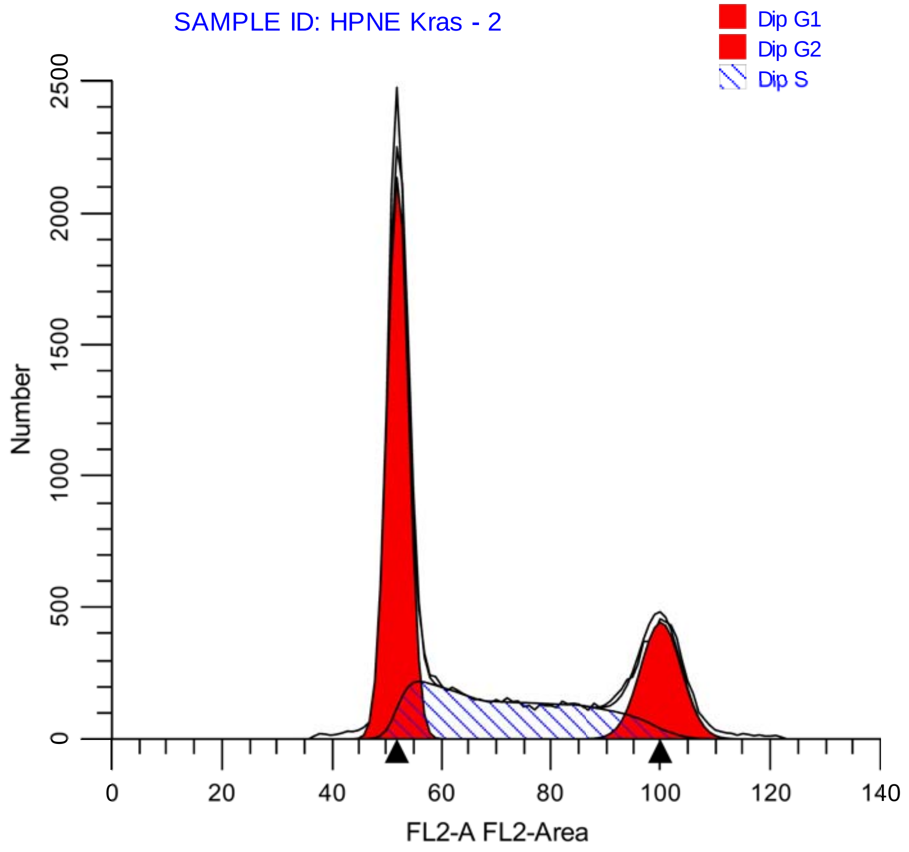

File analyzed: EC13J20.008
Date analyzed: 13-Jan-2020
Model: 1nn0n_DSD
Analysis type: Manual analysis
Auto Linearity: No

Ploidy Mode: First cycle is diploid

Diploid: 100.00 %
Dip G1: 48.43 % at 52.15
Dip G2: 19.10 % at 100.14
Dip S: 32.47 %   G2/G1: 1.92
%CV: 3.67

Total S-Phase: 32.47 %
Total B.A.D.: 0.00 %   no debris no aggs

Debris: %
Aggregates: %
Modeled events: 21457
All cycle events: 21457
Cycle events per channel: 438
RCS: 4.393

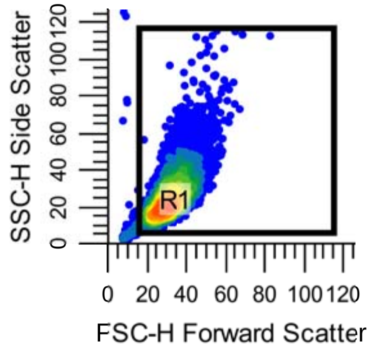

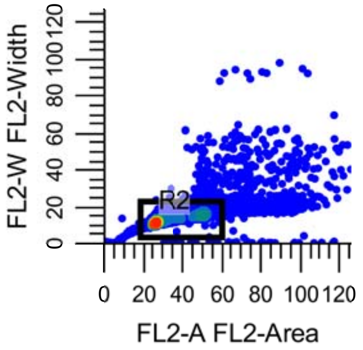

ModFit LT V4.0.5(Win)

SAMPLE ID: HPNE Kras - 3

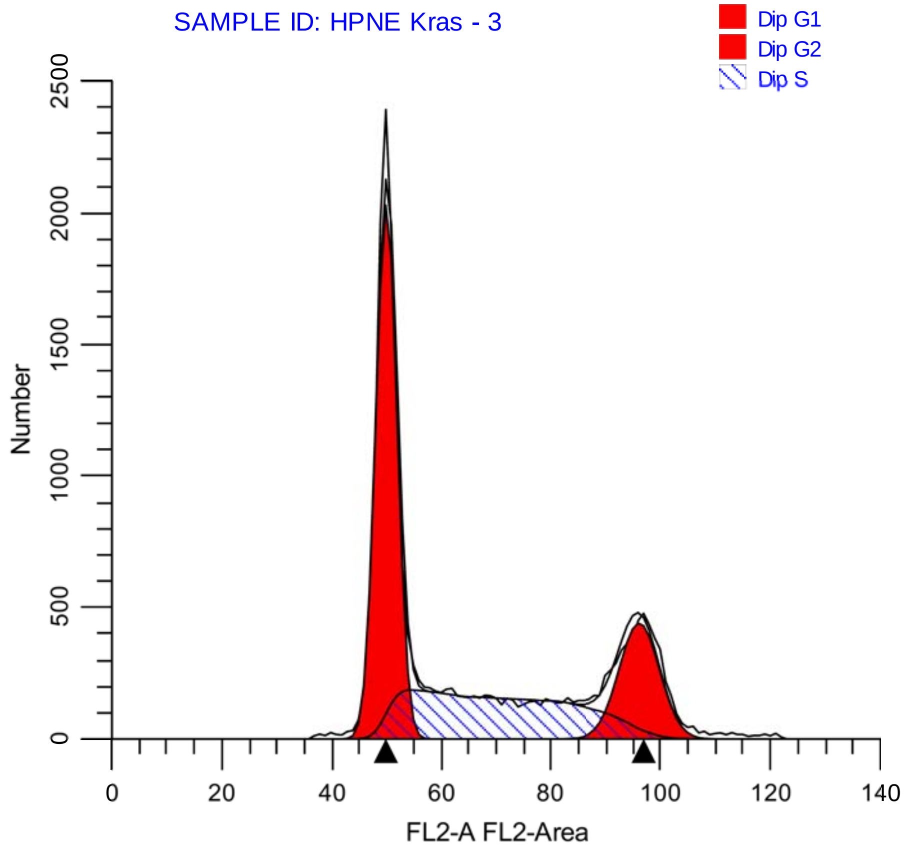

File analyzed: EC13J20.009
Date analyzed: 13-Jan-2020
Model: 1nn0n_DSD
Analysis type: Manual analysis
Auto Linearity: No

Ploidy Mode: First cycle is diploid

Diploid: 100.00 %
Dip G1: 47.42 % at 50.11
Dip G2: 19.55 % at 96.20
Dip S: 33.03 %   G2/G1: 1.92
%CV: 3.77

Total S-Phase: 33.03 %
Total B.A.D.: 0.00 %   no debris no aggs

Debris: %
Aggregates: %
Modeled events: 20533
All cycle events: 20533
Cycle events per channel: 436
RCS: 4.977

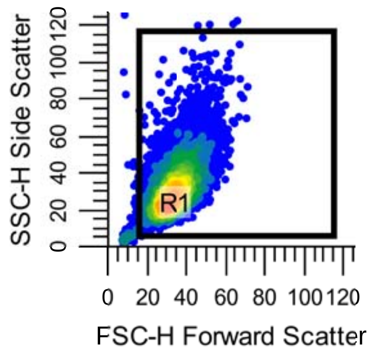

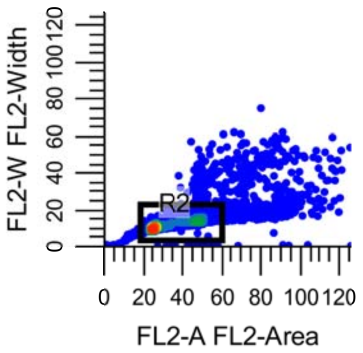

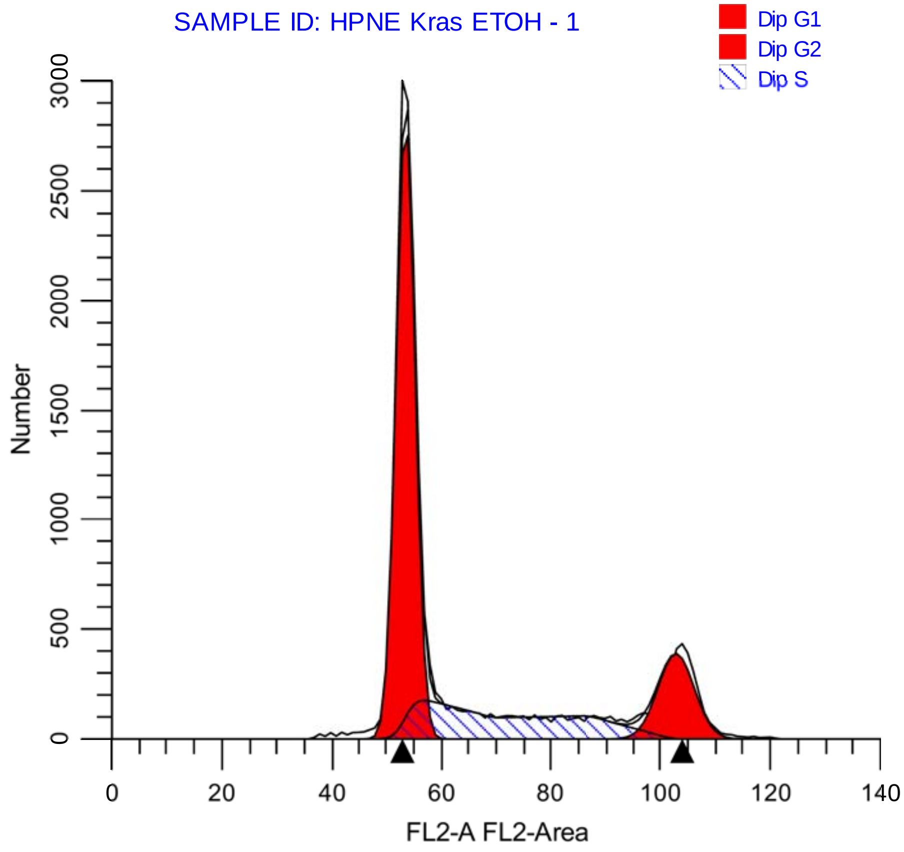

File analyzed: EC13J20.010
Date analyzed: 13-Jan-2020
Model: 1nn0n_DSD
Analysis type: Manual analysis
Auto Linearity: No

Ploidy Mode: First cycle is diploid

Diploid: 100.00 %
Dip G1: 59.87 % at 53.59
Dip G2: 15.64 % at 102.90
Dip S: 24.49 % G2/G1: 1.92
%CV: 3.15

Total S-Phase: 24.49 %
Total B.A.D.: 0.00 % no debris no aggs

Debris: %
Aggregates: %
Modeled events: 20293
All cycle events: 20293
Cycle events per channel: 403
RCS: 4.547

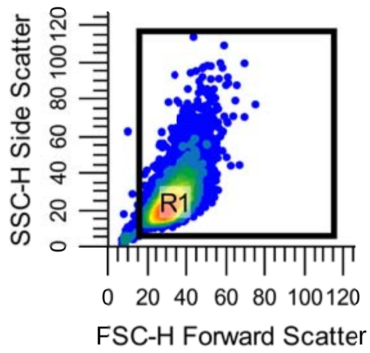

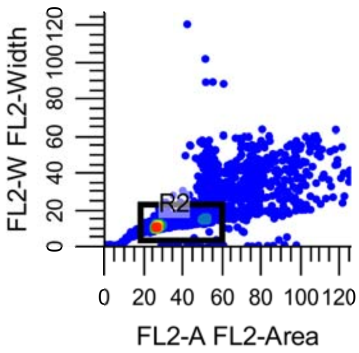

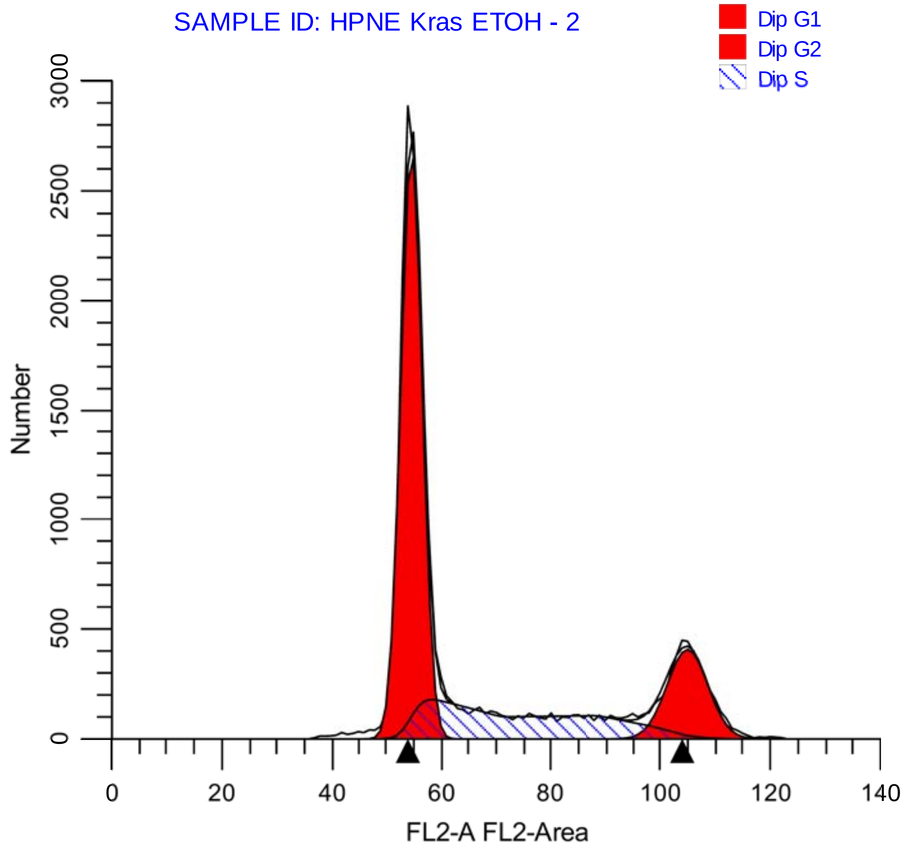

File analyzed: EC13J20.011
Date analyzed: 13-Jan-2020
Model: 1nn0n_DSD
Analysis type: Manual analysis
Auto Linearity: No

Ploidy Mode: First cycle is diploid

Diploid: 100.00 %
Dip G1: 58.55 % at 54.66
Dip G2: 16.92 % at 104.96
Dip S: 24.53 % G2/G1: 1.92
%CV: 3.49

Total S-Phase: 24.53 %
Total B.A.D.: 0.00 % no debris no aggs

Debris: %
Aggregates: %
Modeled events: 22226
All cycle events: 22226
Cycle events per channel: 433
RCS: 4.058

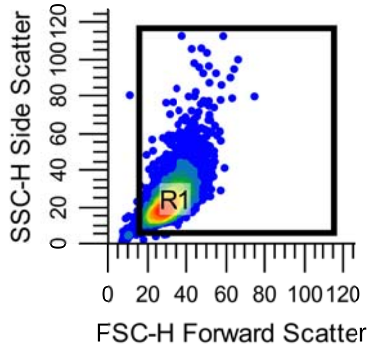

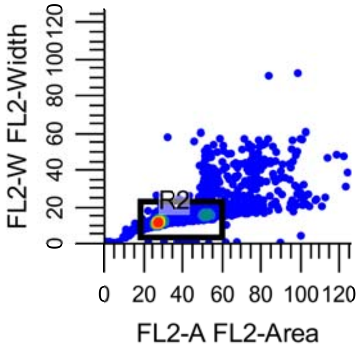

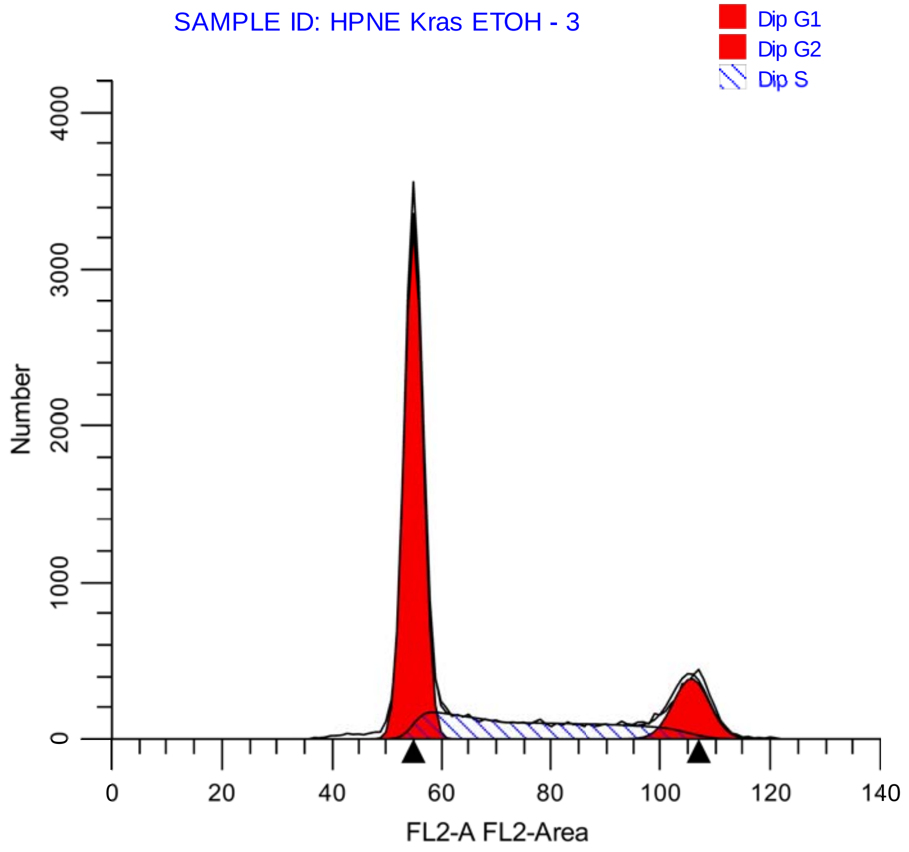

File analyzed: EC13J20.012
Date analyzed: 13-Jan-2020
Model: 1nn0n_DSD
Analysis type: Manual analysis
Auto Linearity: No

Ploidy Mode: First cycle is diploid

Diploid: 100.00 %
Dip G1: 61.71 % at 55.03
Dip G2: 13.83 % at 105.65
Dip S: 24.45 % G2/G1: 1.92
%CV: 3.06

Total S-Phase: 24.45 %
Total B.A.D.: 0.00 % no debris no aggs

Debris: %
Aggregates: %
Modeled events: 22622
All cycle events: 22622
Cycle events per channel: 438
RCS: 4.587

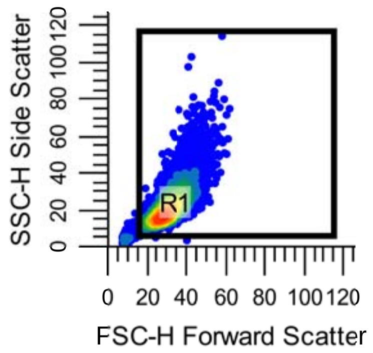

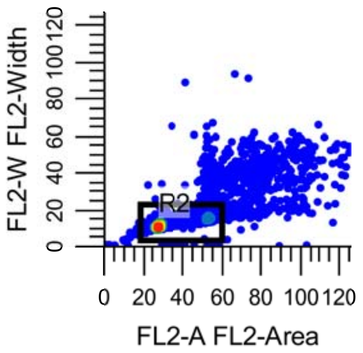

ModFit LT V4.0.5(Win)

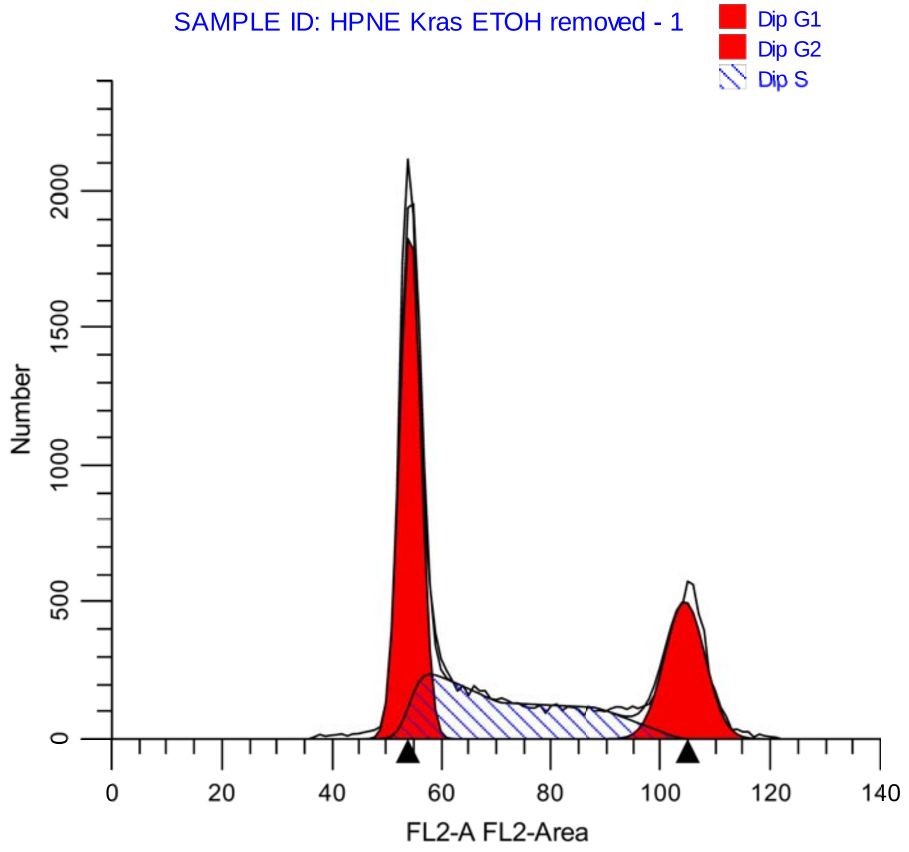

File analyzed: EC13J20.013
Date analyzed: 13-Jan-2020
Model: 1nn0n_DSD
Analysis type: Manual analysis
Auto Linearity: No

Ploidy Mode: First cycle is diploid

Diploid: 100.00 %
Dip G1: 45.03 % at 54.42
Dip G2: 22.98 % at 104.48
Dip S: 31.99 % G2/G1: 1.92
%CV: 3.49

Total S-Phase: 31.99 %
Total B.A.D.: 0.00 % no debris no aggs

Debris: %
Aggregates: %
Modeled events: 19988
All cycle events: 19988
Cycle events per channel: 391
RCS: 4.972

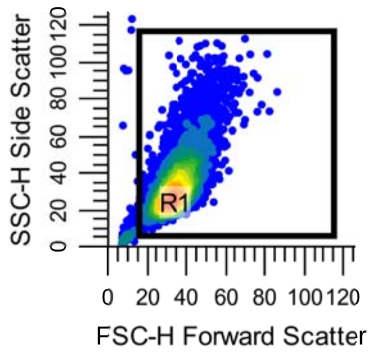

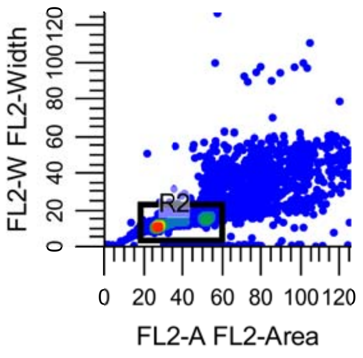

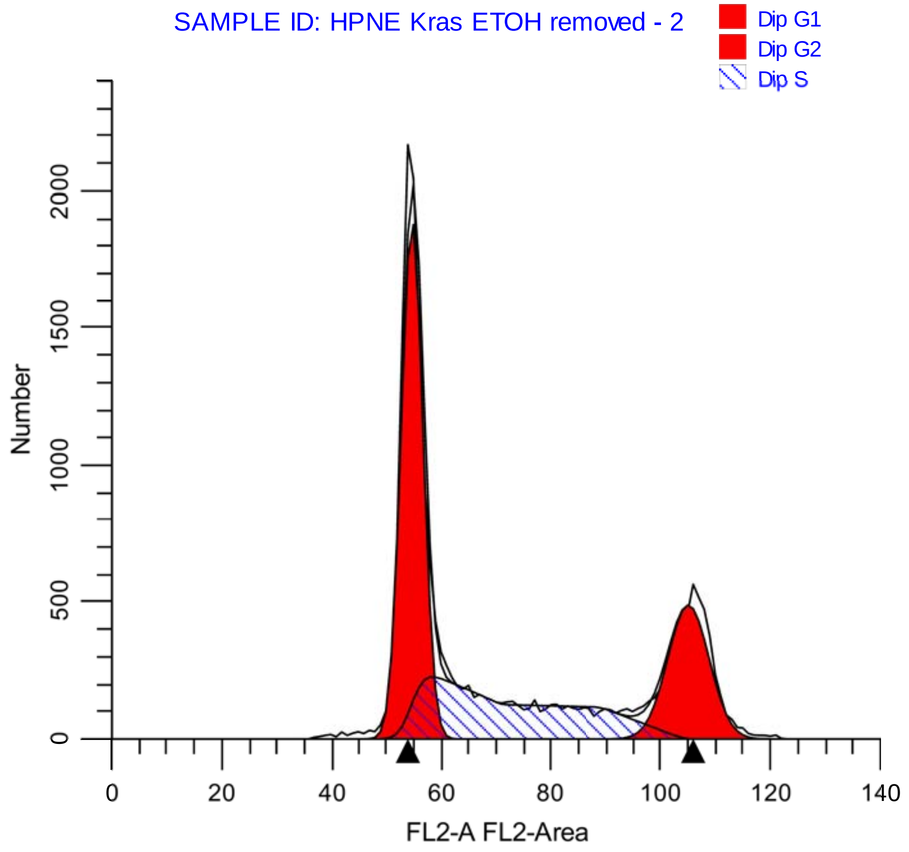

File analyzed: EC13J20.014
Date analyzed: 13-Jan-2020
Model: 1nn0n_DSD
Analysis type: Manual analysis
Auto Linearity: No

Ploidy Mode: First cycle is diploid

Diploid: 100.00 %
Dip G1: 46.21 % at 54.76
Dip G2: 22.70 % at 105.14
Dip S: 31.09 % G2/G1: 1.92
%CV: 3.54

Total S-Phase: 31.09 %
Total B.A.D.: 0.00 % no debris no aggs

Debris: %
Aggregates: %
Modeled events: 20124
All cycle events: 20124
Cycle events per channel: 392
RCS: 5.764

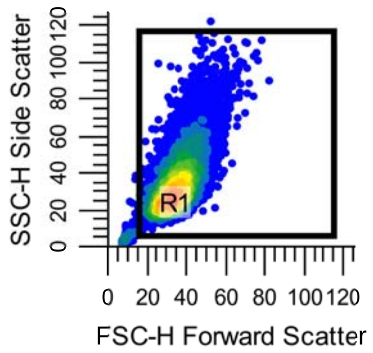

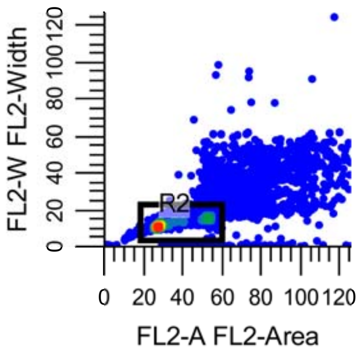

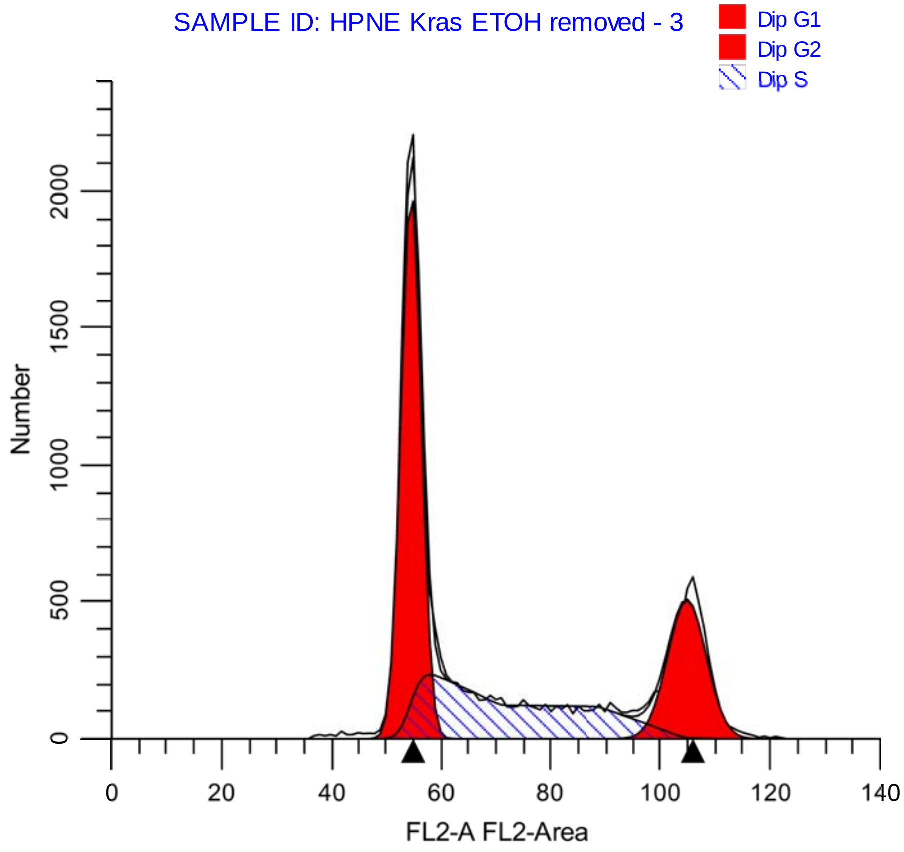

File analyzed: EC13J20.015
Date analyzed: 13-Jan-2020
Model: 1nn0n_DSD
Analysis type: Manual analysis
Auto Linearity: No

Ploidy Mode: First cycle is diploid

Diploid: 100.00 %
Dip G1: 45.97 % at 54.64
Dip G2: 22.09 % at 104.91
Dip S: 31.94 % G2/G1: 1.92
%CV: 3.29

Total S-Phase: 31.94 %
Total B.A.D.: 0.00 % no debris no aggs

Debris: %
Aggregates: %
Modeled events: 19878
All cycle events: 19878
Cycle events per channel: 388
RCS: 3.879

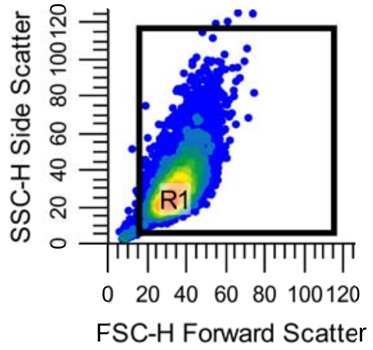

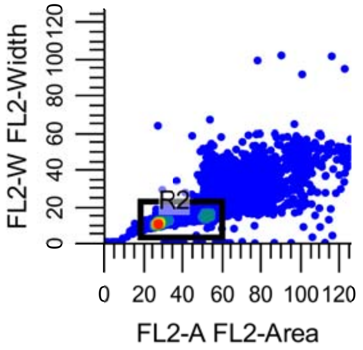

ModFit LT V4.0.5(Win)
