# Supplementary material for: Combined Alcohol Exposure and KRAS Mutation in Human Pancreatic Ductal Epithelial Cells Induces Proliferation and Alters Subtype Signatures Determined by Multi-Omics Analysis
Source: Cancers (Basel). 2022 Apr 13;14(8):1968. doi: 10.3390/cancers14081968 (PMC9027648; doi:10.3390/cancers14081968)
Supplement: Supplementary file 1 [file cancers-14-01968-s001.zip › supplementary/Figure_S2.pdf]

# BD FACSDiva 8.0.1

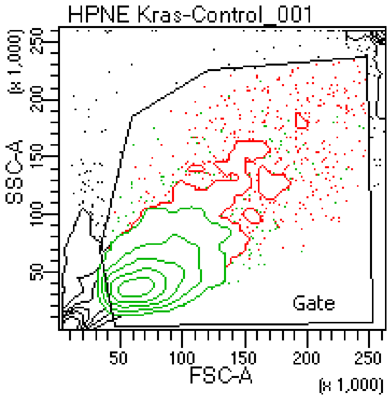


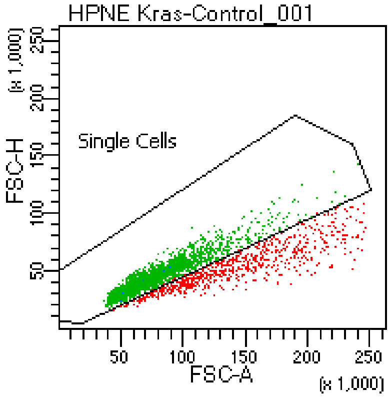


Tube: Control_001

| Population | #Events | %Parent | %Total |
|---|---|---|---|
| All Events | 5,128 | #### | 100.0 |
| Gate | 3,158 | 61.6 | 61.6 |
| Single Cells | 2,422 | 76.7 | 47.2 |
| DCFDA+ | 5 | 0.2 | 0.1 |

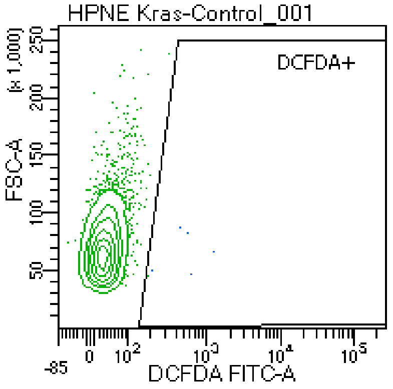


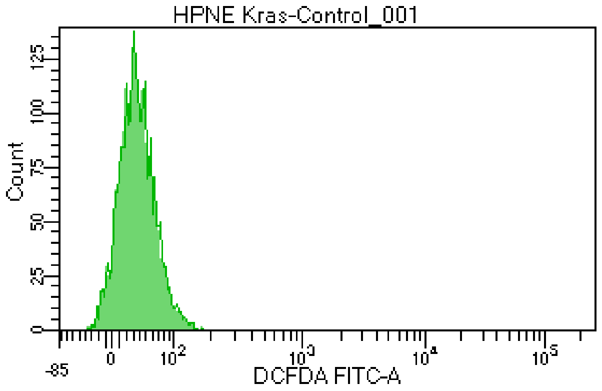


Specimen Name: HPNE Kras    Tube Name: Control_001

| Population | #Events | %Parent | %Total | DCFDA FITC-A Median |
|---|---|---|---|---|
| All Events | 5,128 | #### | 100.0 | 34 |
| Gate | 3,158 | 61.6 | 61.6 | 38 |
| Single Cells | 2,422 | 76.7 | 47.2 | 30 |
| DCFDA+ | 5 | 0.2 | 0.1 | 506 |

# BD FACSDiva 8.0.1

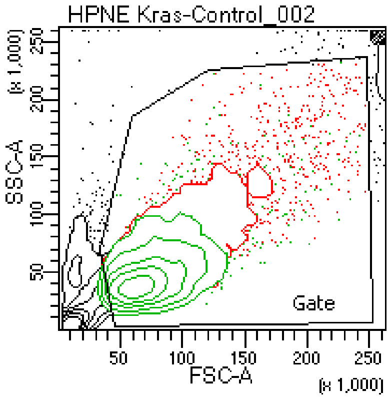


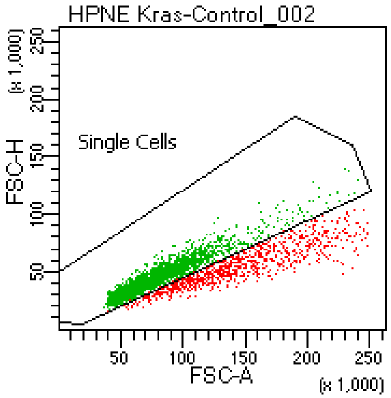


Tube: Control_002

| Population | #Events | %Parent | %Total |
|---|---|---|---|
| All Events | 5,601 | #### | 100.0 |
| Gate | 3,599 | 64.3 | 64.3 |
| Single Cells | 2,728 | 75.8 | 48.7 |
| DCFDA+ | 0 | 0.0 | 0.0 |

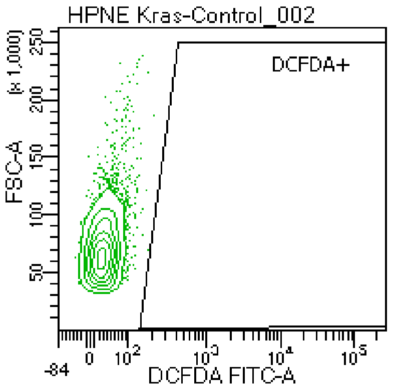


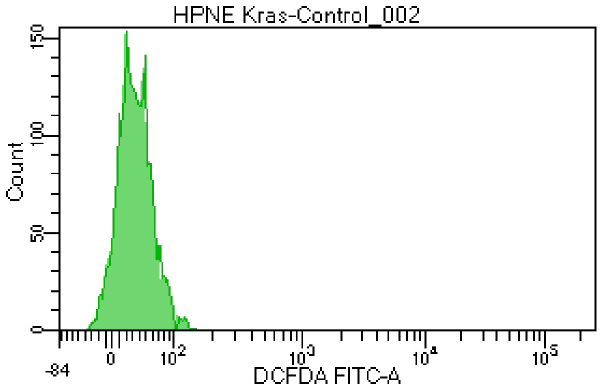


Specimen Name: HPNE Kras    Tube Name: Control_002

| Population | #Events | %Parent | %Total | DCFDA FITC-A Median |
|---|---|---|---|---|
| All Events | 5,601 | #### | 100.0 | 31 |
| Gate | 3,599 | 64.3 | 64.3 | 36 |
| Single Cells | 2,728 | 75.8 | 48.7 | 27 |
| DCFDA+ | 0 | 0.0 | 0.0 | #### |

# BD FACSDiva 8.0.1

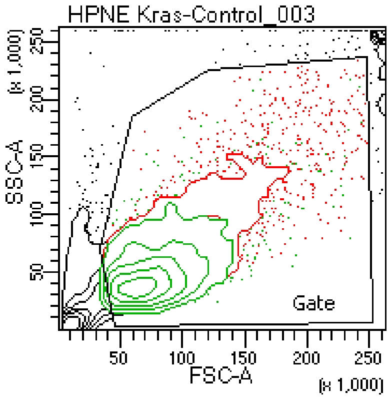


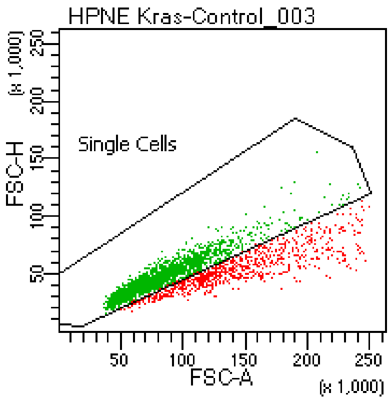


Tube: Control_003

| Population | #Events | %Parent | %Total |
|---|---|---|---|
| All Events | 5,269 | #### | 100.0 |
| Gate | 3,307 | 62.8 | 62.8 |
| Single Cells | 2,487 | 75.2 | 47.2 |
| DCFDA+ | 0 | 0.0 | 0.0 |

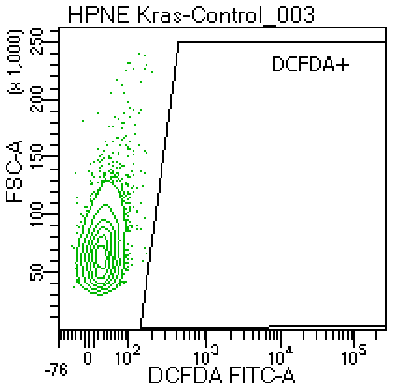


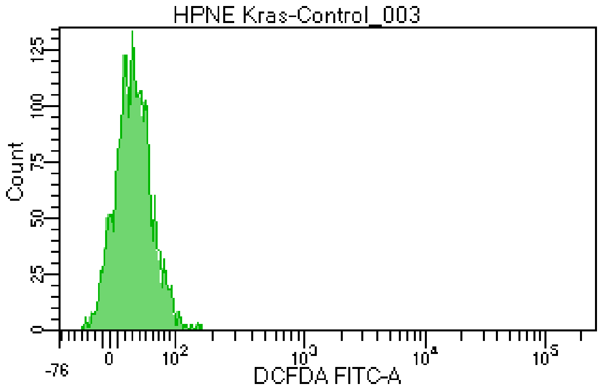


Specimen Name: HPNE Kras    Tube Name: Control_003

| Population | #Events | %Parent | %Total | DCFDA FITC-A Median |
|---|---|---|---|---|
| All Events | 5,269 | #### | 100.0 | 31 |
| Gate | 3,307 | 62.8 | 62.8 | 35 |
| Single Cells | 2,487 | 75.2 | 47.2 | 26 |
| DCFDA+ | 0 | 0.0 | 0.0 | #### |

BD FACSDiva 8.0.1

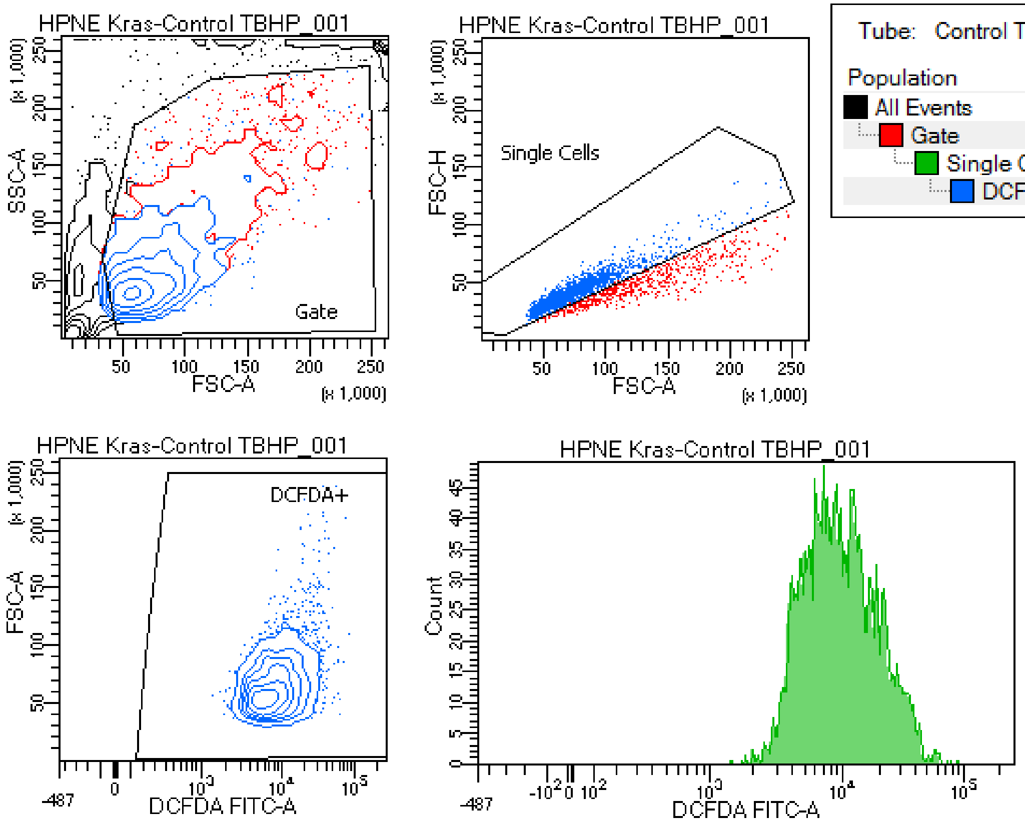


Tube: Control TBHP_001

| Population | #Events | %Parent | %Total |
|---|---|---|---|
| All Events | 4,195 | #### | 100.0 |
| Gate | 2,573 | 61.3 | 61.3 |
| Single Cells | 1,901 | 73.9 | 45.3 |
| DCFDA+ | 1,901 | 100.0 | 45.3 |

Specimen Name: HPNE Kras    Tube Name: Control TBHP_001

| Population | #Events | %Parent | %Total | DCFDA FITC-A Median |
|---|---|---|---|---|
| All Events | 4,195 | #### | 100.0 | 10,756 |
| Gate | 2,573 | 61.3 | 61.3 | 11,588 |
| Single Cells | 1,901 | 73.9 | 45.3 | 8,549 |
| DCFDA+ | 1,901 | 100.0 | 45.3 | 8,549 |

# BD FACSDiva 8.0.1

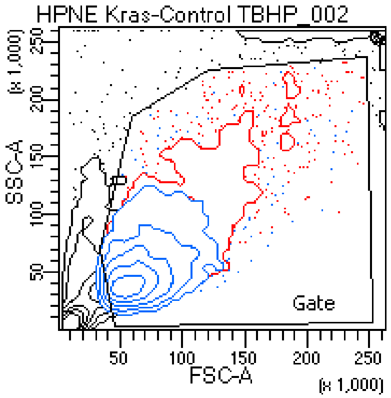


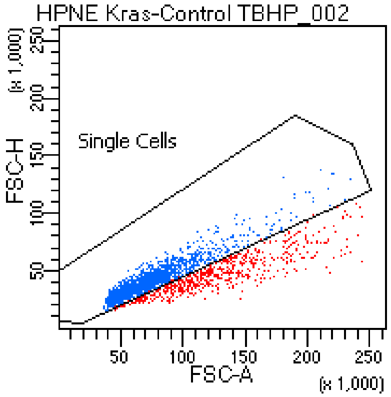


Tube: Control TBHP_002

| Population | #Events | %Parent | %Total |
|---|---|---|---|
| All Events | 4,093 | #### | 100.0 |
| Gate | 2,356 | 57.6 | 57.6 |
| Single Cells | 1,732 | 73.5 | 42.3 |
| DCFDA+ | 1,732 | 100.0 | 42.3 |

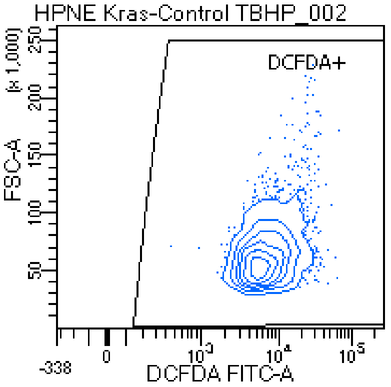


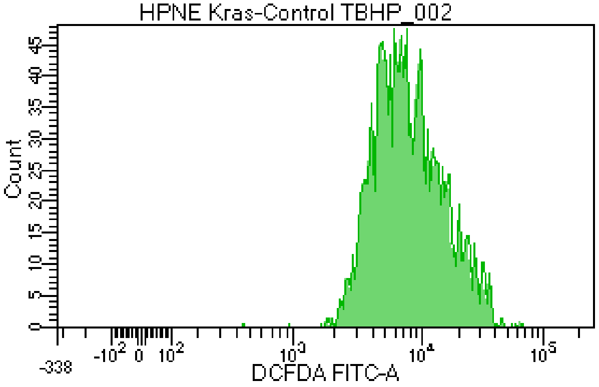


Specimen Name: HPNE Kras    Tube Name: Control TBHP_002

| Population | #Events | %Parent | %Total | DCFDA FITC-A Median |
|---|---|---|---|---|
| All Events | 4,093 | #### | 100.0 | 8,172 |
| Gate | 2,356 | 57.6 | 57.6 | 9,189 |
| Single Cells | 1,732 | 73.5 | 42.3 | 6,733 |
| DCFDA+ | 1,732 | 100.0 | 42.3 | 6,733 |

BD FACSDiva 8.0.1

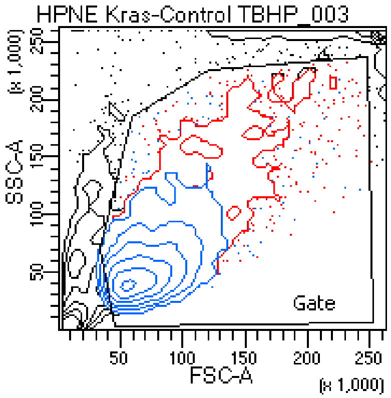


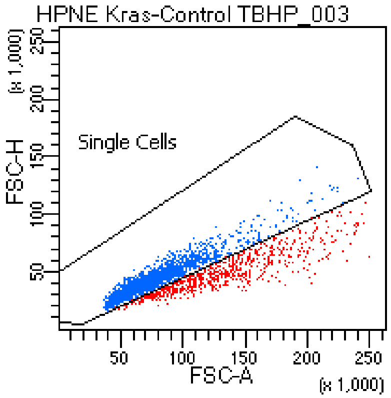


Tube: Control TBHP_003

| Population | #Events | %Parent | %Total |
|---|---|---|---|
| All Events | 4,830 | #### | 100.0 |
| Gate | 2,915 | 60.4 | 60.4 |
| Single Cells | 2,200 | 75.5 | 45.5 |
| DCFDA+ | 2,200 | 100.0 | 45.5 |

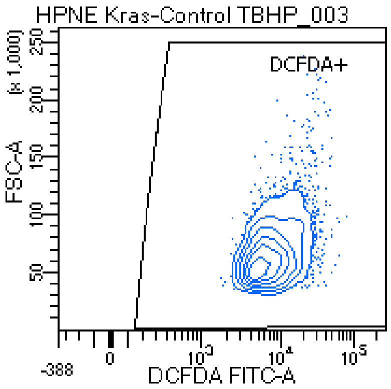


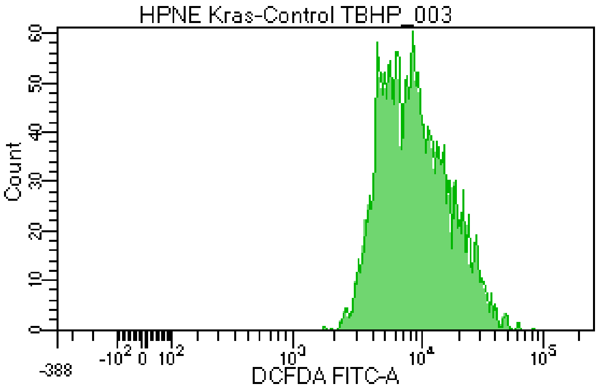


| Specimen Name: | HPNE Kras | Tube Name: | Control TBHP_003 | |
|---|---|---|---|---|
| Population | #Events | %Parent | %Total | DCFDA FITC-A Median |
| All Events | 4,830 | #### | 100.0 | 9,398 |
| Gate | 2,915 | 60.4 | 60.4 | 10,177 |
| Single Cells | 2,200 | 75.5 | 45.5 | 7,651 |
| DCFDA+ | 2,200 | 100.0 | 45.5 | 7,651 |

BD FACSDiva 8.0.1

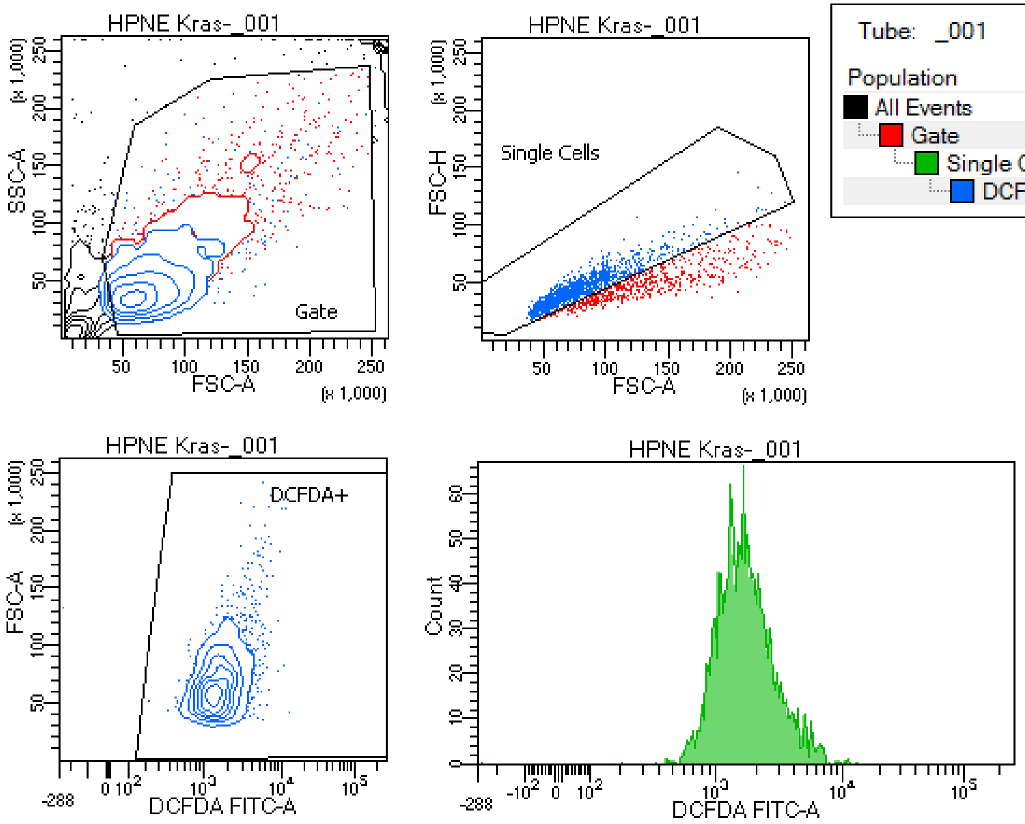


Tube: _001

| Population | #Events | %Parent | %Total |
|---|---|---|---|
| All Events | 3,733 | #### | 100.0 |
| Gate | 2,167 | 58.0 | 58.0 |
| Single Cells | 1,590 | 73.4 | 42.6 |
| DCFDA+ | 1,589 | 99.9 | 42.6 |

Specimen Name: HPNE Kras Tube Name: _001

| Population | #Events | %Parent | %Total | DCFDA FITC-A Median |
|---|---|---|---|---|
| All Events | 3,733 | #### | 100.0 | 1,563 |
| Gate | 2,167 | 58.0 | 58.0 | 1,888 |
| Single Cells | 1,590 | 73.4 | 42.6 | 1,508 |
| DCFDA+ | 1,589 | 99.9 | 42.6 | 1,508 |

BD FACSDiva 8.0.1

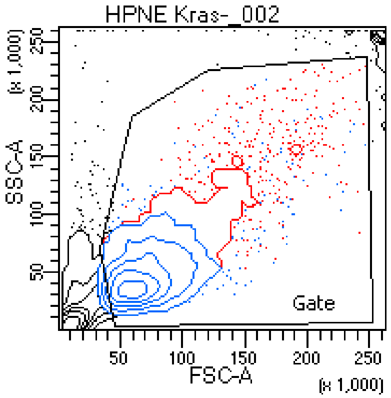


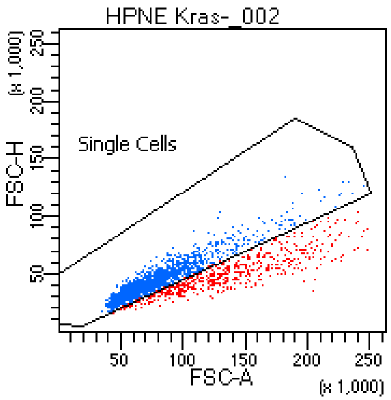


Tube: _002

| Population | #Events | %Parent | %Total |
|---|---|---|---|
| All Events | 3,969 | #### | 100.0 |
| Gate | 2,373 | 59.8 | 59.8 |
| Single Cells | 1,773 | 74.7 | 44.7 |
| DCFDA+ | 1,771 | 99.9 | 44.6 |

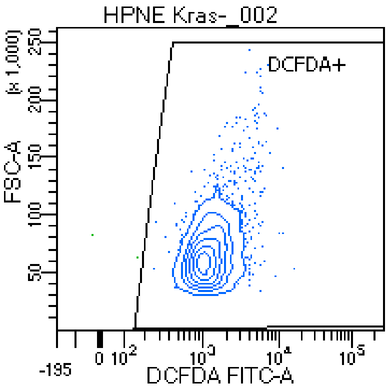


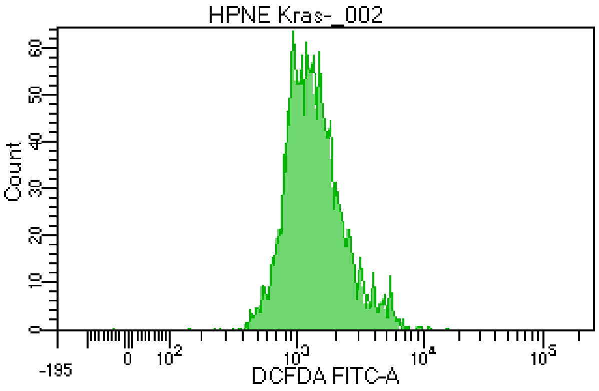


Specimen Name: HPNE Kras Tube Name: _002

| Population | #Events | %Parent | %Total | DCFDA FITC-A Median |
|---|---|---|---|---|
| All Events | 3,969 | #### | 100.0 | 1,236 |
| Gate | 2,373 | 59.8 | 59.8 | 1,477 |
| Single Cells | 1,773 | 74.7 | 44.7 | 1,195 |
| DCFDA+ | 1,771 | 99.9 | 44.6 | 1,196 |

# BD FACSDiva 8.0.1

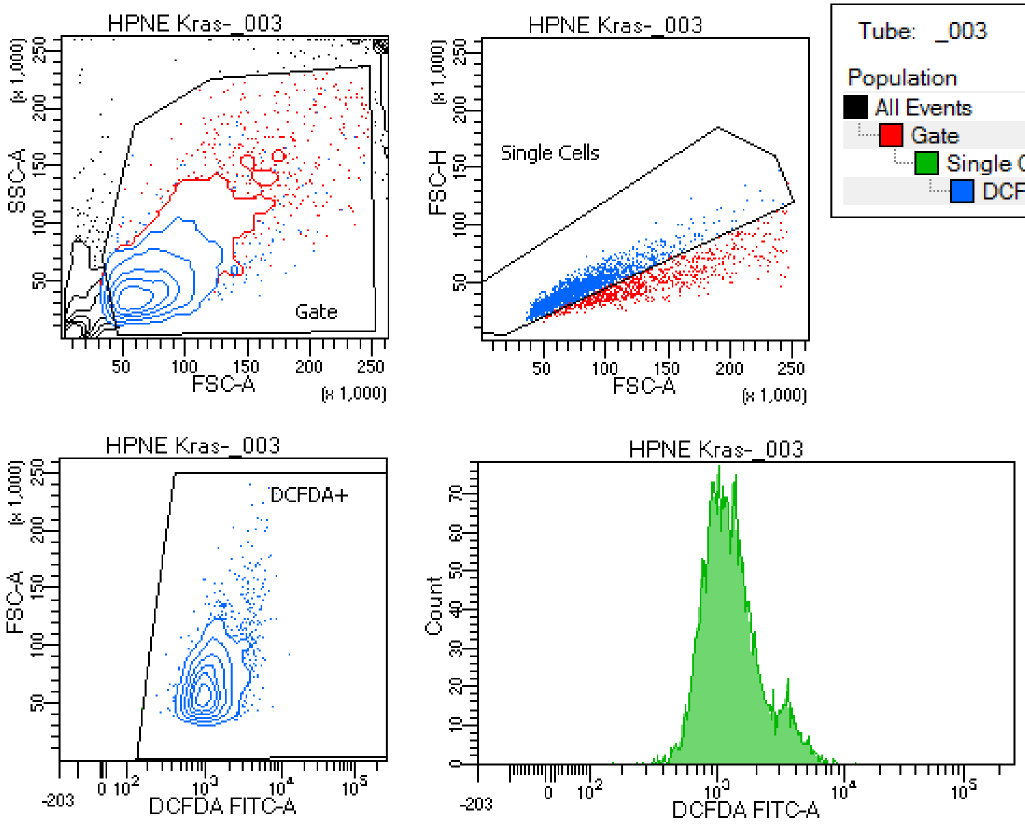


Tube: _003

| Population | #Events | %Parent | %Total |
|---|---|---|---|
| All Events | 4,556 | #### | 100.0 |
| Gate | 2,914 | 64.0 | 64.0 |
| Single Cells | 2,155 | 74.0 | 47.3 |
| DCFDA+ | 2,154 | 100.0 | 47.3 |

Specimen Name: HPNE Kras Tube Name: _003

| Population | #Events | %Parent | %Total | DCFDA FITC-A Median |
|---|---|---|---|---|
| All Events | 4,556 | #### | 100.0 | 1,216 |
| Gate | 2,914 | 64.0 | 64.0 | 1,386 |
| Single Cells | 2,155 | 74.0 | 47.3 | 1,112 |
| DCFDA+ | 2,154 | 100.0 | 47.3 | 1,113 |

# BD FACSDiva 8.0.1

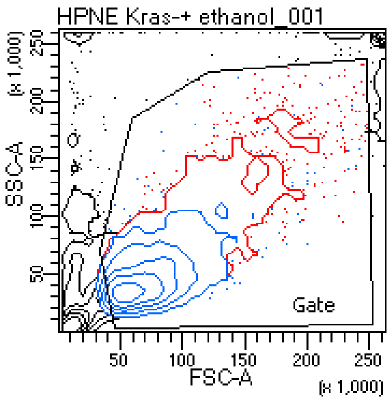


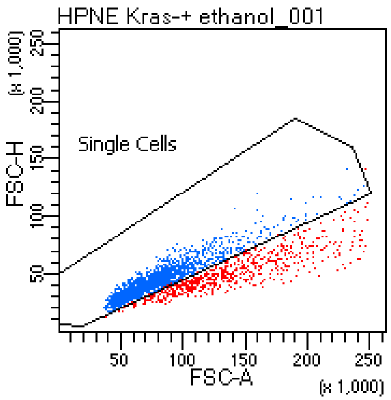


Tube: + ethanol_001

| Population | #Events | %Parent | %Total |
|---|---|---|---|
| All Events | 4,362 | #### | 100.0 |
| Gate | 2,627 | 60.2 | 60.2 |
| Single Cells | 1,922 | 73.2 | 44.1 |
| DCFDA+ | 1,921 | 99.9 | 44.0 |

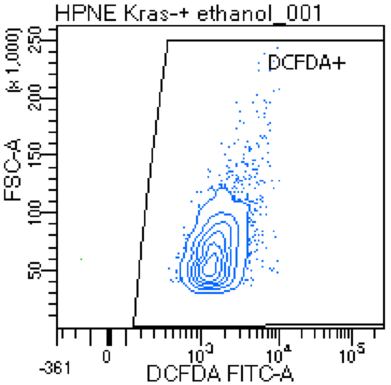


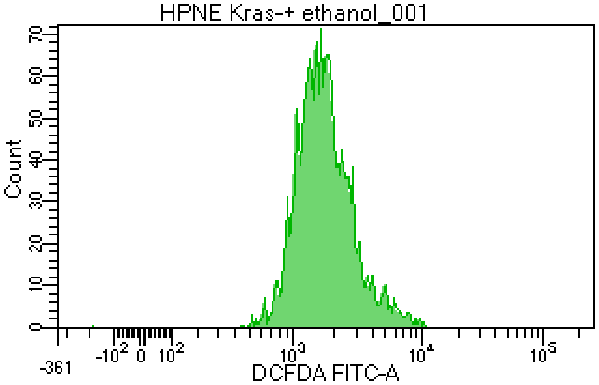


Specimen Name: HPNE Kras    Tube Name: + ethanol_001

| Population | #Events | %Parent | %Total | DCFDA FITC-A Median |
|---|---|---|---|---|
| All Events | 4,362 | #### | 100.0 | 1,662 |
| Gate | 2,627 | 60.2 | 60.2 | 1,886 |
| Single Cells | 1,922 | 73.2 | 44.1 | 1,529 |
| DCFDA+ | 1,921 | 99.9 | 44.0 | 1,529 |

# BD FACSDiva 8.0.1

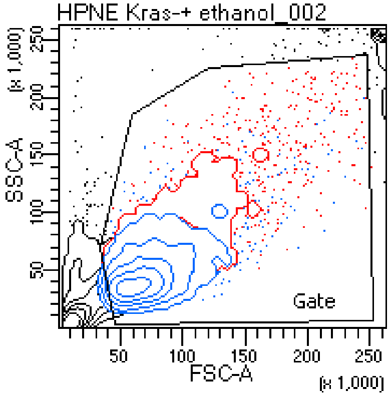


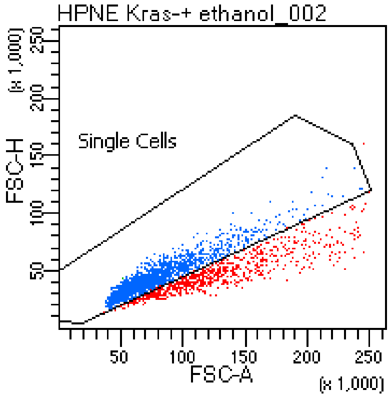


| Tube: + ethanol_002 | | | |
|---|---|---|---|
| Population | #Events | %Parent | %Total |
| All Events | 4,287 | #### | 100.0 |
| Gate | 2,604 | 60.7 | 60.7 |
| Single Cells | 1,865 | 71.6 | 43.5 |
| DCFDA+ | 1,864 | 99.9 | 43.5 |

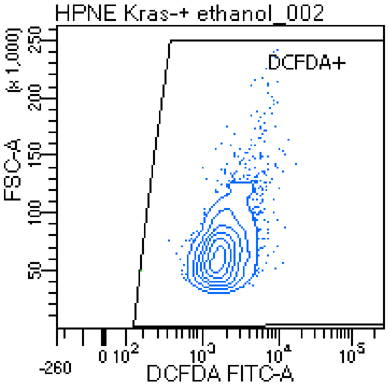


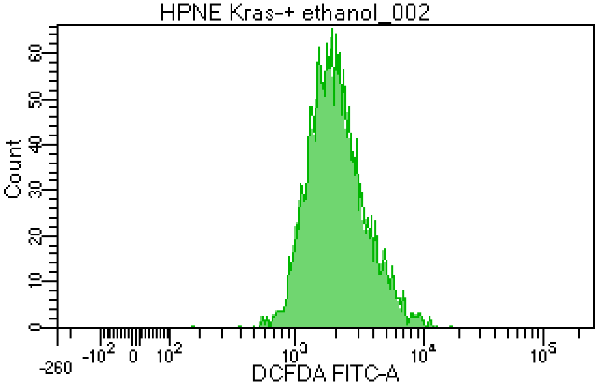


Specimen Name: HPNE Kras　　Tube Name: + ethanol_002

| Population | #Events | %Parent | %Total | DCFDA FITC-A Median |
|---|---|---|---|---|
| All Events | 4,287 | #### | 100.0 | 1,928 |
| Gate | 2,604 | 60.7 | 60.7 | 2,346 |
| Single Cells | 1,865 | 71.6 | 43.5 | 1,831 |
| DCFDA+ | 1,864 | 99.9 | 43.5 | 1,831 |

# BD FACSDiva 8.0.1

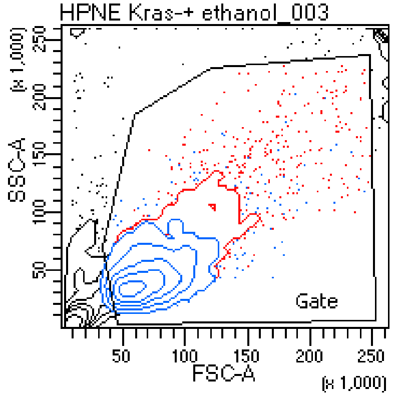


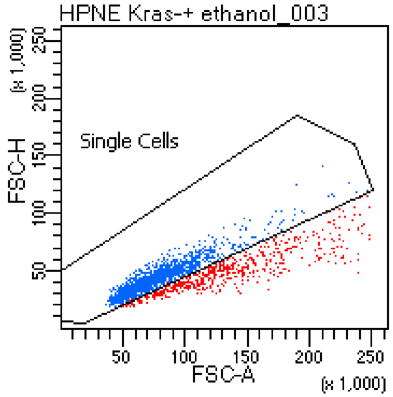


Tube: + ethanol_003

| Population | #Events | %Parent | %Total |
|---|---|---|---|
| All Events | 3,583 | #### | 100.0 |
| Gate | 2,083 | 58.1 | 58.1 |
| Single Cells | 1,498 | 71.9 | 41.8 |
| DCFDA+ | 1,498 | 100.0 | 41.8 |

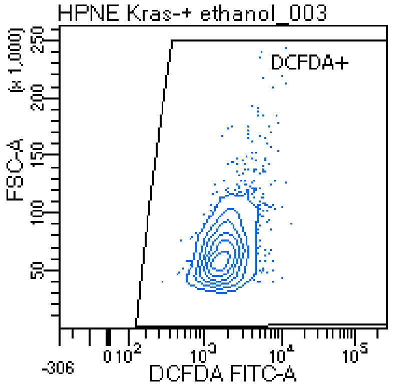


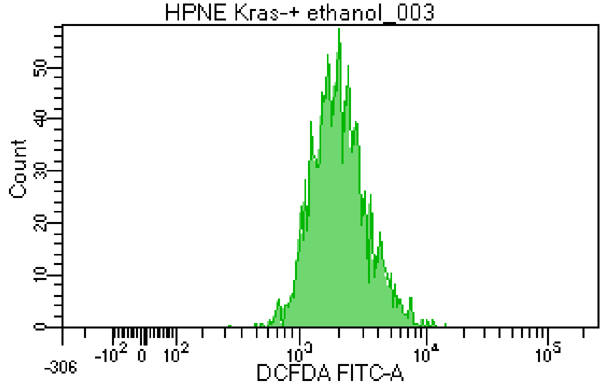


| Specimen Name: | HPNE Kras | Tube Name: | + ethanol_003 | |
|---|---|---|---|---|
| Population | #Events | %Parent | %Total | DCFDA FITC-A Median |
| All Events | 3,583 | #### | 100.0 | 1,831 |
| Gate | 2,083 | 58.1 | 58.1 | 2,290 |
| Single Cells | 1,498 | 71.9 | 41.8 | 1,822 |
| DCFDA+ | 1,498 | 100.0 | 41.8 | 1,822 |

# BD FACSDiva 8.0.1

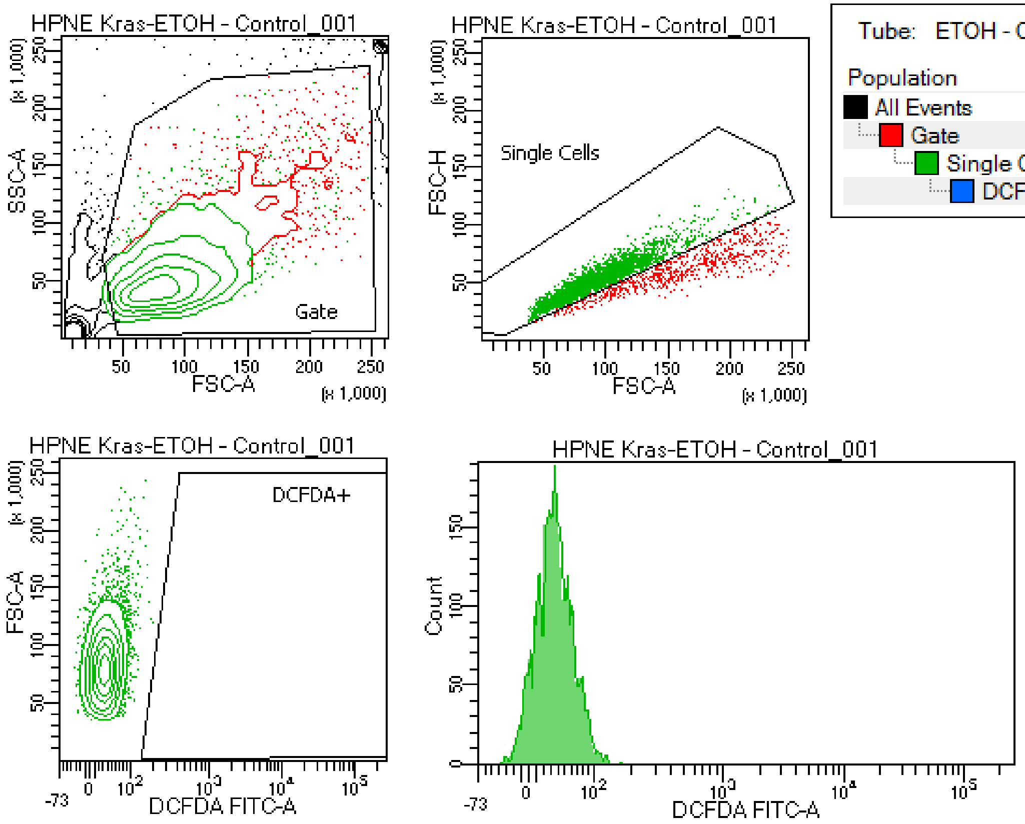


Tube: ETOH - Control_001

| Population | #Events | %Parent | %Total |
|---|---|---|---|
| All Events | 5,602 | #### | 100.0 |
| Gate | 4,042 | 72.2 | 72.2 |
| Single Cells | 3,377 | 83.5 | 60.3 |
| DCFDA+ | 0 | 0.0 | 0.0 |

Specimen Name: HPNE Kras    Tube Name: ETOH - Control_001

| Population | #Events | %Parent | %Total | DCFDA FITC-A Median |
|---|---|---|---|---|
| All Events | 5,602 | #### | 100.0 | 34 |
| Gate | 4,042 | 72.2 | 72.2 | 35 |
| Single Cells | 3,377 | 83.5 | 60.3 | 30 |
| DCFDA+ | 0 | 0.0 | 0.0 | #### |

# BD FACSDiva 8.0.1

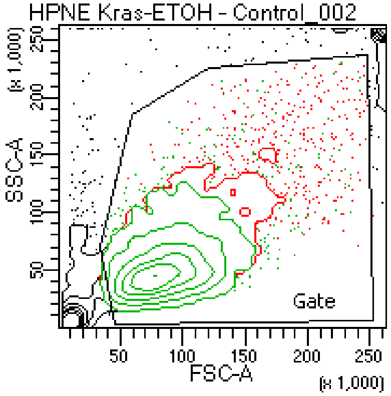


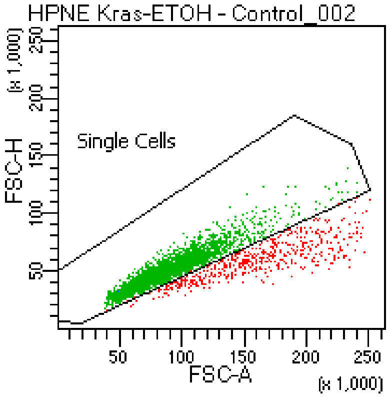


Tube: ETOH - Control_002

| Population | #Events | %Parent | %Total |
|---|---|---|---|
| All Events | 5,157 | #### | 100.0 |
| Gate | 3,659 | 71.0 | 71.0 |
| Single Cells | 3,114 | 85.1 | 60.4 |
| DCFDA+ | 1 | 0.0 | 0.0 |

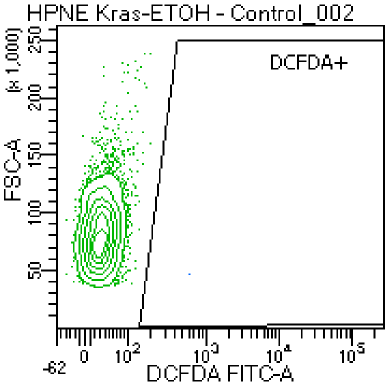


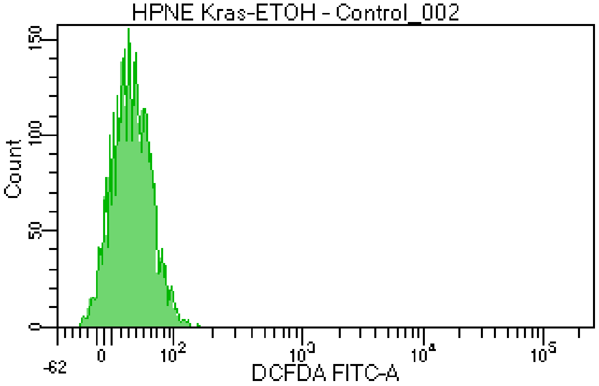


Specimen Name: HPNE Kras Tube Name: ETOH - Control_002

| Population | #Events | %Parent | %Total | DCFDA FITC-A Median |
|---|---|---|---|---|
| All Events | 5,157 | #### | 100.0 | 32 |
| Gate | 3,659 | 71.0 | 71.0 | 34 |
| Single Cells | 3,114 | 85.1 | 60.4 | 29 |
| DCFDA+ | 1 | 0.0 | 0.0 | 569 |

BD FACSDiva 8.0.1

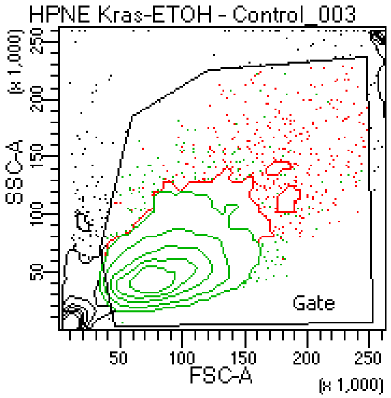


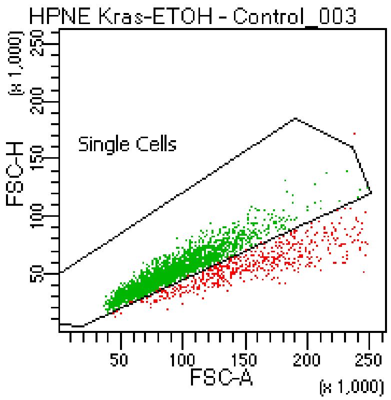


Tube: ETOH - Control_003

| Population | #Events | %Parent | %Total |
|---|---|---|---|
| All Events | 5,244 | #### | 100.0 |
| Gate | 3,757 | 71.6 | 71.6 |
| Single Cells | 3,204 | 85.3 | 61.1 |
| DCFDA+ | 0 | 0.0 | 0.0 |

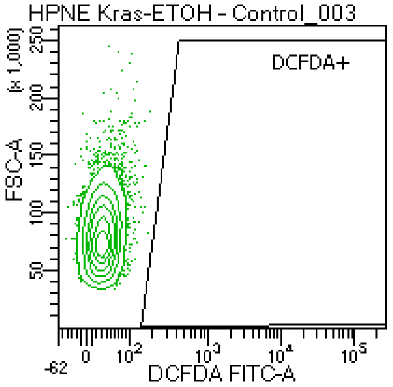


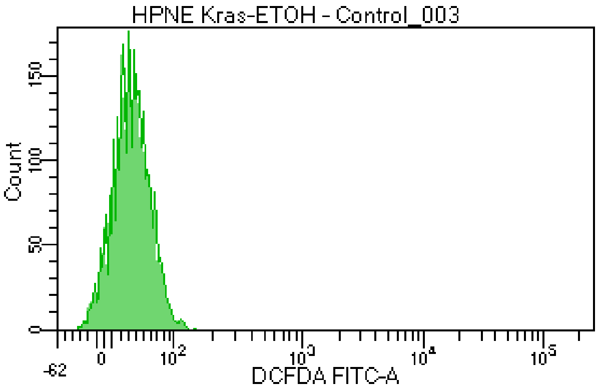


Specimen Name: HPNE Kras Tube Name: ETOH - Control_003

| Population | #Events | %Parent | %Total | DCFDA FITC-A Median |
|---|---|---|---|---|
| All Events | 5,244 | #### | 100.0 | 31 |
| Gate | 3,757 | 71.6 | 71.6 | 33 |
| Single Cells | 3,204 | 85.3 | 61.1 | 29 |
| DCFDA+ | 0 | 0.0 | 0.0 | #### |

# BD FACSDiva 8.0.1

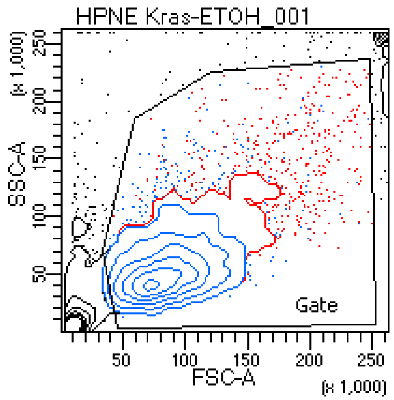


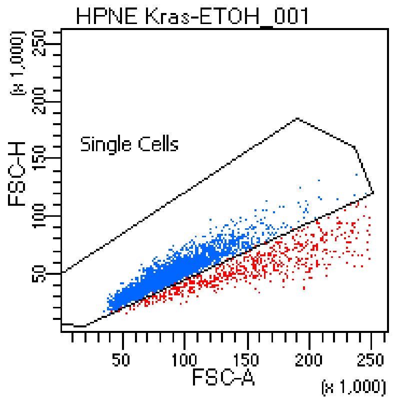


Tube: ETOH_001

| Population | #Events | %Parent | %Total |
|---|---|---|---|
| All Events | 5,694 | #### | 100.0 |
| Gate | 4,216 | 74.0 | 74.0 |
| Single Cells | 3,497 | 82.9 | 61.4 |
| DCFDA+ | 3,496 | 100.0 | 61.4 |

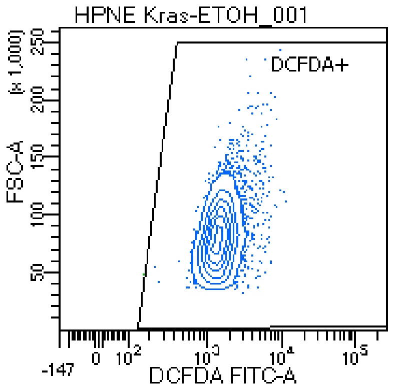


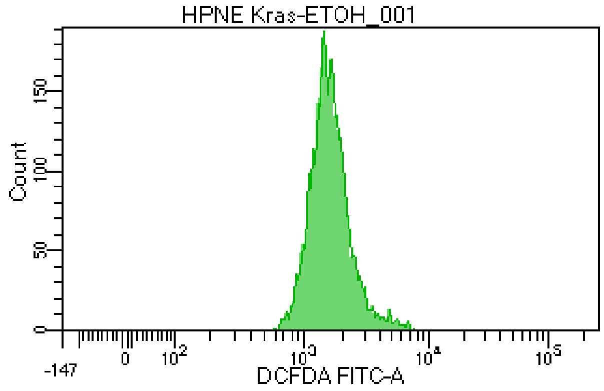


Specimen Name: HPNE Kras    Tube Name: ETOH_001

| Population | #Events | %Parent | %Total | DCFDA FITC-A Median |
|---|---|---|---|---|
| All Events | 5,694 | #### | 100.0 | 1,500 |
| Gate | 4,216 | 74.0 | 74.0 | 1,566 |
| Single Cells | 3,497 | 82.9 | 61.4 | 1,446 |
| DCFDA+ | 3,496 | 100.0 | 61.4 | 1,447 |

BD FACSDiva 8.0.1

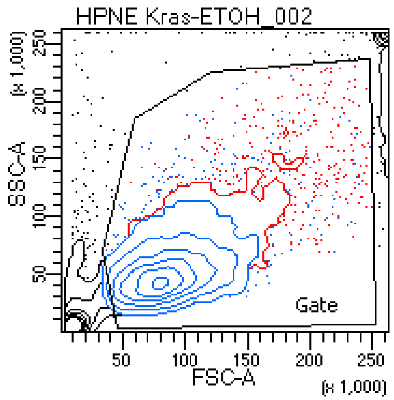


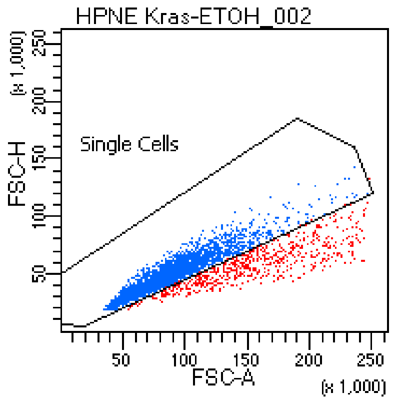


Tube: ETOH_002

| Population | #Events | %Parent | %Total |
|---|---|---|---|
| All Events | 4,986 | #### | 100.0 |
| Gate | 3,727 | 74.7 | 74.7 |
| Single Cells | 3,076 | 82.5 | 61.7 |
| DCFDA+ | 3,076 | 100.0 | 61.7 |

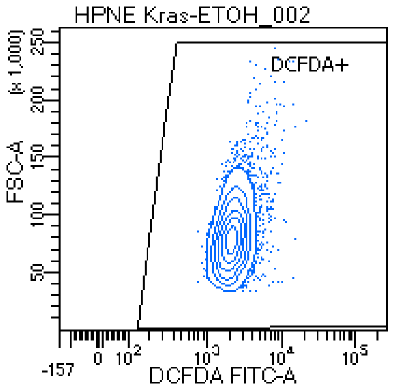


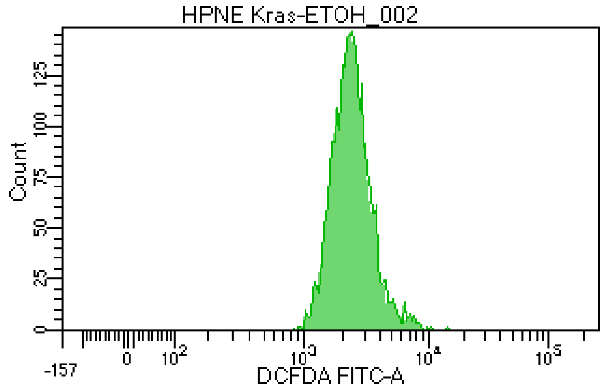


Specimen Name: HPNE Kras Tube Name: ETOH_002

| Population | #Events | %Parent | %Total | DCFDA FITC-A Median |
|---|---|---|---|---|
| All Events | 4,986 | #### | 100.0 | 2,267 |
| Gate | 3,727 | 74.7 | 74.7 | 2,372 |
| Single Cells | 3,076 | 82.5 | 61.7 | 2,180 |
| DCFDA+ | 3,076 | 100.0 | 61.7 | 2,180 |

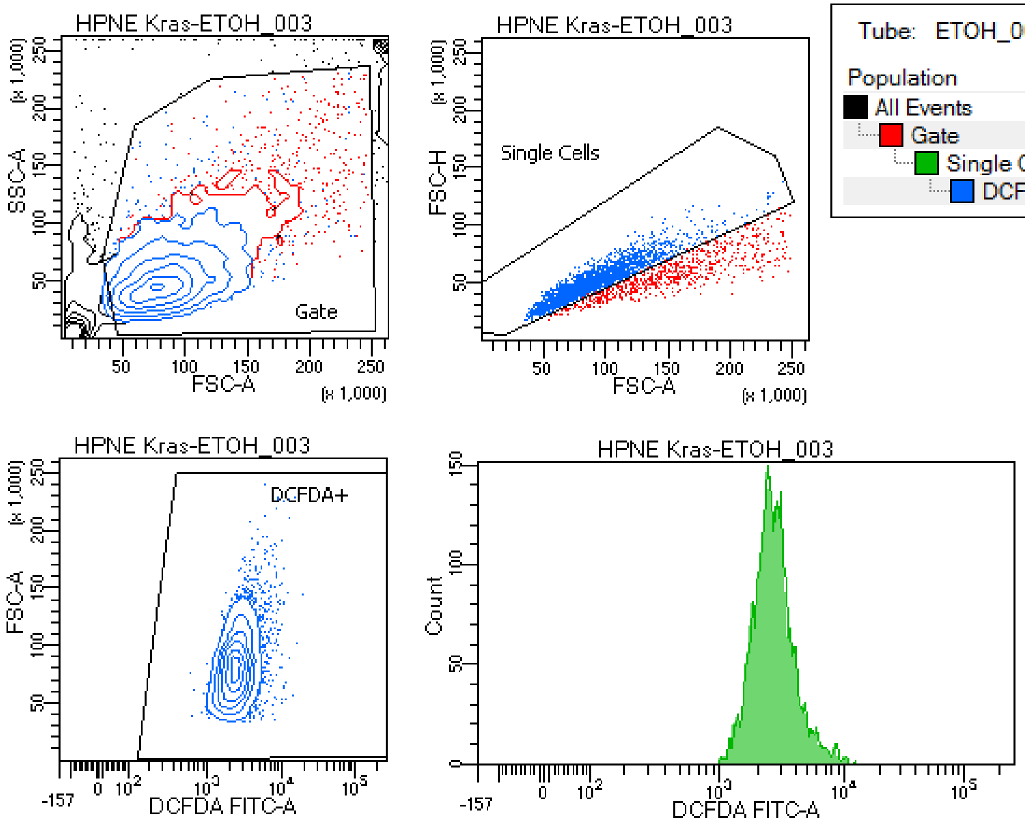


Tube: ETOH_003

| Population | #Events | %Parent | %Total |
|---|---|---|---|
| All Events | 5,043 | #### | 100.0 |
| Gate | 3,643 | 72.2 | 72.2 |
| Single Cells | 2,968 | 81.5 | 58.9 |
| DCFDA+ | 2,968 | 100.0 | 58.9 |

Specimen Name: HPNE Kras Tube Name: ETOH_003

| Population | #Events | %Parent | %Total | DCFDA FITC-A Median |
|---|---|---|---|---|
| All Events | 5,043 | #### | 100.0 | 2,578 |
| Gate | 3,643 | 72.2 | 72.2 | 2,682 |
| Single Cells | 2,968 | 81.5 | 58.9 | 2,426 |
| DCFDA+ | 2,968 | 100.0 | 58.9 | 2,426 |

# BD FACSDiva 8.0.1

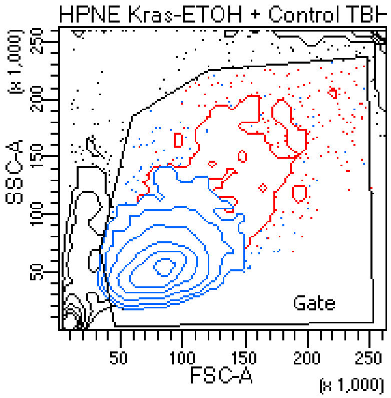


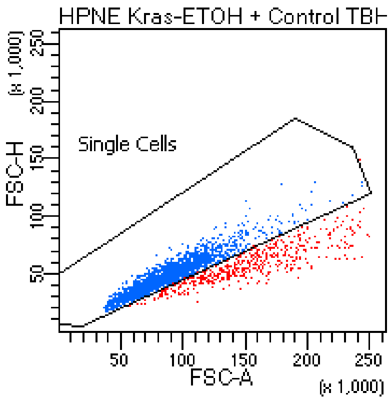


Tube: ETOH + Control TBHP_001

| Population | #Events | %Parent | %Total |
|---|---|---|---|
| All Events | 4,240 | #### | 100.0 |
| Gate | 2,935 | 69.2 | 69.2 |
| Single Cells | 2,399 | 81.7 | 56.6 |
| DCFDA+ | 2,399 | 100.0 | 56.6 |

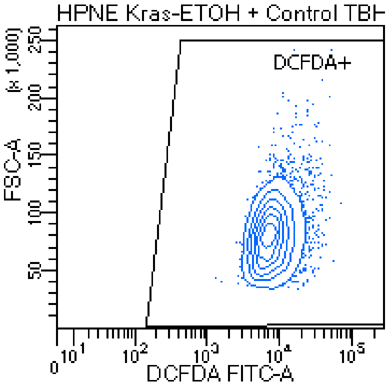


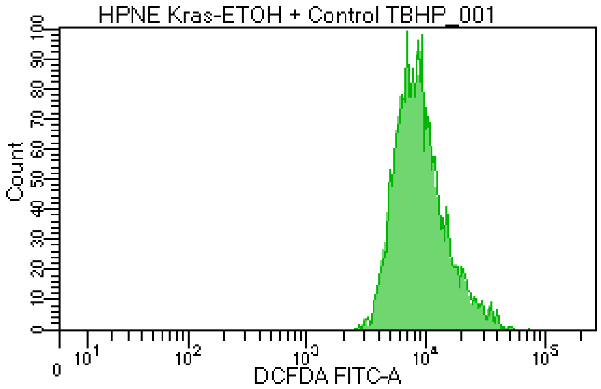


| Specimen Name: | HPNE Kras | Tube Name: | ETOH + Control TBHP_001 | |
|---|---|---|---|---|
| Population | #Events | %Parent | %Total | DCFDA FITC-A Median |
| All Events | 4,240 | #### | 100.0 | 9,179 |
| Gate | 2,935 | 69.2 | 69.2 | 8,917 |
| Single Cells | 2,399 | 81.7 | 56.6 | 7,907 |
| DCFDA+ | 2,399 | 100.0 | 56.6 | 7,907 |

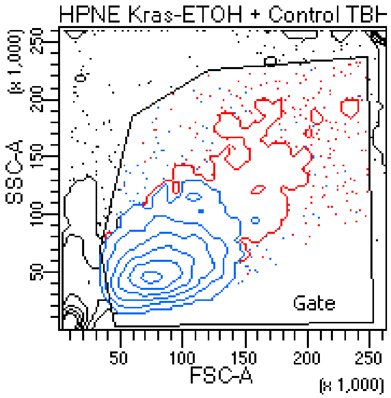


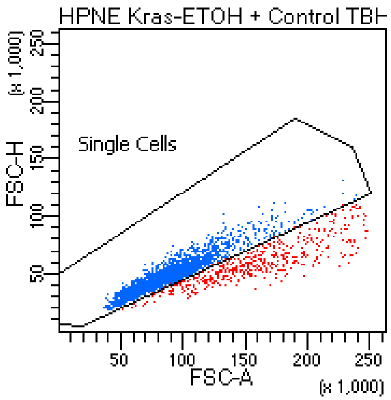


Tube: ETOH + Control TBHP_002

| Population | #Events | %Parent | %Total |
|---|---|---|---|
| All Events | 4,037 | #### | 100.0 |
| Gate | 2,739 | 67.8 | 67.8 |
| Single Cells | 2,231 | 81.5 | 55.3 |
| DCFDA+ | 2,231 | 100.0 | 55.3 |

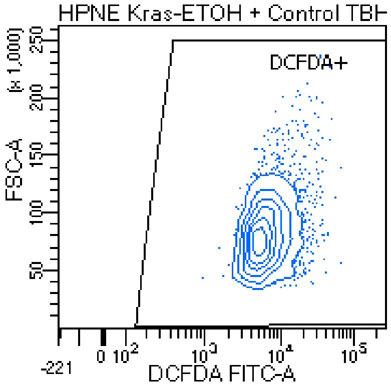


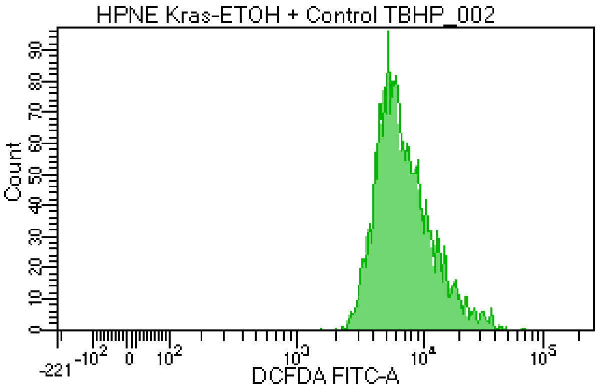


Specimen Name: HPNE Kras    Tube Name: ETOH + Control TBHP_002

| Population | #Events | %Parent | %Total | DCFDA FITC-A Median |
|---|---|---|---|---|
| All Events | 4,037 | #### | 100.0 | 6,940 |
| Gate | 2,739 | 67.8 | 67.8 | 7,083 |
| Single Cells | 2,231 | 81.5 | 55.3 | 5,965 |
| DCFDA+ | 2,231 | 100.0 | 55.3 | 5,965 |

# BD FACSDiva 8.0.1

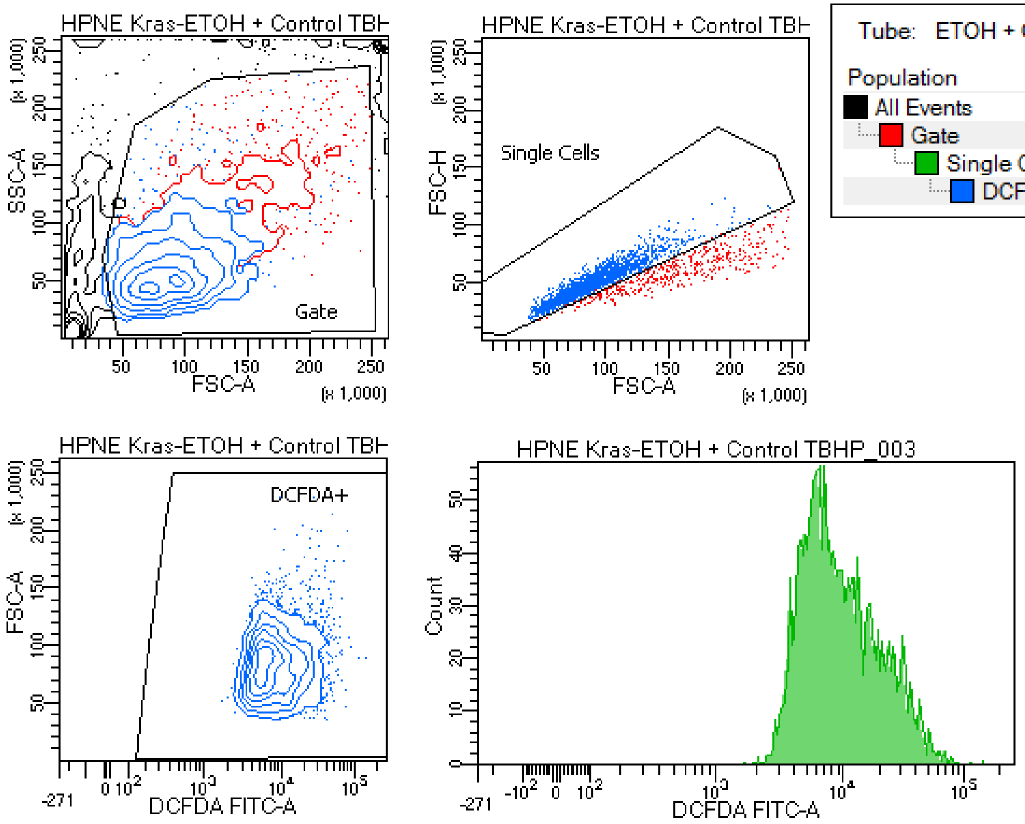


Tube: ETOH + Control TBHP_003

| Population | #Events | %Parent | %Total |
|---|---|---|---|
| All Events | 3,637 | #### | 100.0 |
| Gate | 2,506 | 68.9 | 68.9 |
| Single Cells | 2,070 | 82.6 | 56.9 |
| DCFDA+ | 2,070 | 100.0 | 56.9 |

Specimen Name: HPNE Kras    Tube Name: ETOH + Control TBHP_003

| Population | #Events | %Parent | %Total | DCFDA FITC-A Median |
|---|---|---|---|---|
| All Events | 3,637 | #### | 100.0 | 9,723 |
| Gate | 2,506 | 68.9 | 68.9 | 9,914 |
| Single Cells | 2,070 | 82.6 | 56.9 | 7,843 |
| DCFDA+ | 2,070 | 100.0 | 56.9 | 7,843 |

BD FACSDiva 8.0.1

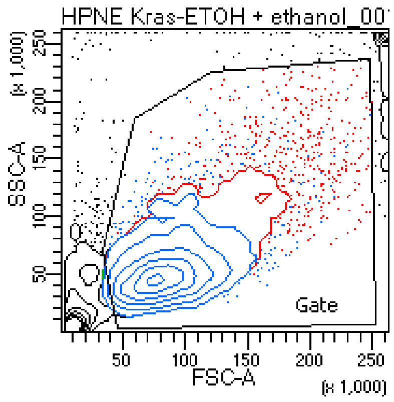


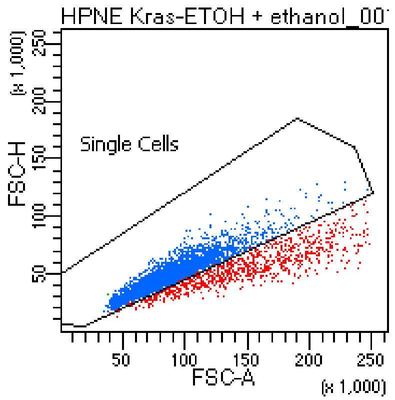


Tube: ETOH + ethanol_001

| Population | #Events | %Parent | %Total |
|---|---|---|---|
| All Events | 6,308 | #### | 100.0 |
| Gate | 4,690 | 74.4 | 74.4 |
| Single Cells | 3,787 | 80.7 | 60.0 |
| DCFDA+ | 3,785 | 99.9 | 60.0 |

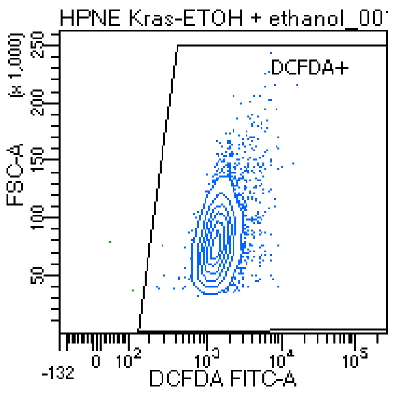


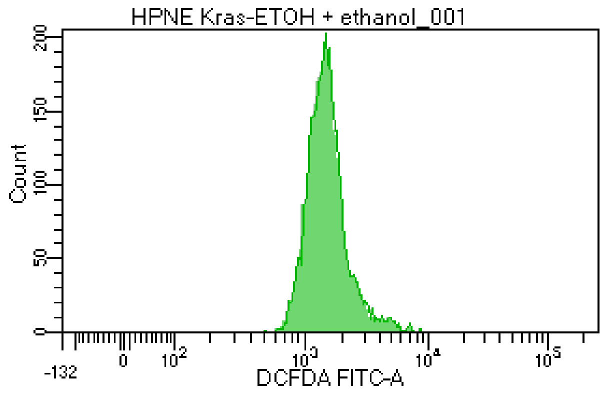


| Specimen Name: | HPNE Kras | Tube Name: | ETOH + ethanol_001 | |
|---|---|---|---|---|
| Population | #Events | %Parent | %Total | DCFDA FITC-A Median |
| All Events | 6,308 | #### | 100.0 | 1,426 |
| Gate | 4,690 | 74.4 | 74.4 | 1,485 |
| Single Cells | 3,787 | 80.7 | 60.0 | 1,357 |
| DCFDA+ | 3,785 | 99.9 | 60.0 | 1,357 |

# BD FACSDiva 8.0.1

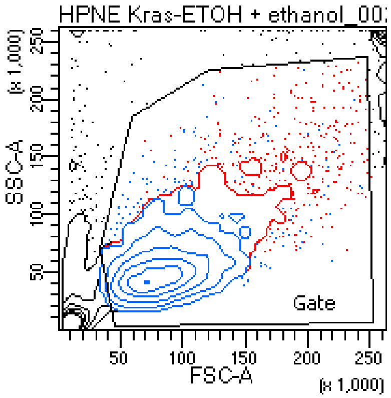


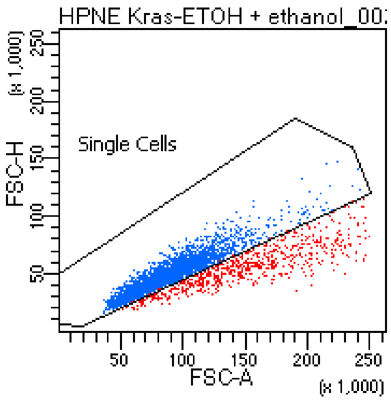


Tube: ETOH + ethanol_002

| Population | #Events | %Parent | %Total |
|---|---|---|---|
| All Events | 5,035 | #### | 100.0 |
| Gate | 3,590 | 71.3 | 71.3 |
| Single Cells | 2,874 | 80.1 | 57.1 |
| DCFDA+ | 2,874 | 100.0 | 57.1 |

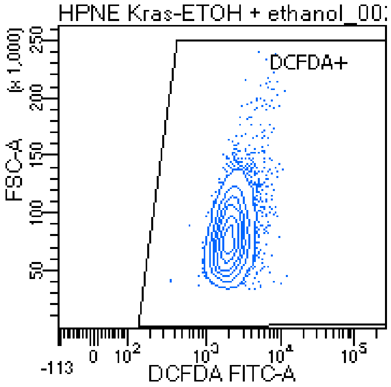


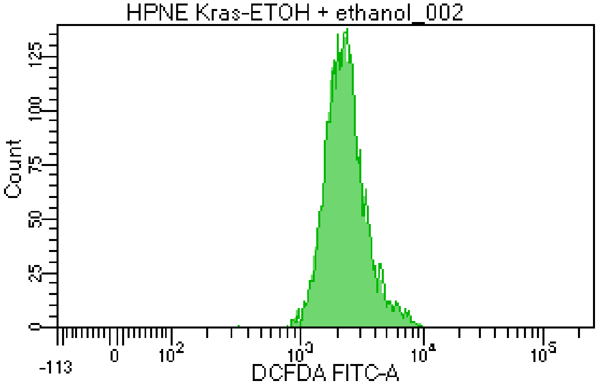


Specimen Name: HPNE Kras Tube Name: ETOH + ethanol_002

| Population | #Events | %Parent | %Total | DCFDA FITC-A Median |
|---|---|---|---|---|
| All Events | 5,035 | #### | 100.0 | 2,164 |
| Gate | 3,590 | 71.3 | 71.3 | 2,316 |
| Single Cells | 2,874 | 80.1 | 57.1 | 2,084 |
| DCFDA+ | 2,874 | 100.0 | 57.1 | 2,084 |

# BD FACSDiva 8.0.1

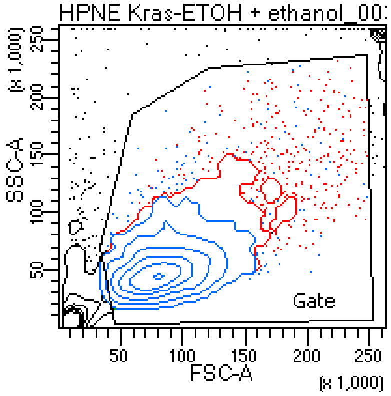


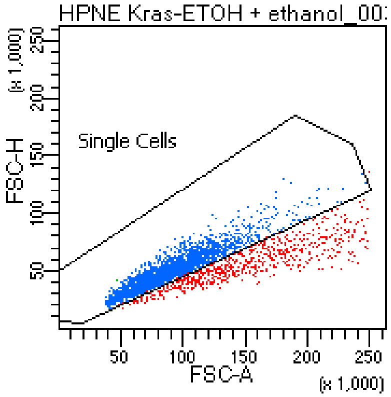


Tube: ETOH + ethanol_003

| Population | #Events | %Parent | %Total |
|---|---|---|---|
| All Events | 4,829 | #### | 100.0 |
| Gate | 3,537 | 73.2 | 73.2 |
| Single Cells | 2,881 | 81.5 | 59.7 |
| DCFDA+ | 2,880 | 100.0 | 59.6 |

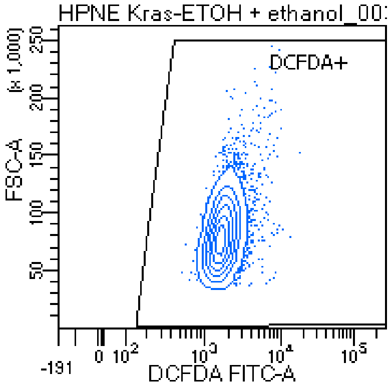


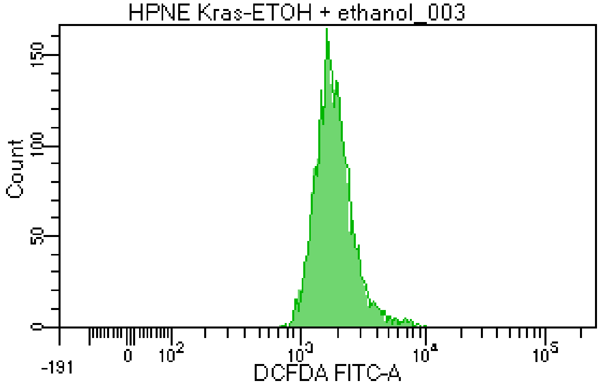


| Specimen Name: | HPNE Kras | Tube Name: | ETOH + ethanol_003 | |
|---|---|---|---|---|
| **Population** | **#Events** | **%Parent** | **%Total** | **DCFDA FITC-A Median** |
| All Events | 4,829 | #### | 100.0 | 1,744 |
| Gate | 3,537 | 73.2 | 73.2 | 1,826 |
| Single Cells | 2,881 | 81.5 | 59.7 | 1,667 |
| DCFDA+ | 2,880 | 100.0 | 59.6 | 1,668 |

# BD FACSDiva 8.0.1

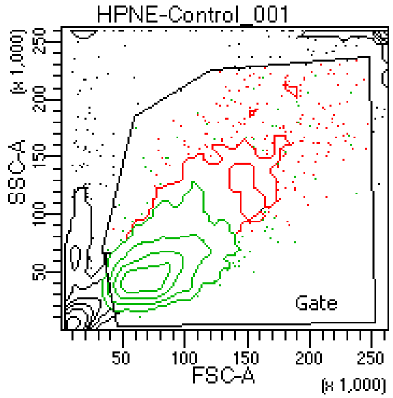


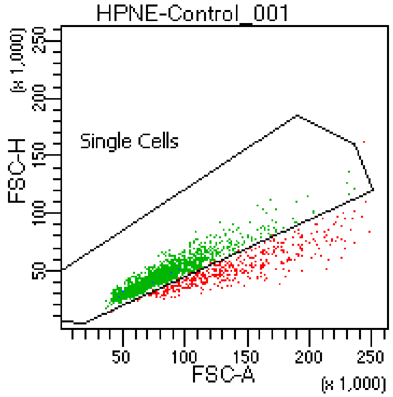


Tube: Control_001

| Population | #Events | %Parent | %Total |
|---|---|---|---|
| All Events | 3,945 | #### | 100.0 |
| Gate | 2,020 | 51.2 | 51.2 |
| Single Cells | 1,569 | 77.7 | 39.8 |
| DCFDA+ | 5 | 0.3 | 0.1 |

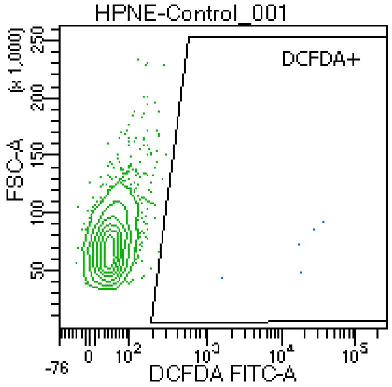


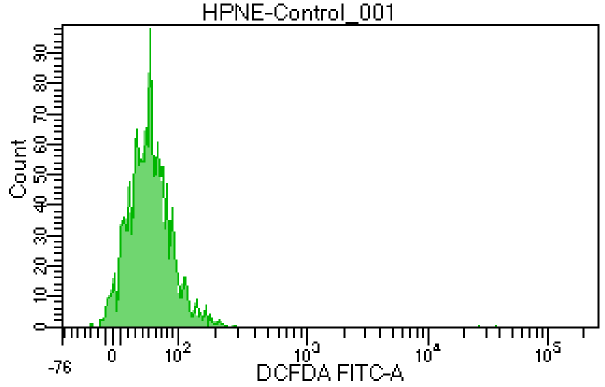


Specimen Name: HPNE Tube Name: Control_001

| Population | #Events | %Parent | %Total | DCFDA FITC-A Median |
|---|---|---|---|---|
| All Events | 3,945 | #### | 100.0 | 37 |
| Gate | 2,020 | 51.2 | 51.2 | 54 |
| Single Cells | 1,569 | 77.7 | 39.8 | 45 |
| DCFDA+ | 5 | 0.3 | 0.1 | 16,875 |

BD FACSDiva 8.0.1

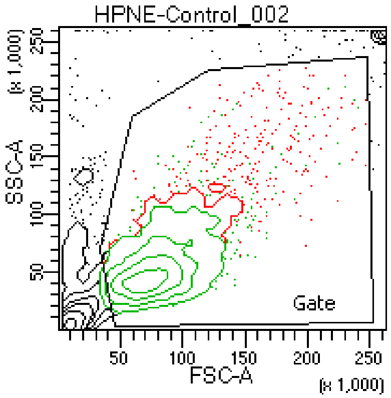


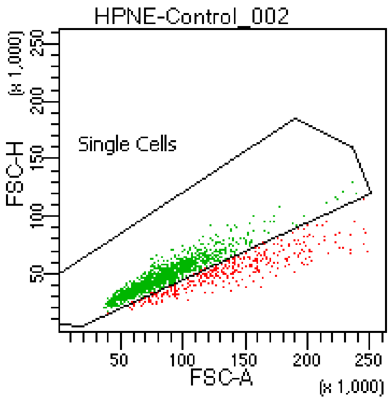


Tube: Control_002

| Population | #Events | %Parent | %Total |
|---|---|---|---|
| All Events | 3,622 | #### | 100.0 |
| Gate | 1,850 | 51.1 | 51.1 |
| Single Cells | 1,496 | 80.9 | 41.3 |
| DCFDA+ | 0 | 0.0 | 0.0 |

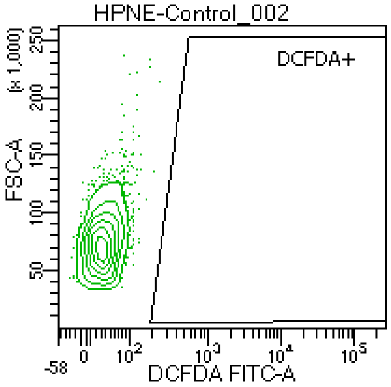


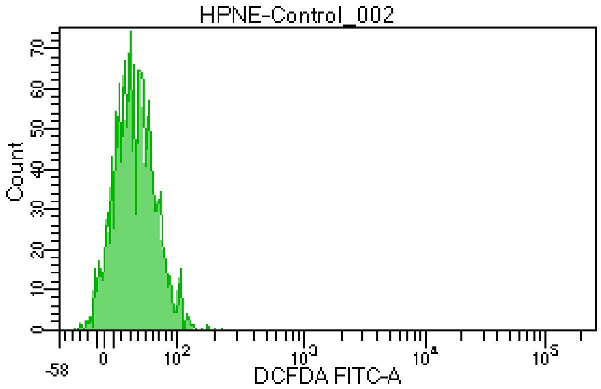


| Specimen Name: | HPNE | Tube Name: | Control_002 | |
|---|---|---|---|---|
| Population | #Events | %Parent | %Total | DCFDA FITC-A Median |
| All Events | 3,622 | #### | 100.0 | 22 |
| Gate | 1,850 | 51.1 | 51.1 | 38 |
| Single Cells | 1,496 | 80.9 | 41.3 | 30 |
| DCFDA+ | 0 | 0.0 | 0.0 | #### |

# BD FACSDiva 8.0.1

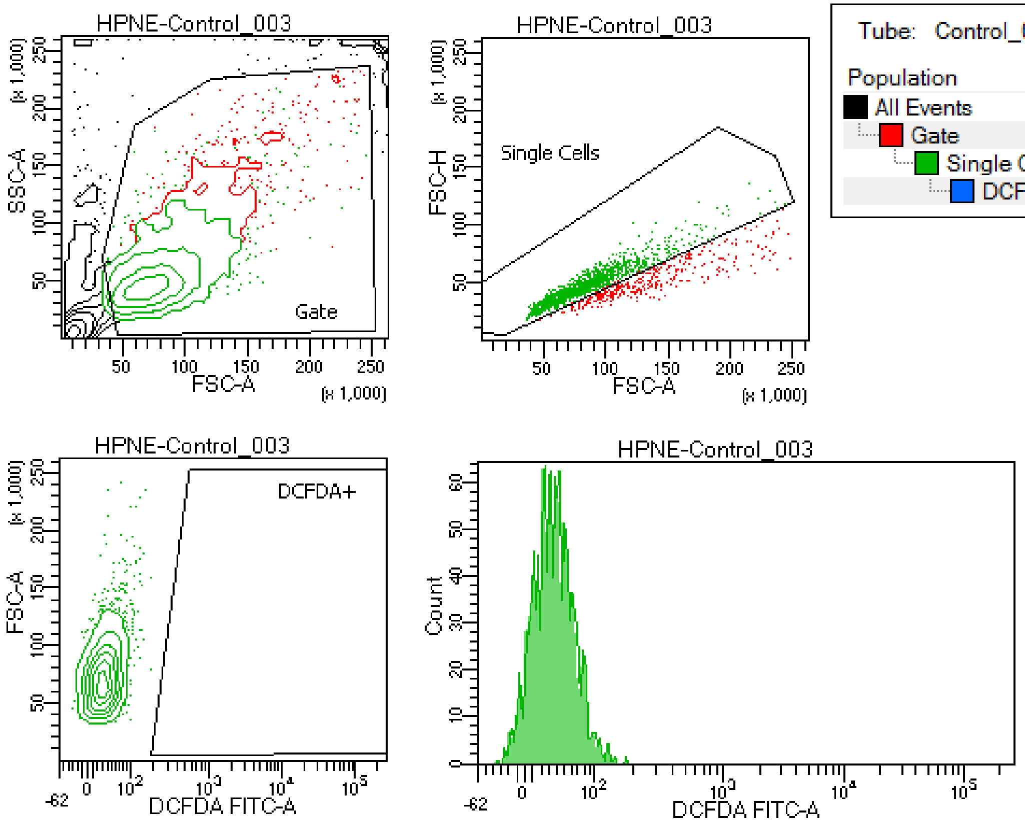


Tube: Control_003

| Population | #Events | %Parent | %Total |
|---|---|---|---|
| All Events | 3,453 | #### | 100.0 |
| Gate | 1,739 | 50.4 | 50.4 |
| Single Cells | 1,361 | 78.3 | 39.4 |
| DCFDA+ | 0 | 0.0 | 0.0 |

Specimen Name: HPNE Tube Name: Control_003

| Population | #Events | %Parent | %Total | DCFDA FITC-A Median |
|---|---|---|---|---|
| All Events | 3,453 | #### | 100.0 | 25 |
| Gate | 1,739 | 50.4 | 50.4 | 38 |
| Single Cells | 1,361 | 78.3 | 39.4 | 30 |
| DCFDA+ | 0 | 0.0 | 0.0 | #### |

BD FACSDiva 8.0.1

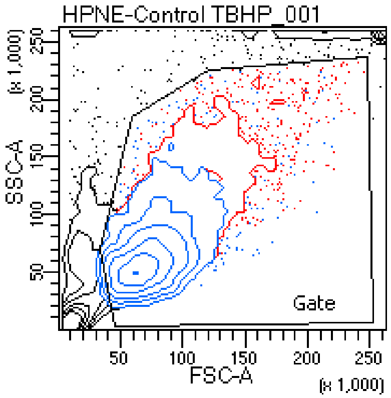


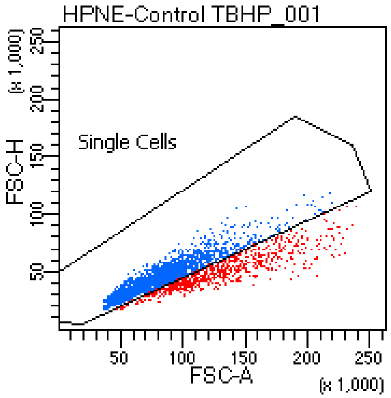


Tube: Control TBHP_001

| Population | #Events | %Parent | %Total |
|---|---|---|---|
| All Events | 5,292 | #### | 100.0 |
| Gate | 3,481 | 65.8 | 65.8 |
| Single Cells | 2,714 | 78.0 | 51.3 |
| DCFDA+ | 2,714 | 100.0 | 51.3 |

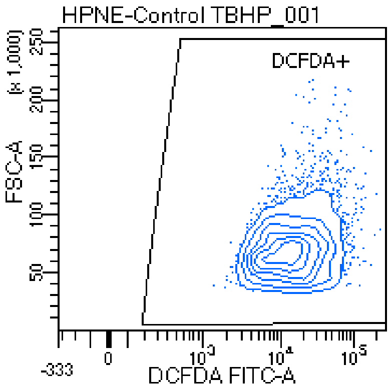


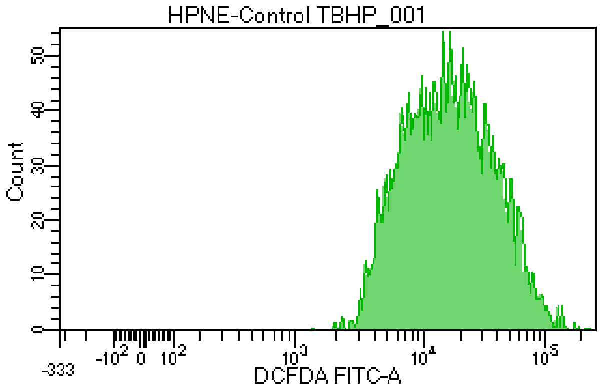


Specimen Name: HPNE Tube Name: Control TBHP_001

| Population | #Events | %Parent | %Total | DCFDA FITC-A Median |
|---|---|---|---|---|
| All Events | 5,292 | #### | 100.0 | 15,872 |
| Gate | 3,481 | 65.8 | 65.8 | 18,616 |
| Single Cells | 2,714 | 78.0 | 51.3 | 14,644 |
| DCFDA+ | 2,714 | 100.0 | 51.3 | 14,644 |

# BD FACSDiva 8.0.1

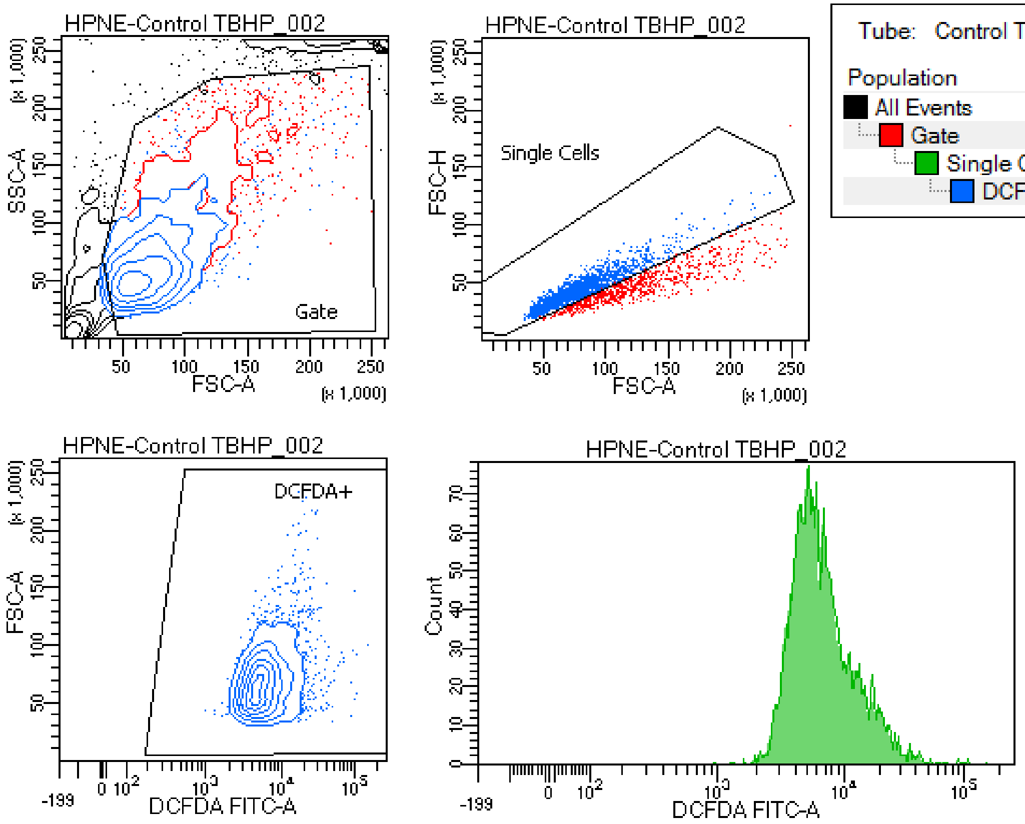


Tube: Control TBHP_002

| Population | #Events | %Parent | %Total |
|---|---|---|---|
| All Events | 4,958 | #### | 100.0 |
| Gate | 2,923 | 59.0 | 59.0 |
| Single Cells | 2,141 | 73.2 | 43.2 |
| DCFDA+ | 2,141 | 100.0 | 43.2 |

Specimen Name: HPNE    Tube Name: Control TBHP_002

| Population | #Events | %Parent | %Total | DCFDA FITC-A Median |
|---|---|---|---|---|
| All Events | 4,958 | #### | 100.0 | 6,842 |
| Gate | 2,923 | 59.0 | 59.0 | 7,480 |
| Single Cells | 2,141 | 73.2 | 43.2 | 5,666 |
| DCFDA+ | 2,141 | 100.0 | 43.2 | 5,666 |

# BD FACSDiva 8.0.1

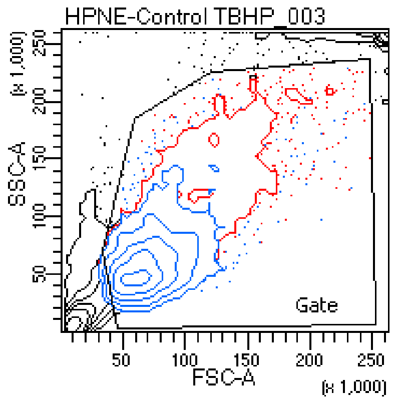


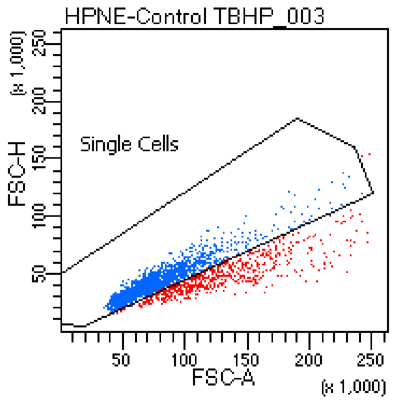


Tube: Control TBHP_003

| Population | #Events | %Parent | %Total |
|---|---|---|---|
| All Events | 4,194 | #### | 100.0 |
| Gate | 2,428 | 57.9 | 57.9 |
| Single Cells | 1,777 | 73.2 | 42.4 |
| DCFDA+ | 1,776 | 99.9 | 42.3 |

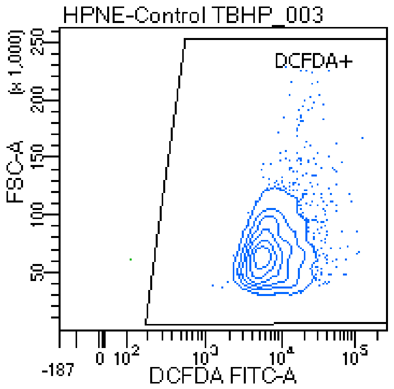


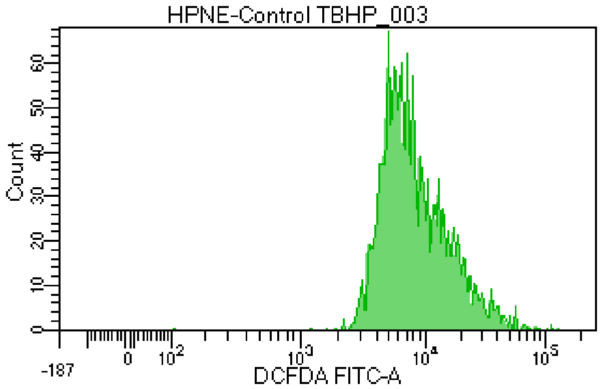


Specimen Name: HPNE    Tube Name: Control TBHP_003

| Population | #Events | %Parent | %Total | DCFDA FITC-A Median |
|---|---|---|---|---|
| All Events | 4,194 | #### | 100.0 | 8,006 |
| Gate | 2,428 | 57.9 | 57.9 | 9,493 |
| Single Cells | 1,777 | 73.2 | 42.4 | 6,799 |
| DCFDA+ | 1,776 | 99.9 | 42.3 | 6,802 |

# BD FACSDiva 8.0.1

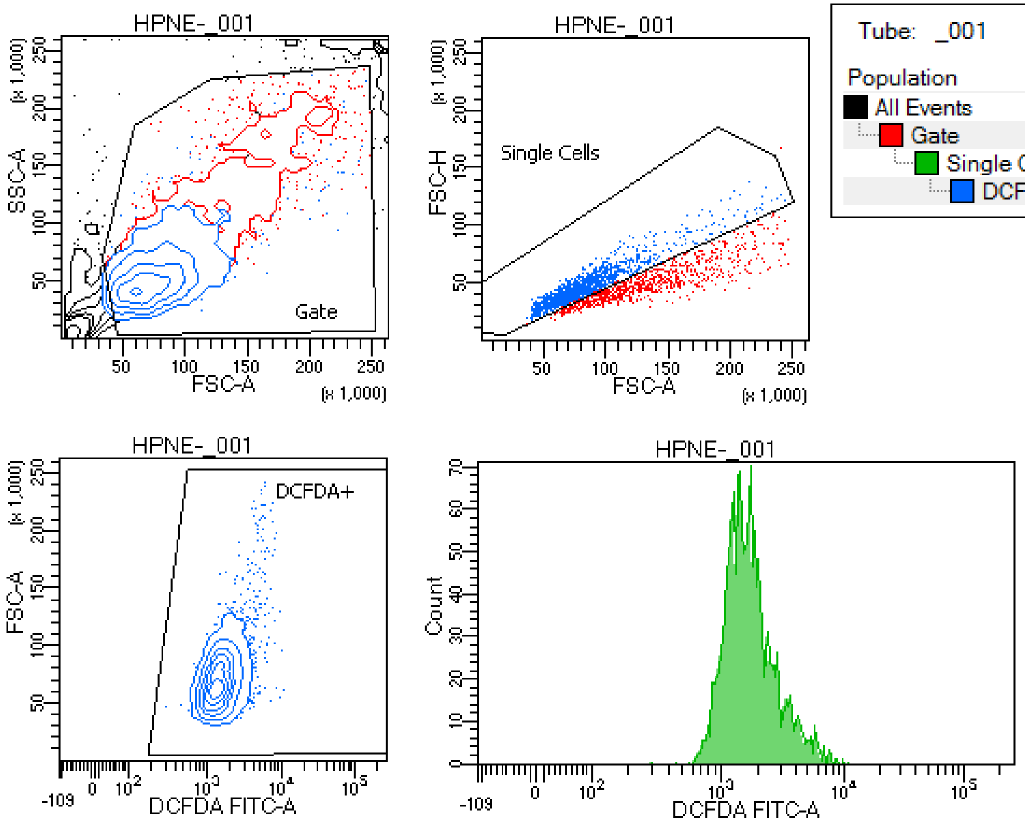


Tube: _001

| Population | #Events | %Parent | %Total |
|---|---|---|---|
| All Events | 4,246 | #### | 100.0 |
| Gate | 2,383 | 56.1 | 56.1 |
| Single Cells | 1,560 | 65.5 | 36.7 |
| DCFDA+ | 1,560 | 100.0 | 36.7 |

Specimen Name: HPNE Tube Name: _001

| Population | #Events | %Parent | %Total | DCFDA FITC-A Median |
|---|---|---|---|---|
| All Events | 4,246 | #### | 100.0 | 1,617 |
| Gate | 2,383 | 56.1 | 56.1 | 2,067 |
| Single Cells | 1,560 | 65.5 | 36.7 | 1,495 |
| DCFDA+ | 1,560 | 100.0 | 36.7 | 1,495 |

# BD FACSDiva 8.0.1

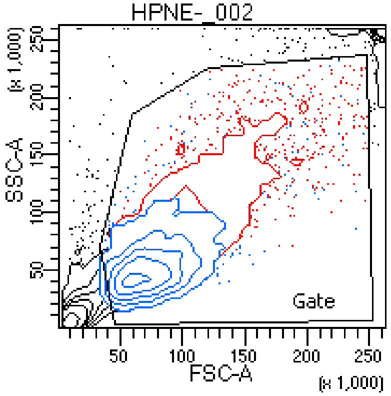


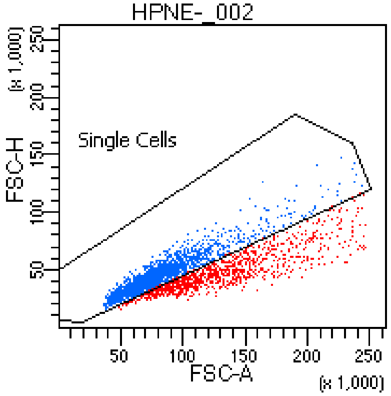


Tube: _002

| Population | #Events | %Parent | %Total |
|---|---|---|---|
| All Events | 4,972 | #### | 100.0 |
| Gate | 2,954 | 59.4 | 59.4 |
| Single Cells | 1,924 | 65.1 | 38.7 |
| DCFDA+ | 1,924 | 100.0 | 38.7 |

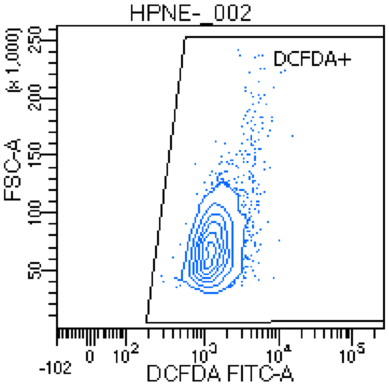


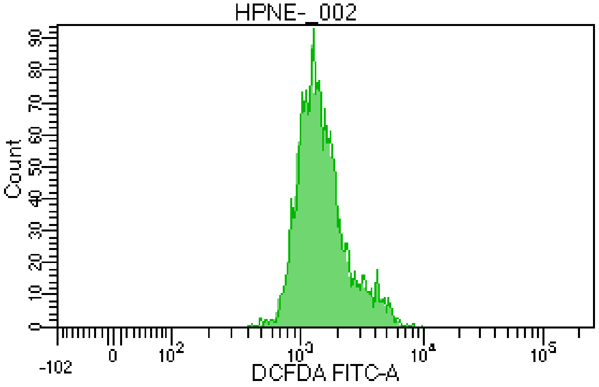


Specimen Name: HPNE Tube Name: _002

| Population | #Events | %Parent | %Total | DCFDA FITC-A Median |
|---|---|---|---|---|
| All Events | 4,972 | #### | 100.0 | 1,428 |
| Gate | 2,954 | 59.4 | 59.4 | 1,765 |
| Single Cells | 1,924 | 65.1 | 38.7 | 1,290 |
| DCFDA+ | 1,924 | 100.0 | 38.7 | 1,290 |

# BD FACSDiva 8.0.1

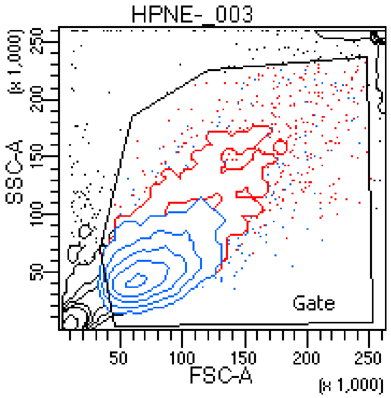


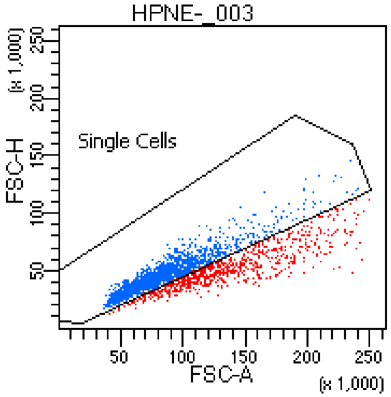


Tube: _003

| Population | #Events | %Parent | %Total |
|---|---|---|---|
| All Events | 4,564 | #### | 100.0 |
| Gate | 2,614 | 57.3 | 57.3 |
| Single Cells | 1,863 | 71.3 | 40.8 |
| DCFDA+ | 1,863 | 100.0 | 40.8 |

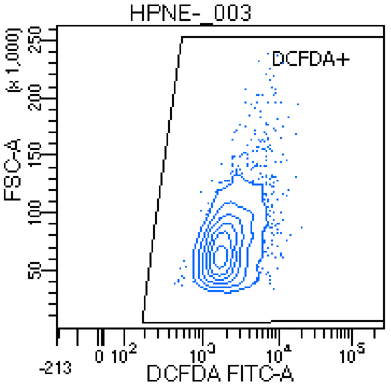


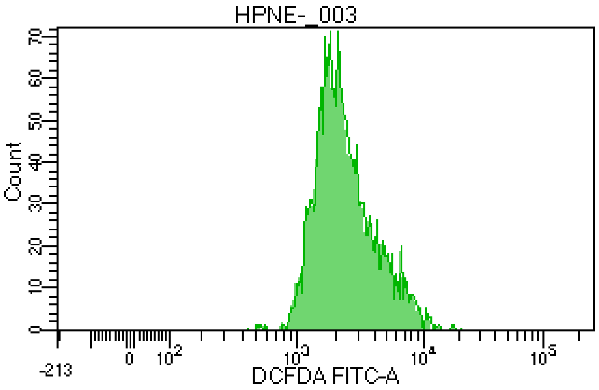


Specimen Name: HPNE    Tube Name: _003

| Population | #Events | %Parent | %Total | DCFDA FITC-A Median |
|---|---|---|---|---|
| All Events | 4,564 | #### | 100.0 | 2,089 |
| Gate | 2,614 | 57.3 | 57.3 | 2,685 |
| Single Cells | 1,863 | 71.3 | 40.8 | 1,997 |
| DCFDA+ | 1,863 | 100.0 | 40.8 | 1,997 |

BD FACSDiva 8.0.1

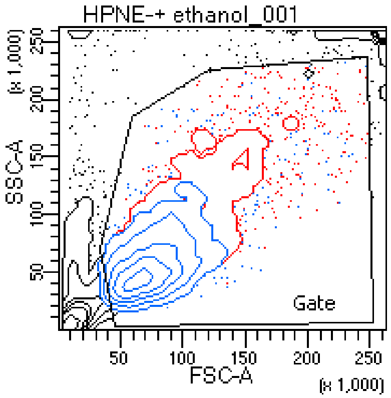


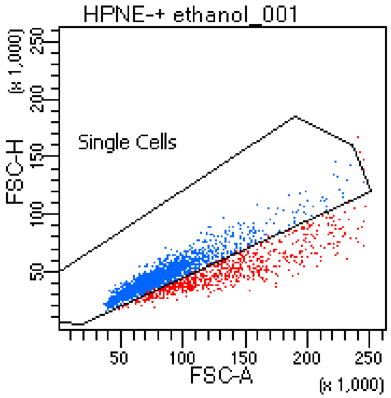


| Tube: + ethanol_001 | | | |
|---|---|---|---|
| Population | #Events | %Parent | %Total |
| All Events | 4,766 | #### | 100.0 |
| Gate | 2,739 | 57.5 | 57.5 |
| Single Cells | 1,996 | 72.9 | 41.9 |
| DCFDA+ | 1,996 | 100.0 | 41.9 |

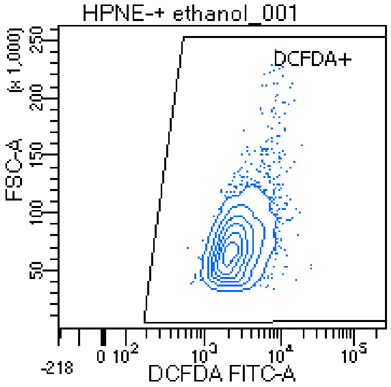


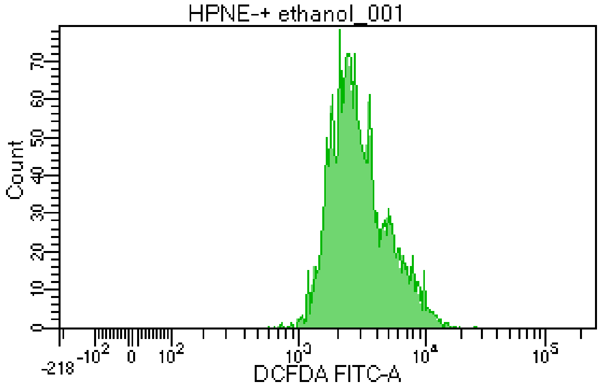


Specimen Name: HPNE Tube Name: + ethanol_001

| Population | #Events | %Parent | %Total | DCFDA FITC-A Median |
|---|---|---|---|---|
| All Events | 4,766 | #### | 100.0 | 2,535 |
| Gate | 2,739 | 57.5 | 57.5 | 3,269 |
| Single Cells | 1,996 | 72.9 | 41.9 | 2,512 |
| DCFDA+ | 1,996 | 100.0 | 41.9 | 2,512 |

BD FACSDiva 8.0.1

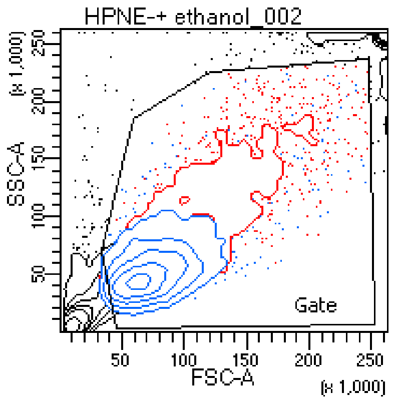


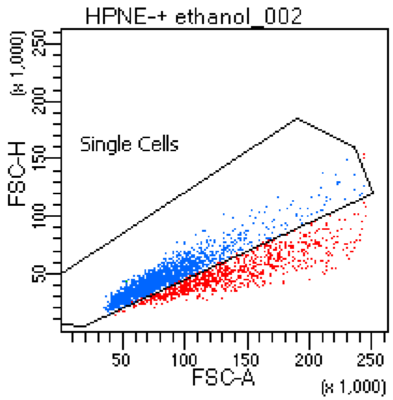


| Tube: + ethanol_002 | | | |
|---|---|---|---|
| Population | #Events | %Parent | %Total |
| All Events | 5,167 | #### | 100.0 |
| Gate | 3,064 | 59.3 | 59.3 |
| Single Cells | 2,150 | 70.2 | 41.6 |
| DCFDA+ | 2,149 | 100.0 | 41.6 |

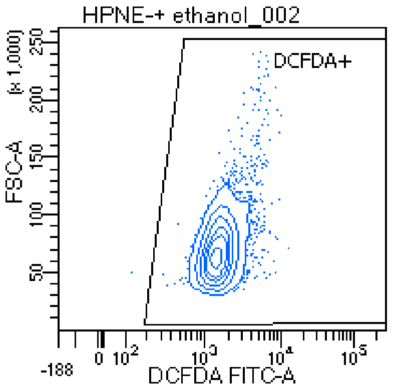


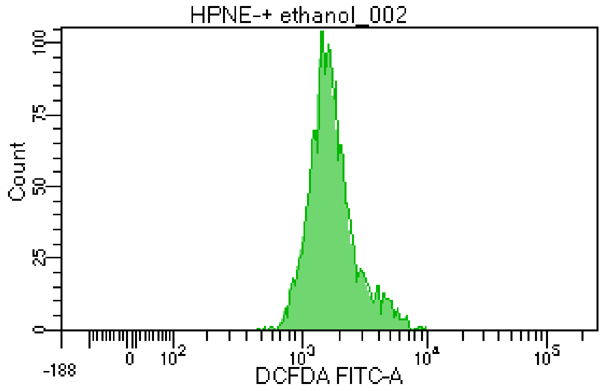


Specimen Name: HPNE Tube Name: + ethanol_002

| Population | #Events | %Parent | %Total | DCFDA FITC-A Median |
|---|---|---|---|---|
| All Events | 5,167 | #### | 100.0 | 1,606 |
| Gate | 3,064 | 59.3 | 59.3 | 1,879 |
| Single Cells | 2,150 | 70.2 | 41.6 | 1,521 |
| DCFDA+ | 2,149 | 100.0 | 41.6 | 1,522 |

# BD FACSDiva 8.0.1

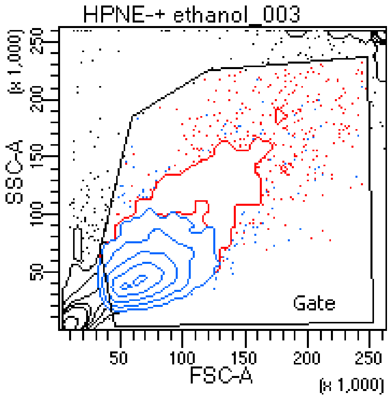


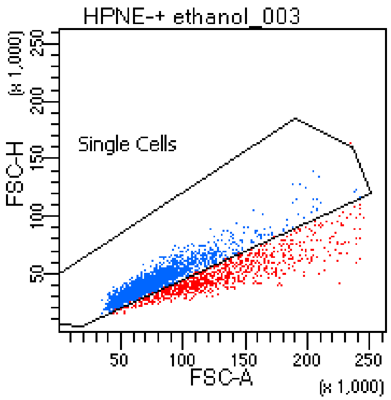


Tube: + ethanol_003

| Population | #Events | %Parent | %Total |
|---|---|---|---|
| All Events | 4,696 | #### | 100.0 |
| Gate | 2,615 | 55.7 | 55.7 |
| Single Cells | 1,797 | 68.7 | 38.3 |
| DCFDA+ | 1,797 | 100.0 | 38.3 |

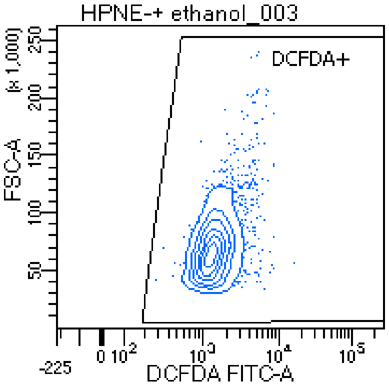


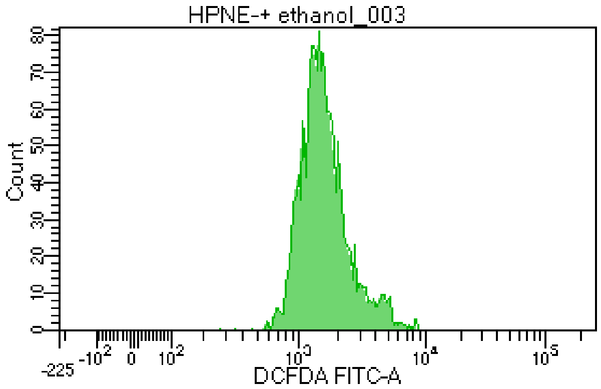


Specimen Name: HPNE    Tube Name: + ethanol_003

| Population | #Events | %Parent | %Total | DCFDA FITC-A Median |
|---|---|---|---|---|
| All Events | 4,696 | #### | 100.0 | 1,470 |
| Gate | 2,615 | 55.7 | 55.7 | 1,749 |
| Single Cells | 1,797 | 68.7 | 38.3 | 1,379 |
| DCFDA+ | 1,797 | 100.0 | 38.3 | 1,379 |

# BD FACSDiva 8.0.1

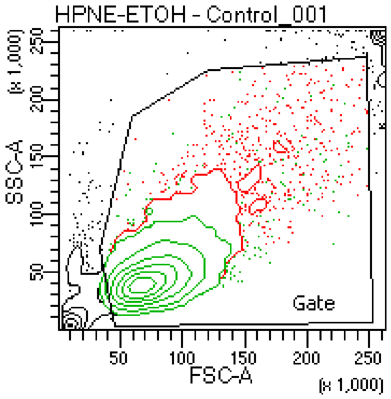


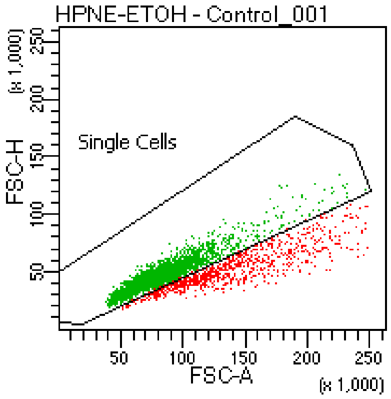


Tube: ETOH - Control_001

| Population | #Events | %Parent | %Total |
|---|---|---|---|
| All Events | 5,963 | #### | 100.0 |
| Gate | 4,835 | 81.1 | 81.1 |
| Single Cells | 3,987 | 82.5 | 66.9 |
| DCFDA+ | 0 | 0.0 | 0.0 |

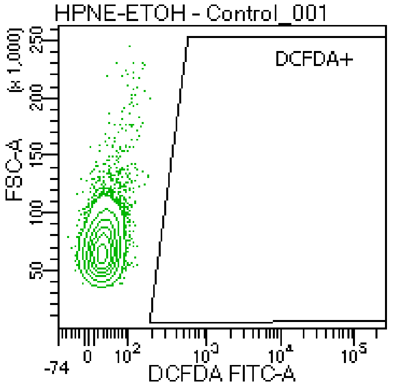


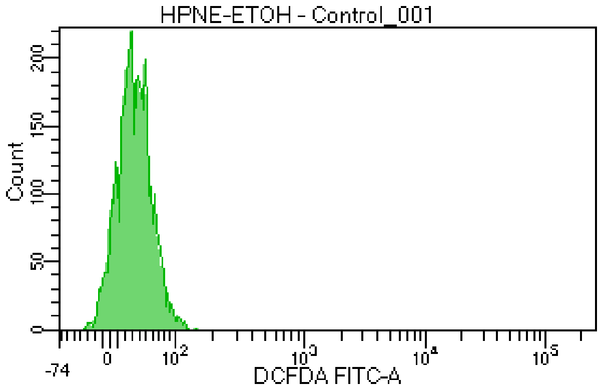


Specimen Name: HPNE Tube Name: ETOH - Control_001

| Population | #Events | %Parent | %Total | DCFDA FITC-A Median |
|---|---|---|---|---|
| All Events | 5,963 | #### | 100.0 | 34 |
| Gate | 4,835 | 81.1 | 81.1 | 33 |
| Single Cells | 3,987 | 82.5 | 66.9 | 28 |
| DCFDA+ | 0 | 0.0 | 0.0 | #### |

# BD FACSDiva 8.0.1

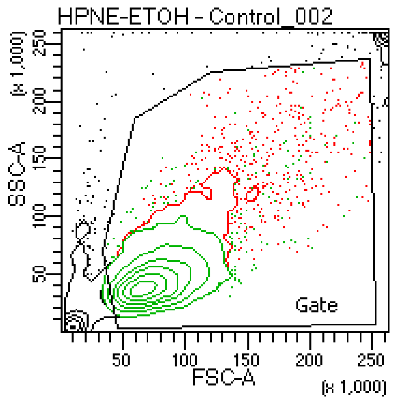


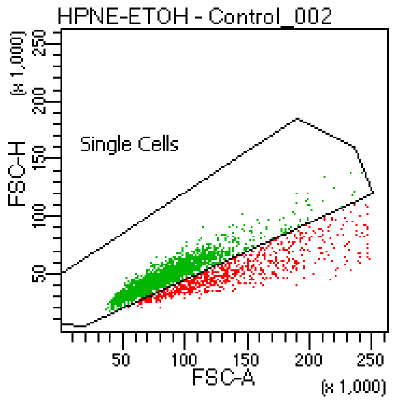


Tube: ETOH - Control_002

| Population | #Events | %Parent | %Total |
|---|---|---|---|
| All Events | 5,591 | #### | 100.0 |
| Gate | 4,438 | 79.4 | 79.4 |
| Single Cells | 3,674 | 82.8 | 65.7 |
| DCFDA+ | 0 | 0.0 | 0.0 |

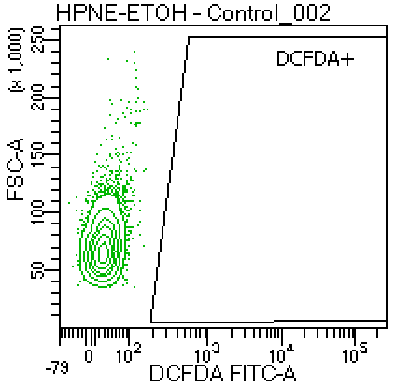


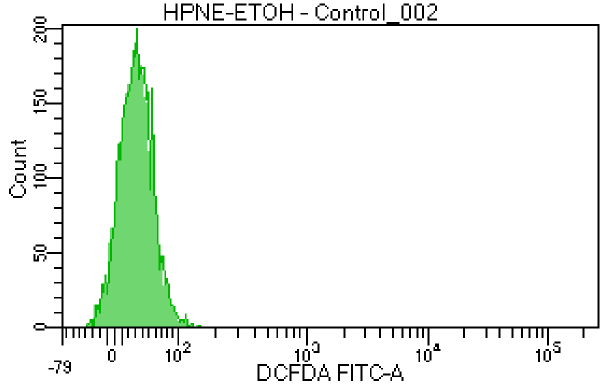


| Specimen Name: | HPNE | Tube Name: | ETOH - Control_002 | |
|---|---|---|---|---|
| **Population** | **#Events** | **%Parent** | **%Total** | **DCFDA FITC-A Median** |
| All Events | 5,591 | #### | 100.0 | 32 |
| Gate | 4,438 | 79.4 | 79.4 | 31 |
| Single Cells | 3,674 | 82.8 | 65.7 | 26 |
| DCFDA+ | 0 | 0.0 | 0.0 | #### |

## BD FACSDiva 8.0.1

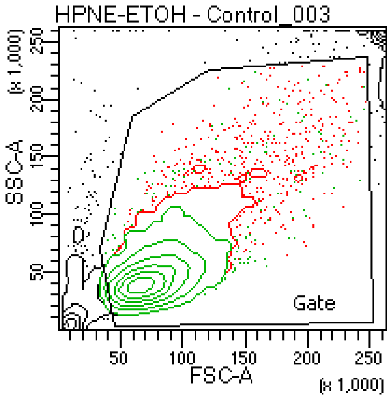


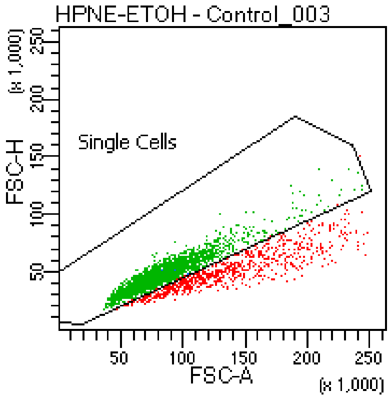


Tube: ETOH - Control_003

| Population | #Events | %Parent | %Total |
|---|---|---|---|
| All Events | 5,628 | #### | 100.0 |
| Gate | 4,502 | 80.0 | 80.0 |
| Single Cells | 3,733 | 82.9 | 66.3 |
| DCFDA+ | 3 | 0.1 | 0.1 |

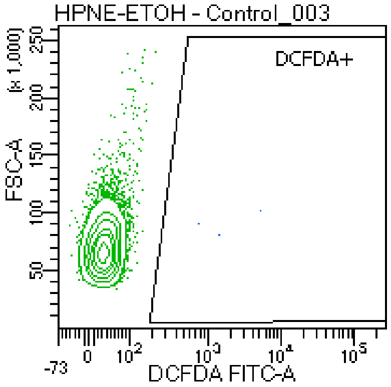


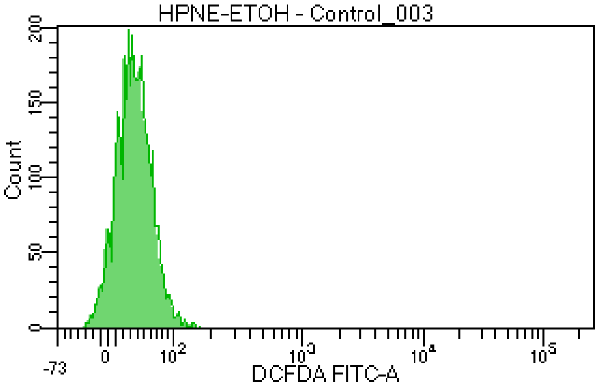


Specimen Name: HPNE    Tube Name: ETOH - Control_003

| Population | #Events | %Parent | %Total | DCFDA FITC-A Median |
|---|---|---|---|---|
| All Events | 5,628 | #### | 100.0 | 37 |
| Gate | 4,502 | 80.0 | 80.0 | 37 |
| Single Cells | 3,733 | 82.9 | 66.3 | 30 |
| DCFDA+ | 3 | 0.1 | 0.1 | 1,309 |

BD FACSDiva 8.0.1

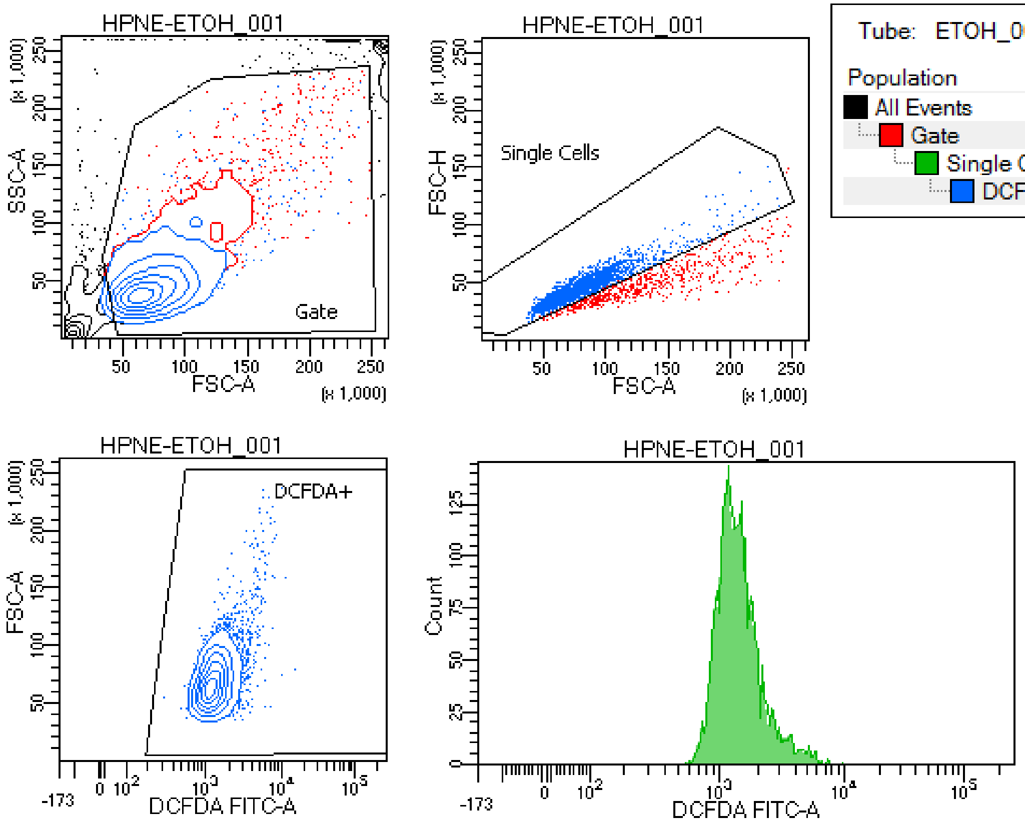


Tube: ETOH_001

| Population | #Events | %Parent | %Total |
|---|---|---|---|
| All Events | 4,602 | #### | 100.0 |
| Gate | 3,523 | 76.6 | 76.6 |
| Single Cells | 2,863 | 81.3 | 62.2 |
| DCFDA+ | 2,863 | 100.0 | 62.2 |

Specimen Name: HPNE Tube Name: ETOH_001

| Population | #Events | %Parent | %Total | DCFDA FITC-A Median |
|---|---|---|---|---|
| All Events | 4,602 | #### | 100.0 | 1,420 |
| Gate | 3,523 | 76.6 | 76.6 | 1,402 |
| Single Cells | 2,863 | 81.3 | 62.2 | 1,263 |
| DCFDA+ | 2,863 | 100.0 | 62.2 | 1,263 |

# BD FACSDiva 8.0.1

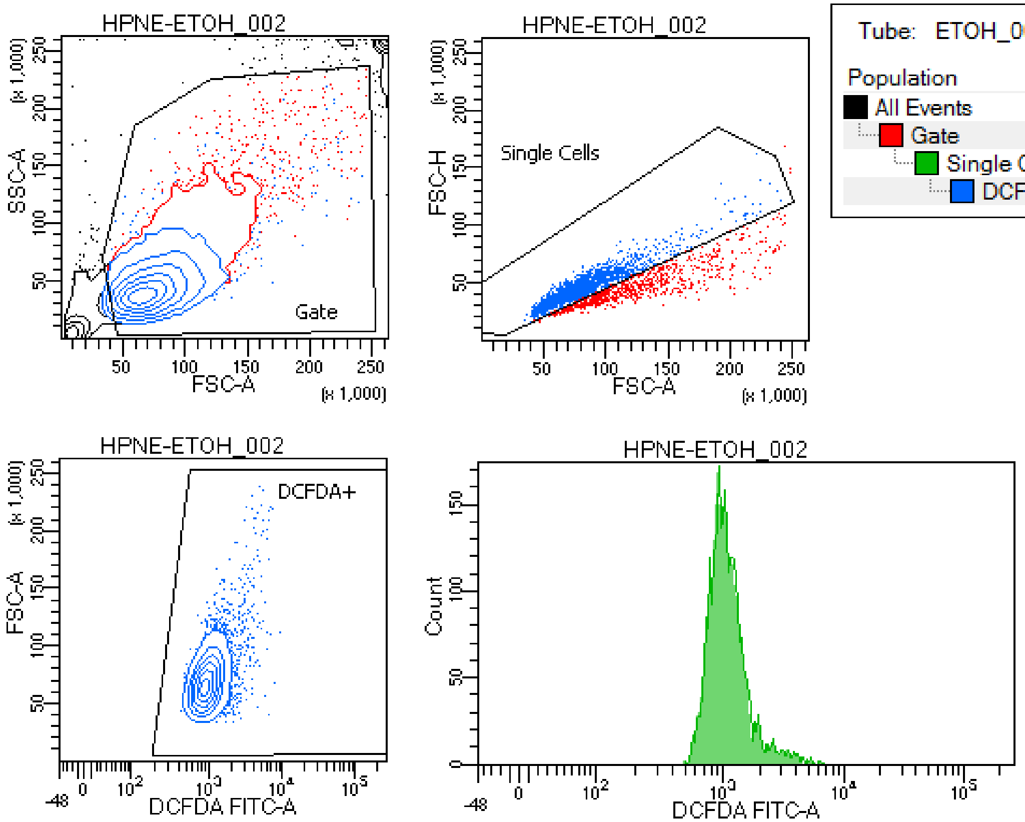


Tube: ETOH_002

| Population | #Events | %Parent | %Total |
|---|---|---|---|
| All Events | 4,501 | #### | 100.0 |
| Gate | 3,621 | 80.4 | 80.4 |
| Single Cells | 2,832 | 78.2 | 62.9 |
| DCFDA+ | 2,832 | 100.0 | 62.9 |

Specimen Name: HPNE    Tube Name: ETOH_002

| Population | #Events | %Parent | %Total | DCFDA FITC-A Median |
|---|---|---|---|---|
| All Events | 4,501 | #### | 100.0 | 1,096 |
| Gate | 3,621 | 80.4 | 80.4 | 1,087 |
| Single Cells | 2,832 | 78.2 | 62.9 | 966 |
| DCFDA+ | 2,832 | 100.0 | 62.9 | 966 |

# BD FACSDiva 8.0.1

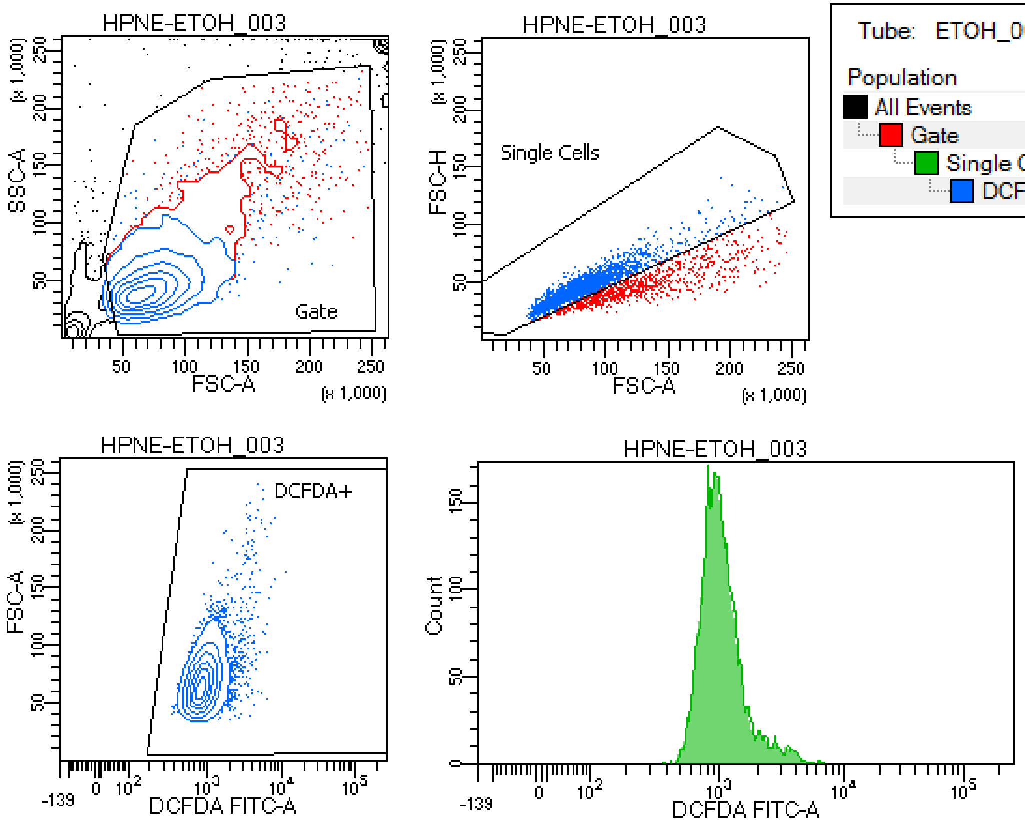


Tube: ETOH_003

| Population | #Events | %Parent | %Total |
|---|---|---|---|
| All Events | 5,181 | #### | 100.0 |
| Gate | 4,180 | 80.7 | 80.7 |
| Single Cells | 3,273 | 78.3 | 63.2 |
| DCFDA+ | 3,273 | 100.0 | 63.2 |

Specimen Name: HPNE    Tube Name: ETOH_003

| Population | #Events | %Parent | %Total | DCFDA FITC-A Median |
|---|---|---|---|---|
| All Events | 5,181 | #### | 100.0 | 1,034 |
| Gate | 4,180 | 80.7 | 80.7 | 1,029 |
| Single Cells | 3,273 | 78.3 | 63.2 | 911 |
| DCFDA+ | 3,273 | 100.0 | 63.2 | 911 |

# BD FACSDiva 8.0.1

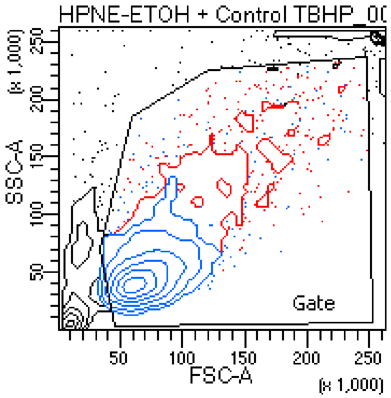


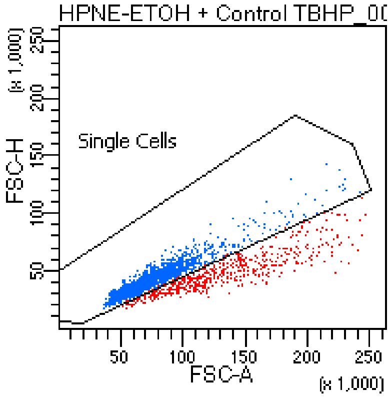


Tube: ETOH + Control TBHP_001

| Population | #Events | %Parent | %Total |
|---|---|---|---|
| All Events | 3,853 | #### | 100.0 |
| Gate | 2,766 | 71.8 | 71.8 |
| Single Cells | 2,183 | 78.9 | 56.7 |
| DCFDA+ | 2,182 | 100.0 | 56.6 |

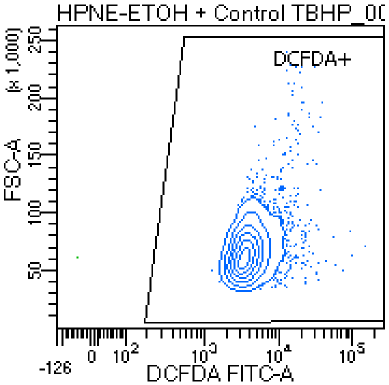


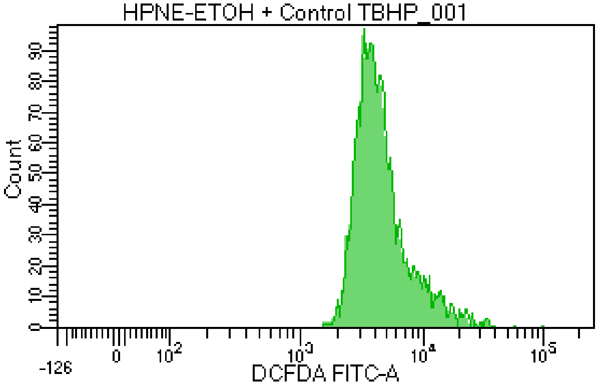


Specimen Name: HPNE Tube Name: ETOH + Control TBHP_001

| Population | #Events | %Parent | %Total | DCFDA FITC-A Median |
|---|---|---|---|---|
| All Events | 3,853 | #### | 100.0 | 4,895 |
| Gate | 2,766 | 71.8 | 71.8 | 4,397 |
| Single Cells | 2,183 | 78.9 | 56.7 | 3,810 |
| DCFDA+ | 2,182 | 100.0 | 56.6 | 3,810 |

# BD FACSDiva 8.0.1

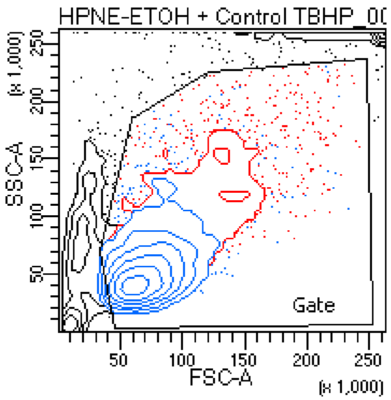


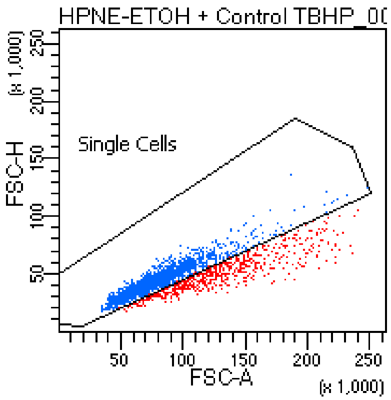


Tube: ETOH + Control TBHP_002

| Population | #Events | %Parent | %Total |
|---|---|---|---|
| All Events | 4,988 | #### | 100.0 |
| Gate | 3,580 | 71.8 | 71.8 |
| Single Cells | 2,929 | 81.8 | 58.7 |
| DCFDA+ | 2,929 | 100.0 | 58.7 |

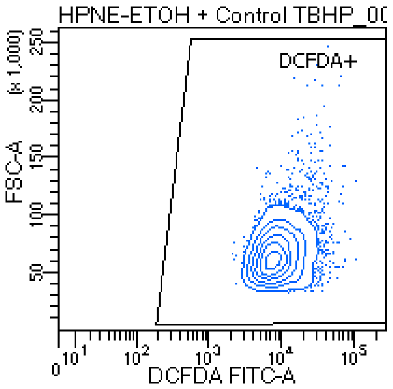


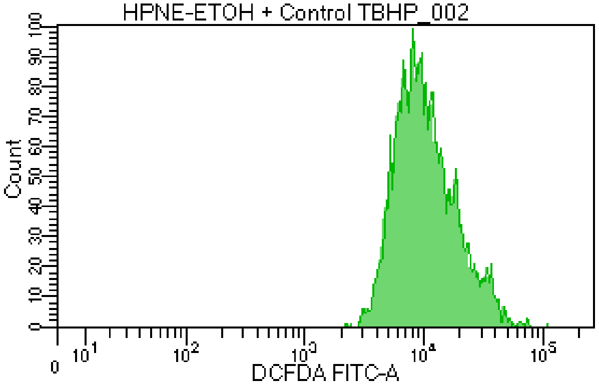


Specimen Name: HPNE Tube Name: ETOH + Control TBHP_002

| Population | #Events | %Parent | %Total | DCFDA FITC-A Median |
|---|---|---|---|---|
| All Events | 4,988 | #### | 100.0 | 11,222 |
| Gate | 3,580 | 71.8 | 71.8 | 10,583 |
| Single Cells | 2,929 | 81.8 | 58.7 | 8,956 |
| DCFDA+ | 2,929 | 100.0 | 58.7 | 8,956 |

BD FACSDiva 8.0.1

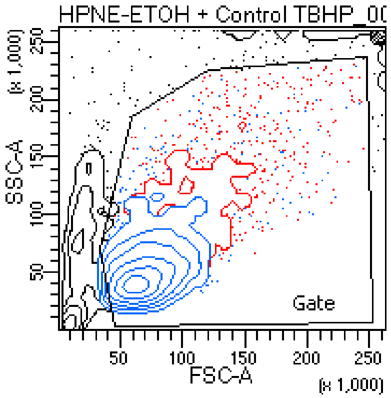


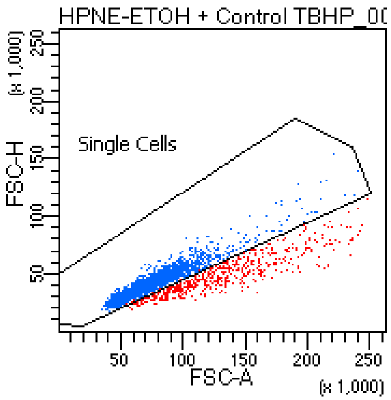


| Tube: ETOH + Control TBHP_003 | | | |
|---|---|---|---|
| Population | #Events | %Parent | %Total |
| All Events | 4,407 | #### | 100.0 |
| Gate | 3,174 | 72.0 | 72.0 |
| Single Cells | 2,577 | 81.2 | 58.5 |
| DCFDA+ | 2,577 | 100.0 | 58.5 |

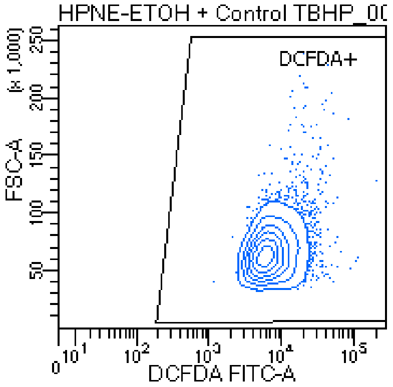


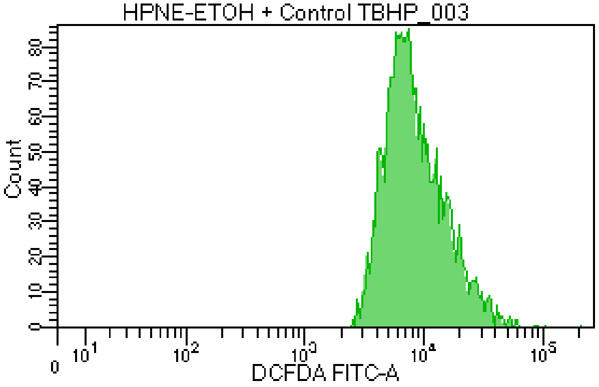


Specimen Name: HPNE Tube Name: ETOH + Control TBHP_003

| Population | #Events | %Parent | %Total | DCFDA FITC-A Median |
|---|---|---|---|---|
| All Events | 4,407 | #### | 100.0 | 9,735 |
| Gate | 3,174 | 72.0 | 72.0 | 8,515 |
| Single Cells | 2,577 | 81.2 | 58.5 | 7,122 |
| DCFDA+ | 2,577 | 100.0 | 58.5 | 7,122 |

# BD FACSDiva 8.0.1

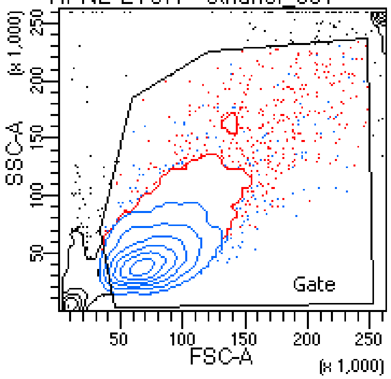


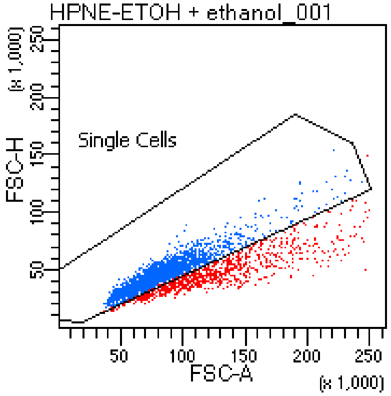


Tube: ETOH + ethanol_001

| Population | #Events | %Parent | %Total |
|---|---|---|---|
| All Events | 5,118 | #### | 100.0 |
| Gate | 4,113 | 80.4 | 80.4 |
| Single Cells | 3,244 | 78.9 | 63.4 |
| DCFDA+ | 3,244 | 100.0 | 63.4 |

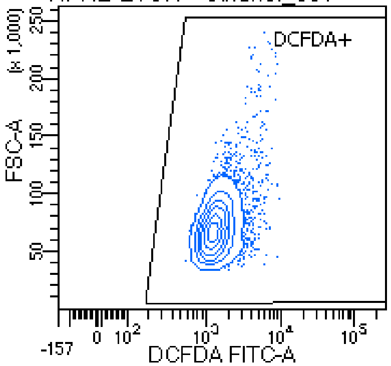


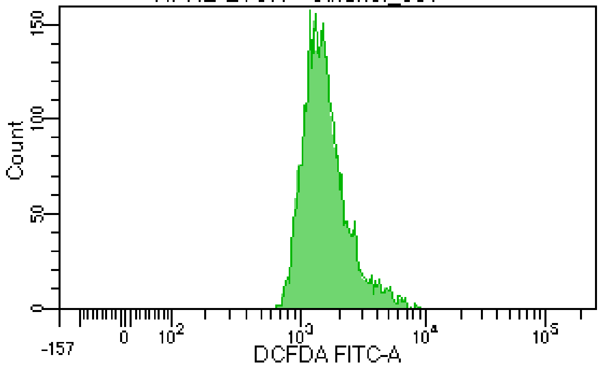


| Specimen Name: | HPNE | Tube Name: | ETOH + ethanol_001 | |
|---|---|---|---|---|
| **Population** | **#Events** | **%Parent** | **%Total** | **DCFDA FITC-A Median** |
| All Events | 5,118 | #### | 100.0 | 1,550 |
| Gate | 4,113 | 80.4 | 80.4 | 1,534 |
| Single Cells | 3,244 | 78.9 | 63.4 | 1,366 |
| DCFDA+ | 3,244 | 100.0 | 63.4 | 1,366 |

# BD FACSDiva 8.0.1

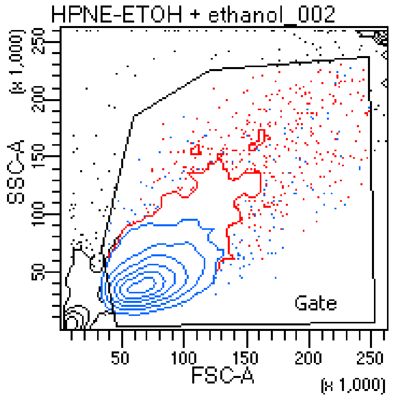


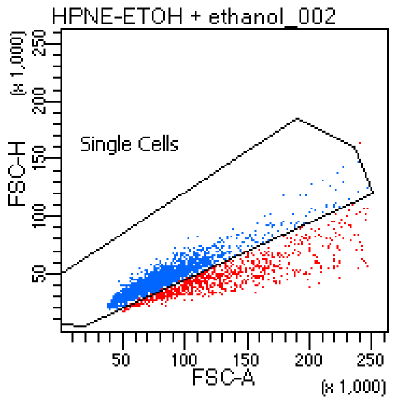


Tube: ETOH + ethanol_002

| Population | #Events | %Parent | %Total |
|---|---|---|---|
| All Events | 4,941 | #### | 100.0 |
| Gate | 3,921 | 79.4 | 79.4 |
| Single Cells | 3,114 | 79.4 | 63.0 |
| DCFDA+ | 3,114 | 100.0 | 63.0 |

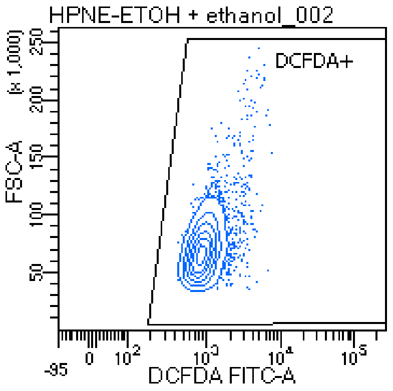


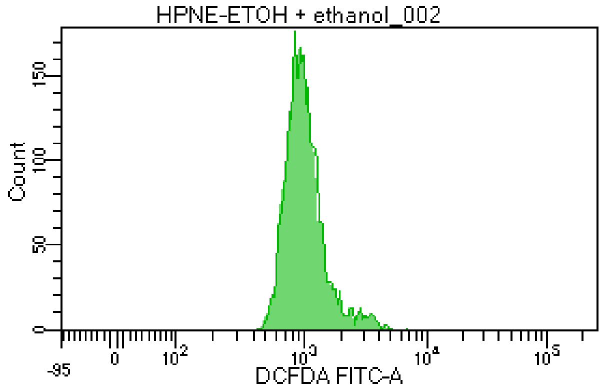


Specimen Name: HPNE Tube Name: ETOH + ethanol_002

| Population | #Events | %Parent | %Total | DCFDA FITC-A Median |
|---|---|---|---|---|
| All Events | 4,941 | #### | 100.0 | 997 |
| Gate | 3,921 | 79.4 | 79.4 | 990 |
| Single Cells | 3,114 | 79.4 | 63.0 | 891 |
| DCFDA+ | 3,114 | 100.0 | 63.0 | 891 |

# BD FACSDiva 8.0.1

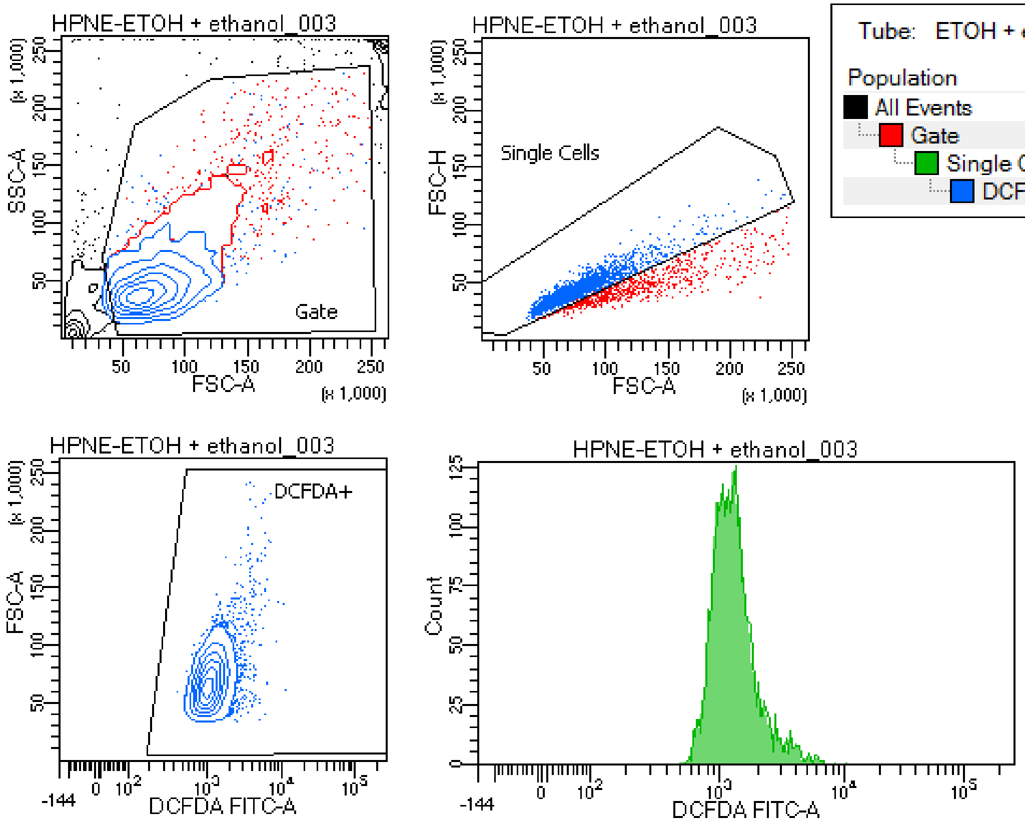


Tube: ETOH + ethanol_003

| Population | #Events | %Parent | %Total |
|---|---|---|---|
| All Events | 4,284 | #### | 100.0 |
| Gate | 3,297 | 77.0 | 77.0 |
| Single Cells | 2,603 | 79.0 | 60.8 |
| DCFDA+ | 2,603 | 100.0 | 60.8 |

Specimen Name: HPNE Tube Name: ETOH + ethanol_003

| Population | #Events | %Parent | %Total | DCFDA FITC-A Median |
|---|---|---|---|---|
| All Events | 4,284 | #### | 100.0 | 1,286 |
| Gate | 3,297 | 77.0 | 77.0 | 1,279 |
| Single Cells | 2,603 | 79.0 | 60.8 | 1,144 |
| DCFDA+ | 2,603 | 100.0 | 60.8 | 1,144 |
