# Supplementary material for: Combined Alcohol Exposure and KRAS Mutation in Human Pancreatic Ductal Epithelial Cells Induces Proliferation and Alters Subtype Signatures Determined by Multi-Omics Analysis
Source: Cancers (Basel). 2022 Apr 13;14(8):1968. doi: 10.3390/cancers14081968 (PMC9027648; doi:10.3390/cancers14081968)

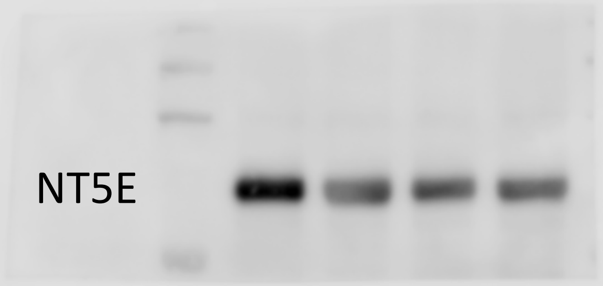
NT5E

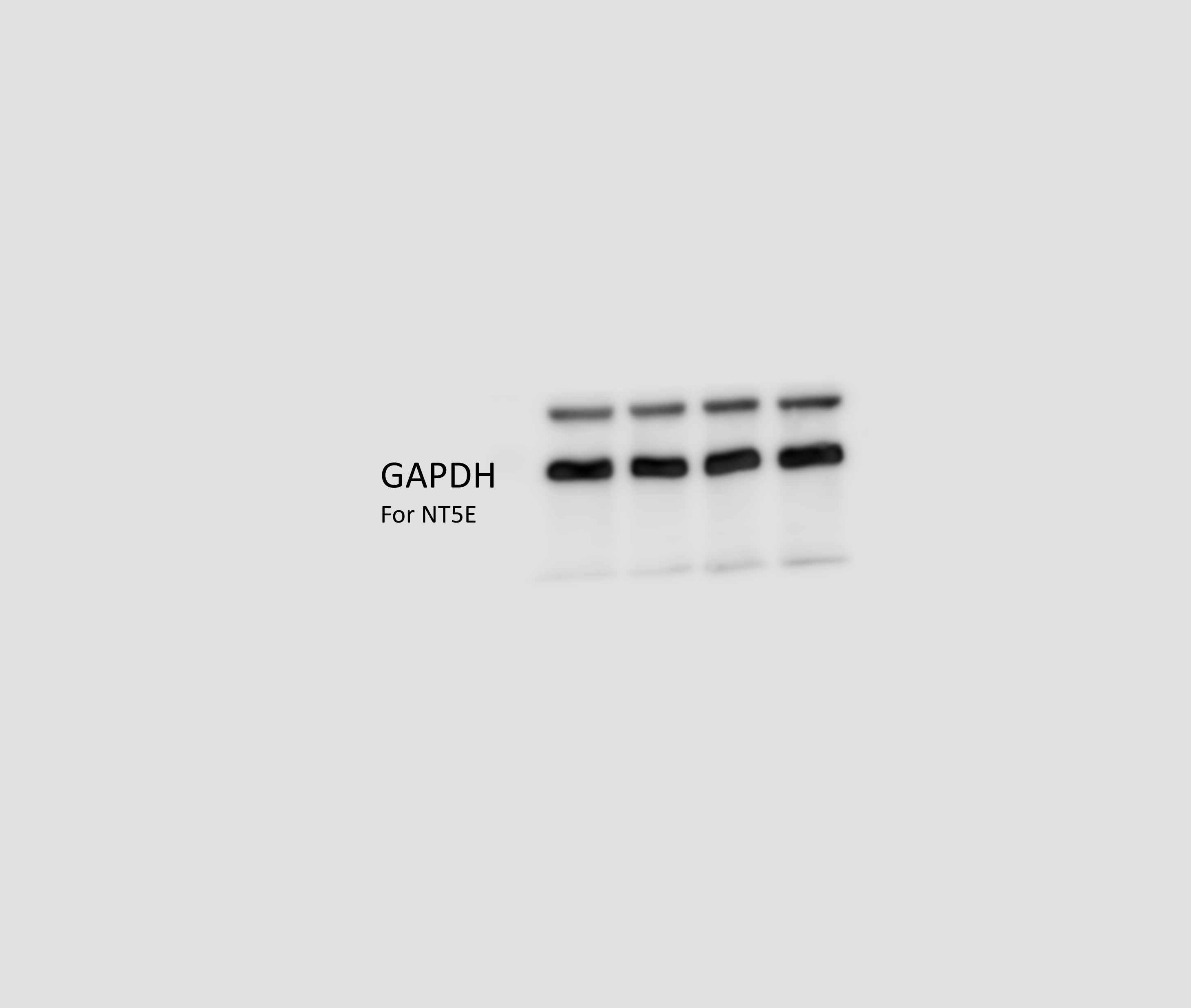
GAPDH
For NT5E

CD81

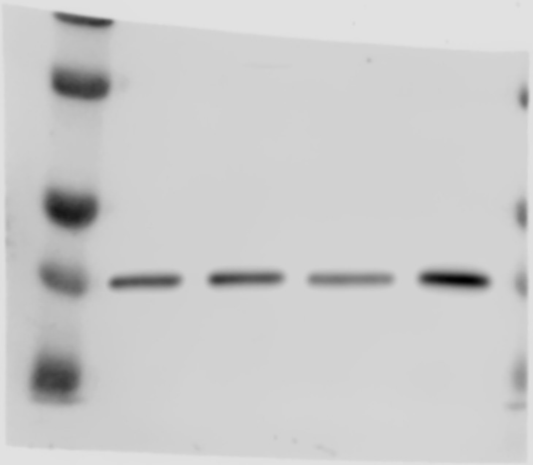

HSC70

For CD81

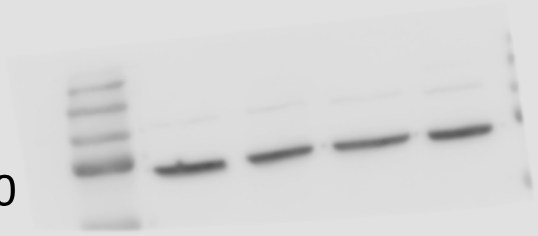

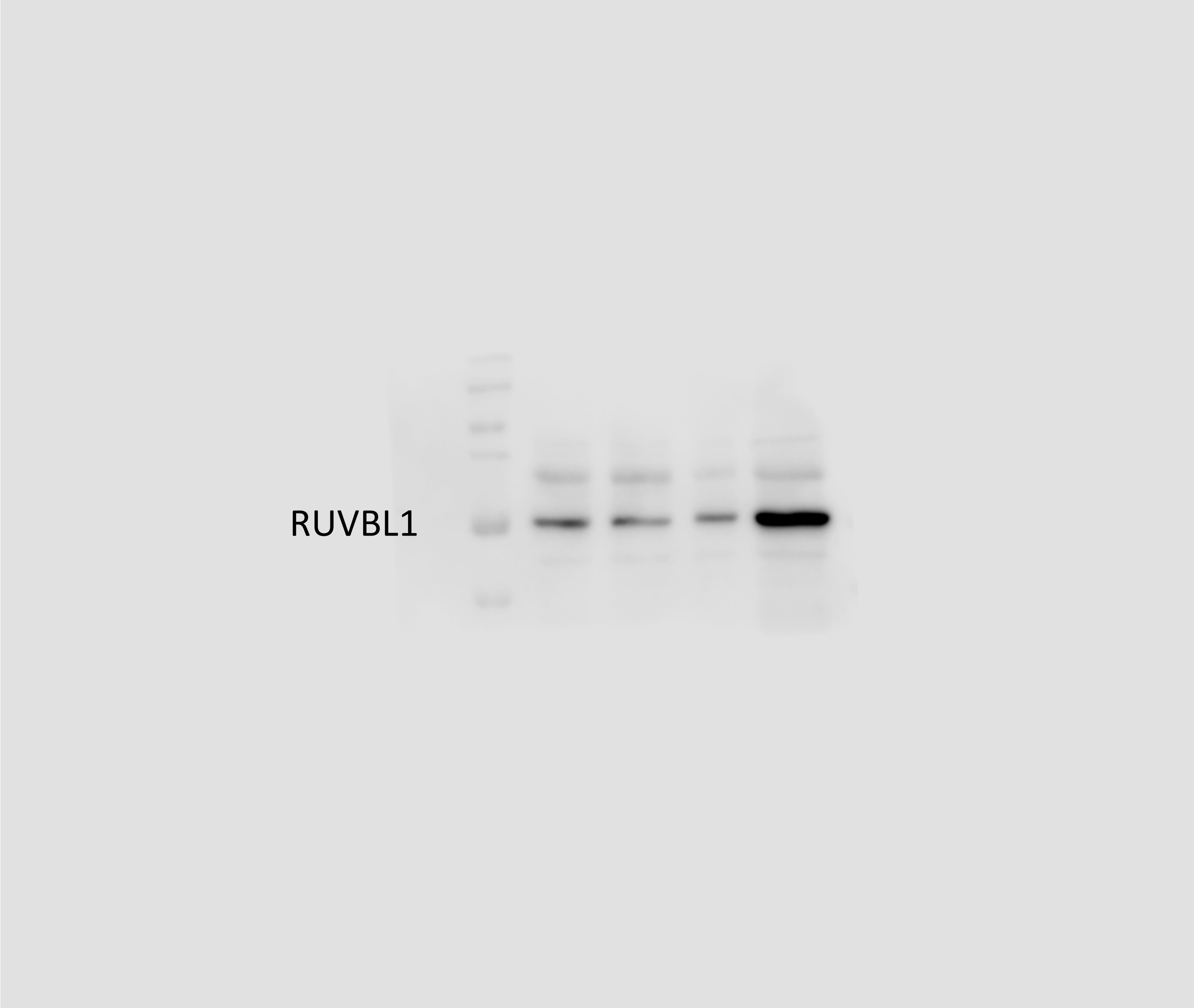
RUVBL1

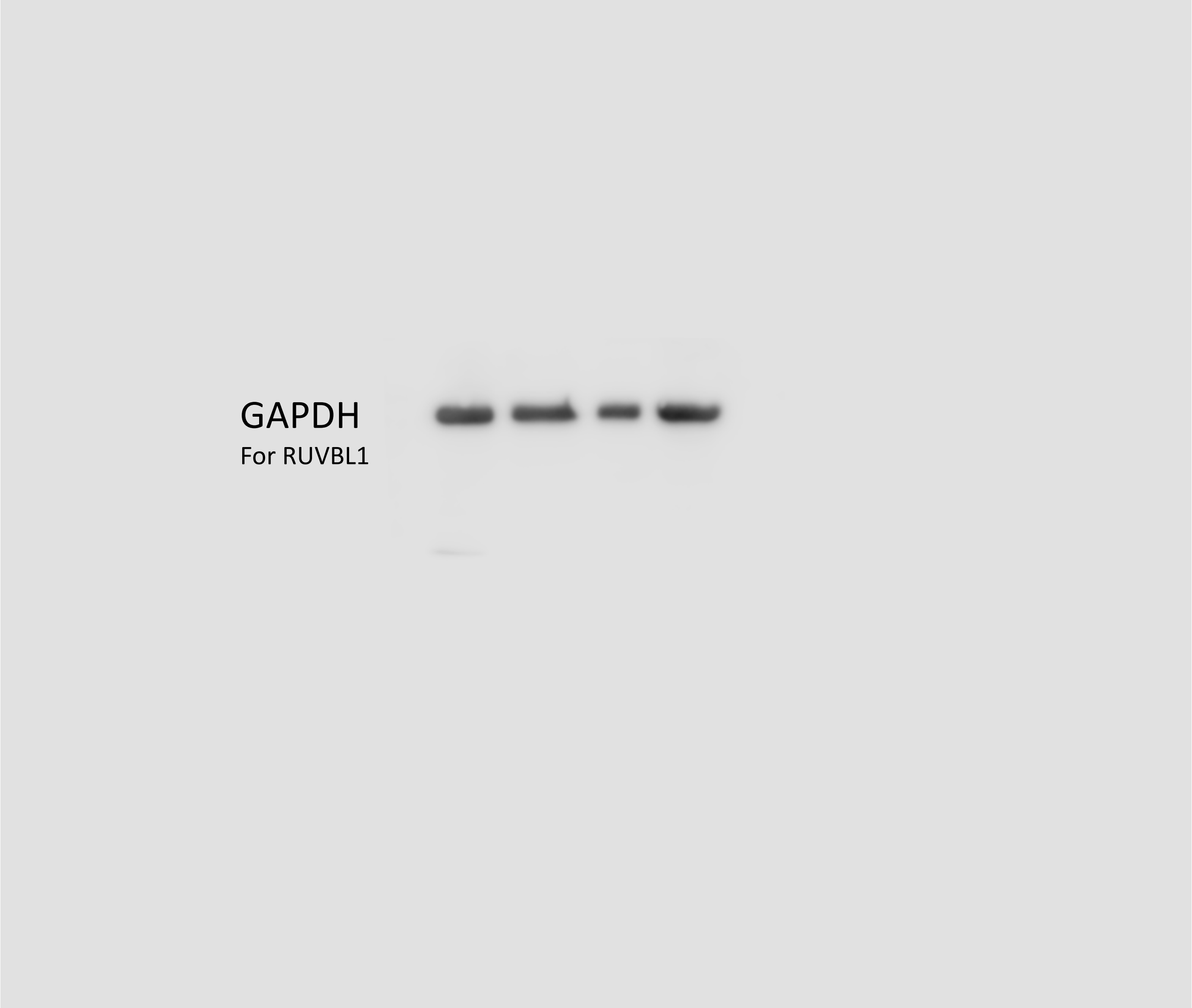
GAPDH
For RUVBL1

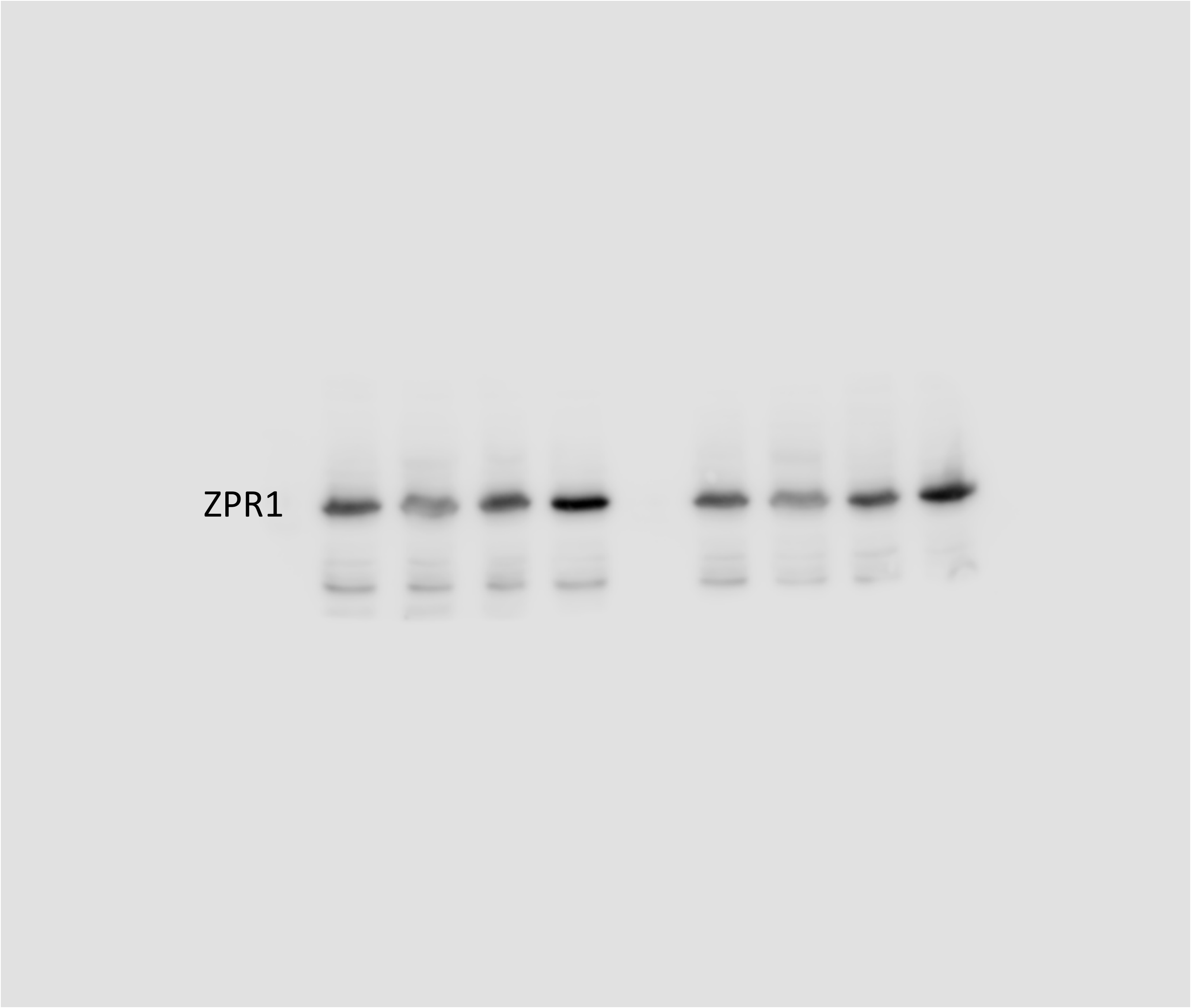
ZPR1

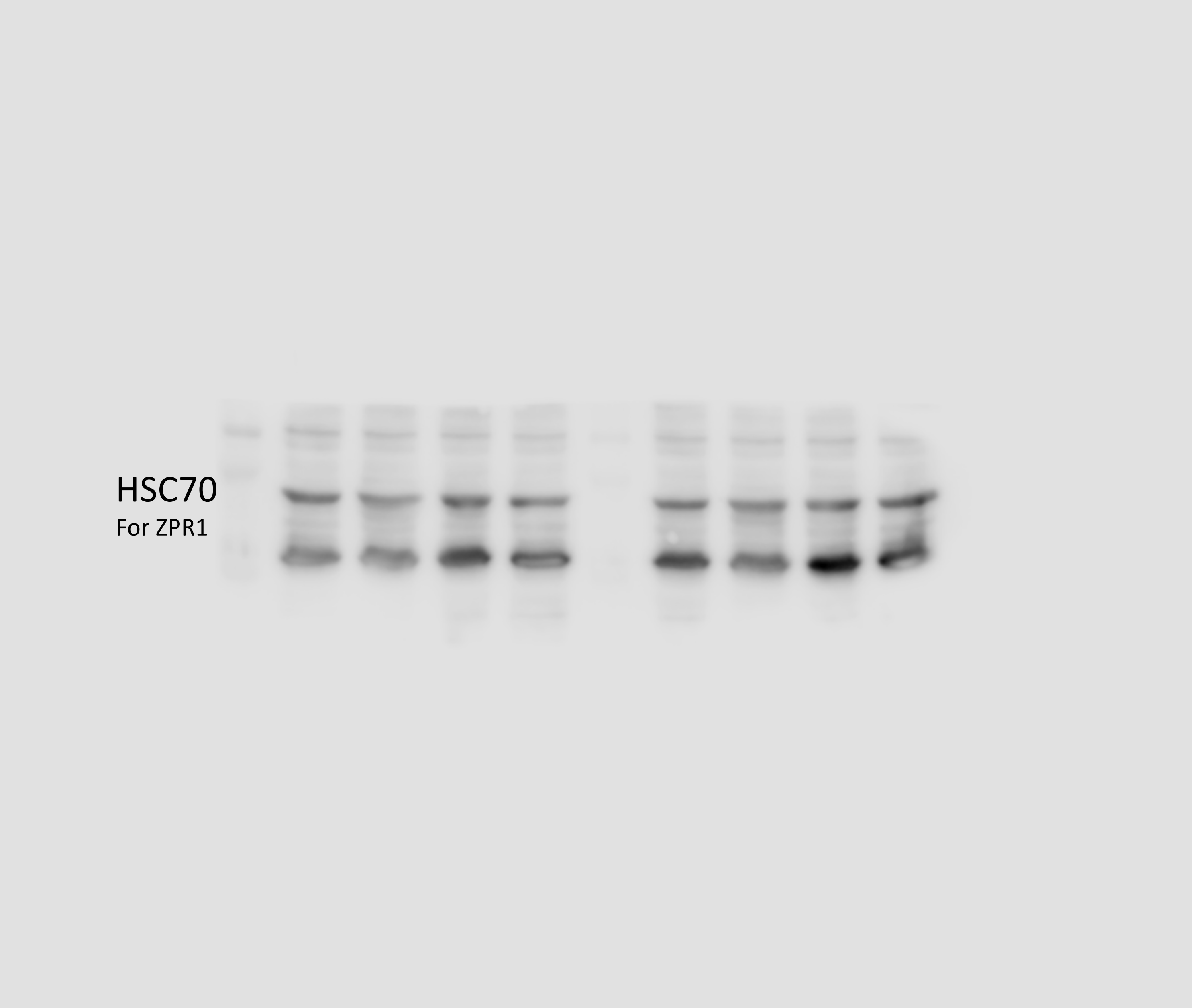
HSC70
For ZPR1

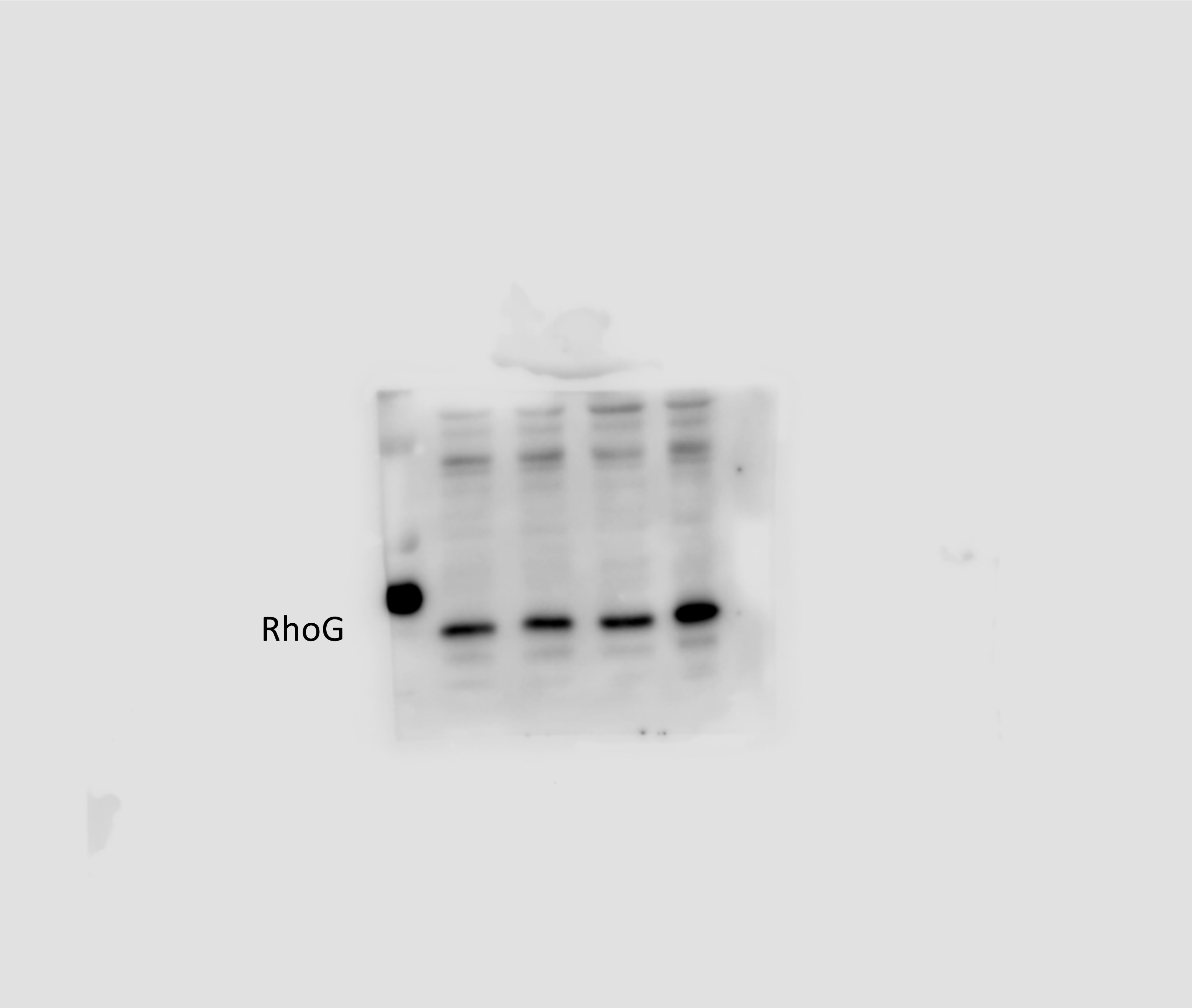
RhoG

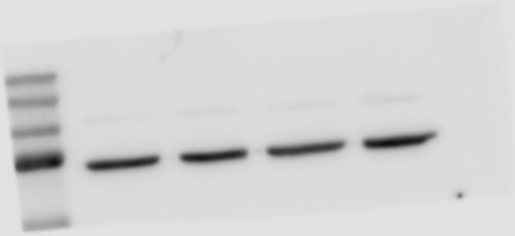

HSC70

For RhoG

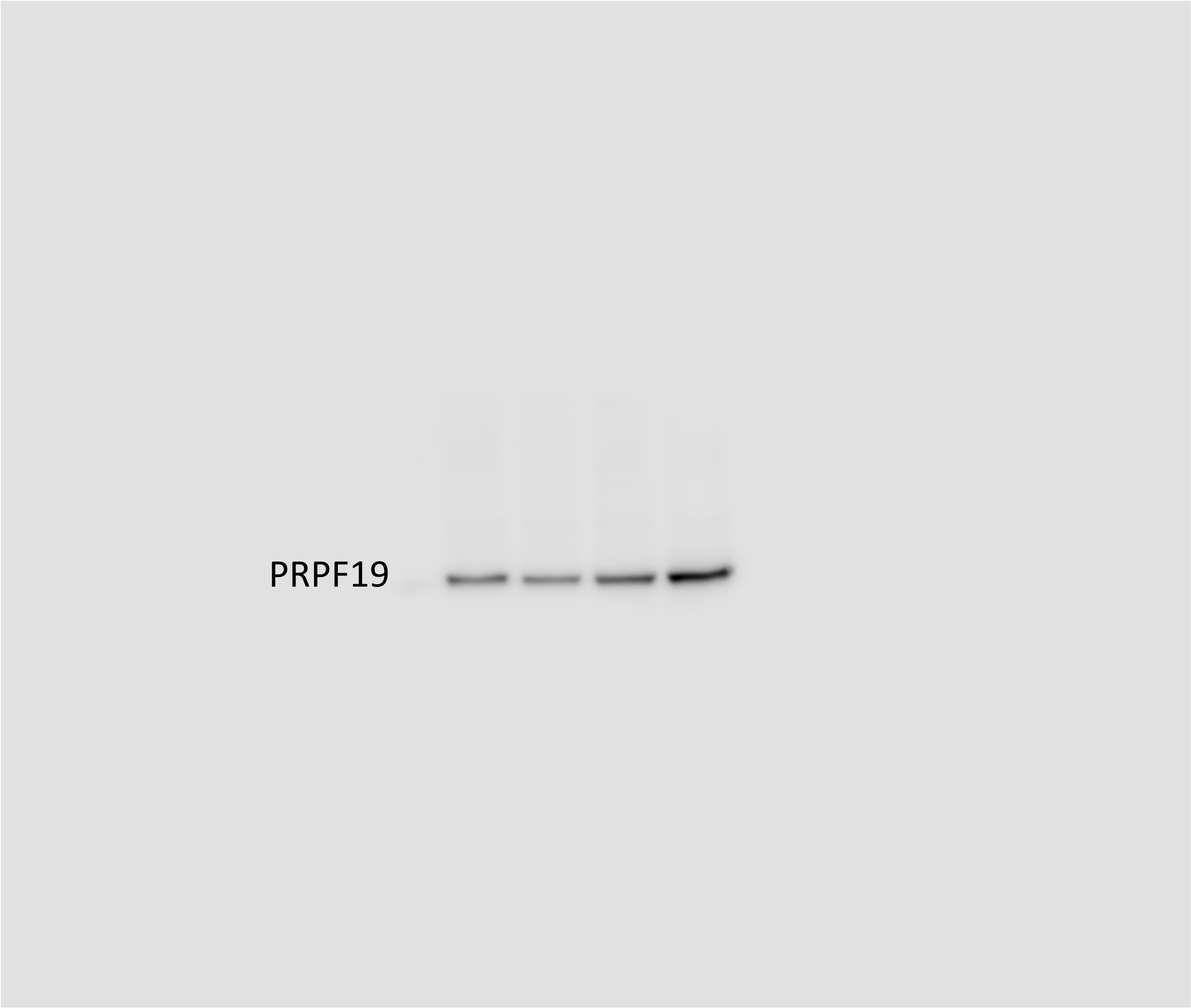
PRPF19

HSC70

For PRPF19

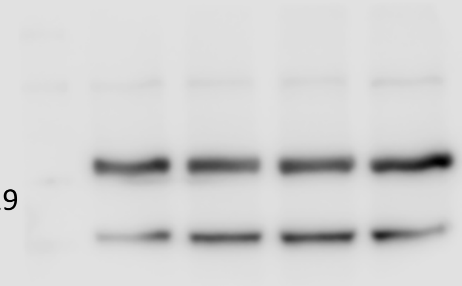

Supplement: Supplementary file 1 [file cancers-14-01968-s001.zip › supplementary/Figure_S5.pdf]
