# Supplementary figures and images for: Combined Alcohol Exposure and KRAS Mutation in Human Pancreatic Ductal Epithelial Cells Induces Proliferation and Alters Subtype Signatures Determined by Multi-Omics Analysis
Source: Cancers (Basel). 2022 Apr 13;14(8):1968. doi: 10.3390/cancers14081968 (PMC9027648; doi:10.3390/cancers14081968)

Figure S3.

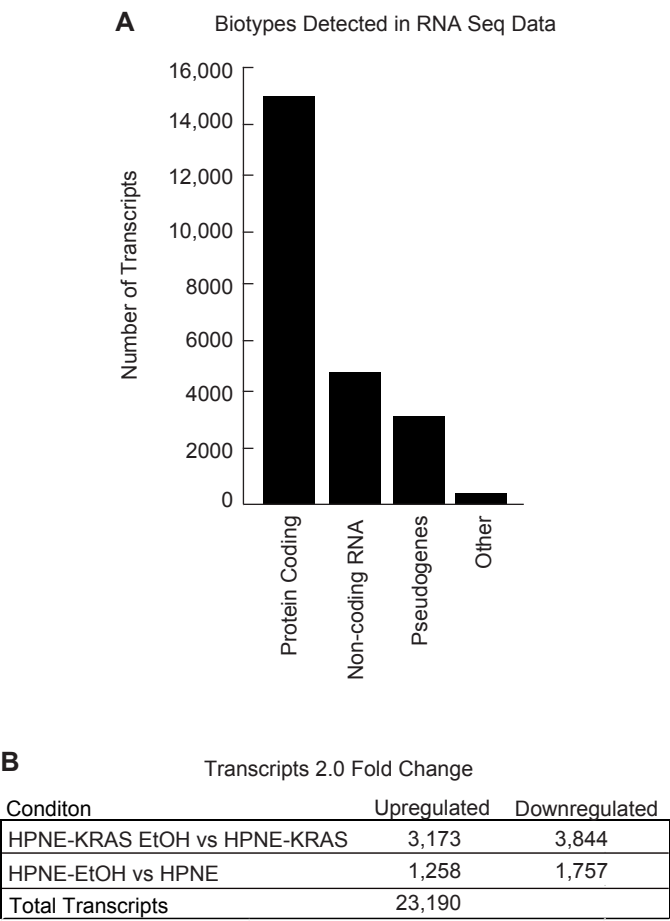

Supplement: Supplementary file 1 [file cancers-14-01968-s001.zip › supplementary/Figure_S3.pdf]

Figure S4.

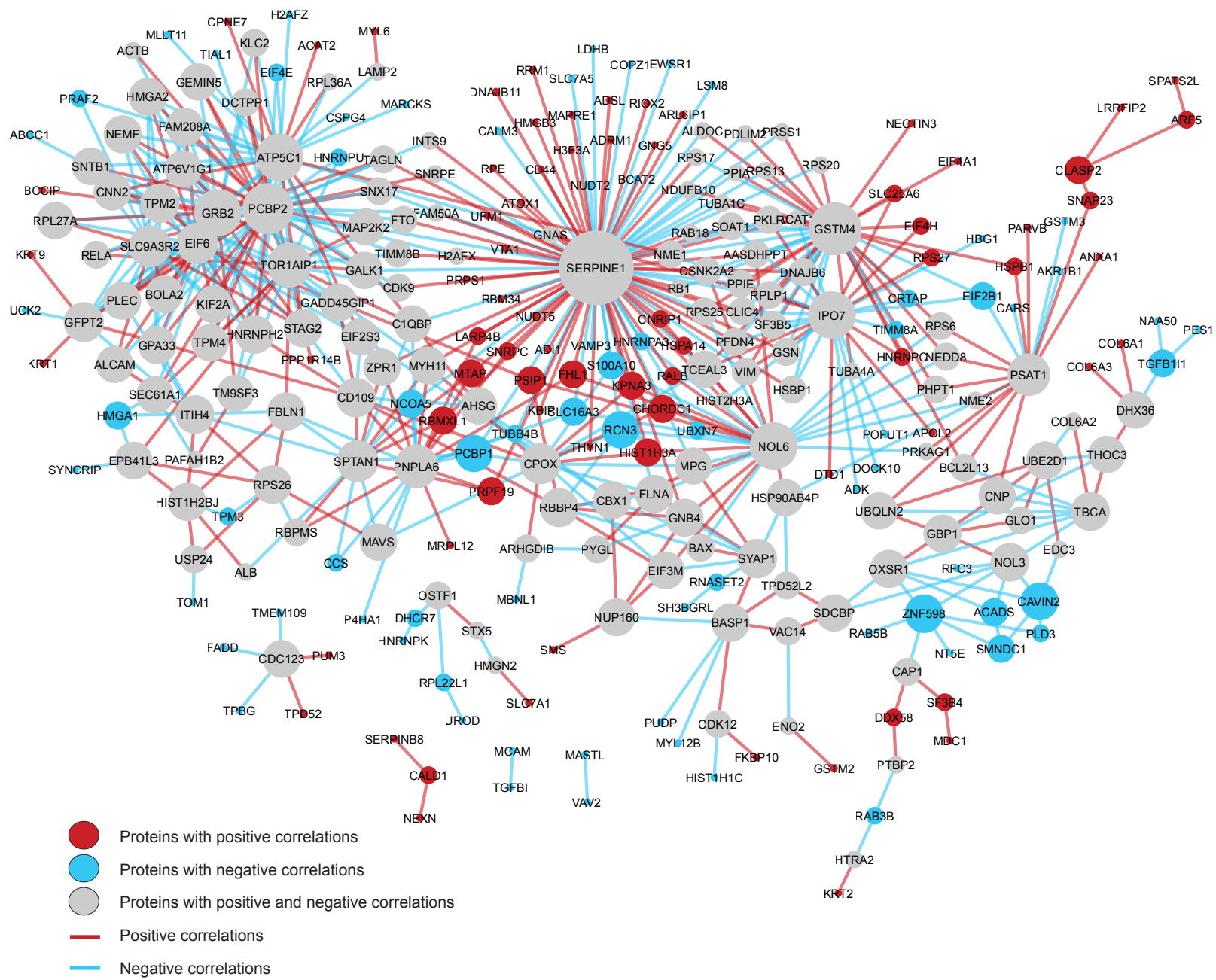

Supplement: Supplementary file 1 [file cancers-14-01968-s001.zip › supplementary/Figure_S4.pdf]
